# Nutrition and reproductive potential of women in low- and middle-income countries: a systematic review and meta-analysis

**DOI:** 10.1136/bmjgh-2024-015713

**Published:** 2025-04-02

**Authors:** Dongqing Wang, Christine H Nguyen, Anahita Asghari-Kamrani, Uttara Partap, Iqbal Shah, Wafaie W Fawzi

**Affiliations:** 1 Department of Global and Community Health, College of Public Health, George Mason University, Fairfax, Virginia, USA; 2 Department of Global Health and Population, Harvard T.H. Chan School of Public Health, Boston, Massachusetts, USA; 3 Department of Nutrition, Harvard T.H. Chan School of Public Health, Boston, Massachusetts, USA; 4 Department of Epidemiology, Harvard T.H. Chan School of Public Health, Boston, Massachusetts, USA

**Keywords:** Systematic review, Randomised control trial, Cohort study, Nutrition, Global Health

## Abstract

**ABSTRACT:**

**Introduction:**

Nutrition plays a critical role in key physiological processes related to reproduction. However, there is limited understanding of the impact of nutritional factors and interventions on the reproductive outcomes of women in low- and middle-income countries (LMICs).

**Methods:**

This systematic review and meta-analysis aimed to synthesise evidence regarding the impact of nutritional factors and interventions on the reproductive outcomes of women in LMICs. Outcomes of interest included fertility and fecundity, menarche and menstrual disorders, miscarriage, stillbirth and live birth. Randomised controlled trials (RCTs) and non-randomised intervention studies with nutritional interventions, and observational cohort studies with nutritional factors, were included. Study selection, data extraction and risk of bias assessment were independently completed by two reviewers. A narrative synthesis of included studies was conducted, and meta-analyses were conducted when feasible.

**Results:**

Systematic search identified 180 studies, including 47 intervention studies and 133 observational cohort studies. From RCTs, there was no clear evidence for an effect of prenatal multiple micronutrient supplementation on the risk of miscarriage (8 RCTs; risk ratio (RR): 0.87; 95% CI 0.75, 1.02; moderate certainty of evidence) or stillbirth (15 RCTs; RR: 0.86; 95% CI 0.73, 1.02; low certainty of evidence). From observational cohort studies, preconceptional obesity was associated with a greater risk of miscarriage (12 studies; RR: 1.27; 95% CI 1.10, 1.47; very low certainty of evidence) and stillbirth (4 studies; RR: 1.66; 95% CI 1.28, 2.14; very low certainty of evidence). Any anaemia during pregnancy was associated with a greater risk of stillbirth (10 studies; RR: 1.26; 95% CI 1.01, 1.58; very low certainty of evidence).

**Conclusion:**

This review highlights the importance of ensuring preconceptional nutrition and preventing anaemia during pregnancy for favourable reproductive outcomes. This review calls for randomised controlled trials to evaluate the effectiveness of preconceptional and prenatal interventions on these outcomes.

**PROSPERO registration number:**

CRD42023395937.

**WHAT IS ALREADY KNOWN ON THIS TOPIC**

- There is limited understanding of how nutritional factors affect measures of reproductive outcomes of women, such as fertility and fecundity, menstruation, miscarriage and stillbirth, in low- and middle-income countries.

**WHAT THIS STUDY ADDS**

- This systematic review and meta-analysis show that the achievement of normal weight before conception may protect against miscarriage and stillbirth and that the absence of anaemia during pregnancy may protect against stillbirths.

**HOW THIS STUDY MIGHT AFFECT RESEARCH, PRACTICE OR POLICY**

- This review highlights the need for randomised controlled trials to evaluate the effectiveness of preconceptional and prenatal nutritional interventions on reproductive outcomes.

## Introduction

 Female reproductive outcomes encompass the physiological capacity to conceive, sustain a pregnancy and deliver a liveborn infant. This multifaceted concept includes a range of inter-related outcomes, such as fertility and fecundity, which reflect the ability to conceive; menarche and menstrual disorders, which are key indicators of reproductive health; and adverse pregnancy outcomes, such as miscarriage and stillbirth, which represent critical endpoints in the reproductive process.

One in six people globally is affected by infertility, the inability to have a child, at some point in their life.^1^ In low- and middle-income countries (LMICs), it is estimated that 16.5% of the population have ever experienced infertility in their life.^1^ Sub-Saharan Africa, in particular, is impacted by a high prevalence of secondary infertility following a previous pregnancy.^2^,^4^ Infertility, however, has remained a neglected issue in LMICs.^4^ Among women who do conceive, the risk of miscarriage and stillbirth remains a serious issue worldwide. Miscarriage represents the loss of a pregnancy before the fetus reaches viability. An estimated 23 million miscarriages occur every year globally.^5^ A stillbirth is a baby born with no signs of life after a given threshold of birth weight or gestational age. In 2021, an estimated 3.04 million babies were stillborn at 20 weeks’ gestation or longer, with South Asia and sub-Saharan Africa jointly accounting for 77% of the global burden of stillbirth.^6^ Infertility, miscarriage and stillbirth have a multitude of physical, psychological and economic consequences for women, their households and society.^4 5 7 8^

Nutrition plays a critical role in key physiological processes related to reproduction, such as oocyte and embryo quality, blood volume, hormonal and metabolic conditions, embryo development, placenta development, fetal oxygenation and the physical ability to engage in sexual activities.^9^,^14^ The impact of nutritional interventions on birth outcomes, such as preterm birth, small-for-gestational-age birth and low birth weight, has been extensively documented.^15^,^19^ Malnutrition in all its forms, including undernutrition, overweight or obesity, and micronutrient deficiencies, can have adverse impacts on women’s abilities to conceive and give birth to live and healthy offspring.^20^,^22^ However, there is limited understanding of how nutritional factors affect measures of reproductive outcomes of women, such as fertility and fecundity, menstruation, miscarriage and stillbirth.

The impact of nutritional factors on the reproductive outcomes of women has garnered interest in recent years. Previous reviews on this topic^23^,^25^ included studies in high-income countries. Nutritional status, dietary practice, social norms and food environment differ substantially between high-income countries and LMICs. As the literature on the association between nutritional factors and human reproduction grows in LMICs, there is a critical need to systematically review evidence regarding the impact of nutritional factors on reproductive outcomes in LMICs. Such syntheses will inform the design and implementation of interventions, programmes and policies appropriate for low-resource countries to improve reproductive outcomes.

In this systematic review and meta-analysis, we aimed to summarise the epidemiological evidence on the link between nutritional factors and reproductive measures of women from intervention studies and observational cohort studies in LMICs. For intervention studies, we included studies that provided any nutritional intervention such as micronutrient supplementation, macronutrient and food supplementation, educational and behavioural interventions, and medical procedures. For observational cohort studies, we included nutritional factors such as food and nutrient intake and anthropometry. The reproductive outcomes included in this review were fertility and fecundity, menstruation, miscarriage and stillbirth.

## Methods

### Eligibility criteria

**Figure 1:**
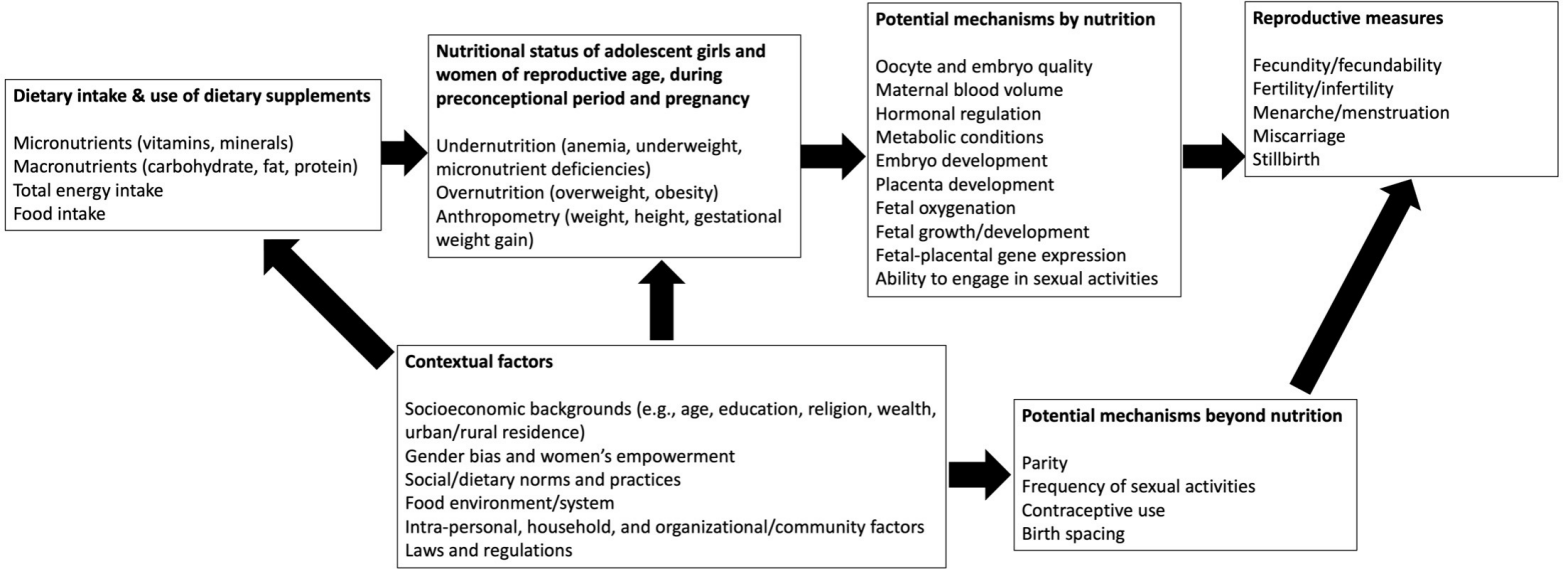
Conceptual framework outlining the impact of nutritional factors on reproductive outcomes of women.

The eligibility criteria are described based on study design, study setting, participants, interventions and exposures, comparator, and outcomes. The conceptual framework informing the review is provided in figure 1.

### Study design

We included the following study designs in this review.

1. Randomised controlled trials (RCTs), which could be individually randomised or cluster randomised.
2. Non-randomised intervention studies, including quasi-experimental designs, controlled before-after studies and interrupted time series studies. We included non-randomised intervention studies only if they incorporated a separate comparator group observed contemporaneously with the intervention group (eg, quasi-experimental designs with a control group), as opposed to relying solely on historical controls or preintervention data in a before-and-after study.
3. Observational cohort studies, including prospective cohort studies or retrospective cohort studies.
4. The included studies could be research studies or evaluations of population-based programmes.

### Study setting

We focused on studies conducted in a low-income, lower-middle-income or upper-middle-income economy defined by the World Bank country classification for the 2023 fiscal year. Multi-country studies with LMICs and high-income countries were included if results specific to the LMICs were reported.

### Participants

Outcomes measured among women of reproductive age (ie, 15–49 years) were included. Additionally, nutritional status and interventions during preconception may impact women’s health outcomes later in life.^26^ Preconception includes any period before conception, including during infancy, childhood, adolescence and adulthood. Therefore, the study population of interest encompassed the following groups.

1. Women of reproductive age (15–49 years), including older adolescent girls (ie, 15–19 years).
2. Females aged less than 15 were followed up longitudinally and had their outcomes ascertained during 15–49 years.
3. The study could be conducted among generally healthy women or exclusively among women with pre-existing health conditions (eg, overweight and obesity). Studies conducted exclusively among couples with a history of recurrent pregnancy loss or prior difficulty achieving and maintaining pregnancy were also included.
4. Studies focusing on the impact of in-utero nutritional status of the index female on her subsequent reproductive outcomes were not included so that we could focus the review on direct nutritional factors and interventions during the preconceptional and reproductive years.

While women of reproductive age, adolescent girls and younger girls followed longitudinally into reproductive have physiological differences, their inclusion in the same systematic review aligns with the broader aim of understanding how nutritional status across different life stages impacts reproductive outcomes.

### Interventions and exposures

For intervention studies, we included studies that provided any nutritional intervention aimed at influencing the nutritional status of women. Eligible interventions included (but were not limited to) micronutrient supplementation, macronutrient and food supplementation, educational interventions on dietary behaviours, behavioural interventions on weight management, behavioural interventions with direct implications on nutritional status (eg, physical activity interventions) and medical procedures with direct implications on nutritional status (eg, bariatric surgery). Intervention packages with both nutritional and non-nutritional interventions were also included.

For observational cohort studies, we included studies with any nutritional factors measured at any time point prior to the ascertainment of the outcome. Eligible nutritional factors included (but were not limited to) intake of specific foods or food groups, intake or status of macronutrients and micronutrients and anthropometric measures (eg, weight, height, underweight, overweight, obesity, stunting gestational weight gain).

### Comparator

For intervention studies, the comparator included (a) no intervention; (b) standard of care or routine care; or (c) placebo. For observational studies, the comparator varied based on individual studies, and it could include a one-unit increment of a nutritional indicator or the comparison of above versus below a specific cut-off for the nutritional indicator.

### Outcomes

Studies were included that reported the effect of nutritional interventions on, or the associations of nutritional factors with, at least one of the reproductive measures.

1. Fertility: eligible outcomes included (but were not limited to) conception, timing to pregnancy, fecundity, fecundability, fertility and infertility.
2. The onset of menarche: studies that ascertained the onset of menarche among girls younger than 15 years were also included, as the onset of menarche may occur before 15 years.
3. Menstrual disorders such as amenorrhoea, oligomenorrhoea and polymenorrhoea. Studies of symptoms, severity and prognosis of menstrual disorders exclusively among patients of the disorders were excluded.
4. Miscarriage is defined as the loss of pregnancy before 28 weeks of gestation; studies that used other definitions of miscarriage were also included.
5. Stillbirth is defined as the loss of pregnancy at or after 28 weeks of gestation; studies that used other definitions of stillbirth were also included.
6. Live birth, frequently defined as the proportion of all in vitro fertilisation cycles or embryo transfers that result in the birth of a living child.

### Exclusion criteria

We excluded the following types of records.

1. Editorials, commentaries, opinions and review articles.
2. Protocol papers and study registrations.
3. Conference abstracts and posters.
4. Studies reported in languages other than English.

### Search methods

Four electronic databases, including PubMed, EMBASE, Web of Science and the Cochrane Library, were searched from the inception of each database through 9 February 2023, with no restrictions placed on publication dates (online supplemental tables 1–4). We also screened reference lists of selected articles or reports for additional papers. We did not apply date restrictions to the search strategy to capture the full breadth of available evidence and ensure inclusivity given the limited evidence from LMICs on nutrition and reproductive outcomes.

### Study selection

We used the Covidence platform (Veritas Health Innovation, Melbourne, Australia) to manage study selection, data extraction and risk of bias (ROB) assessment. Titles and abstracts were screened by two reviewers independently, with conflicts resolved by discussion involving a third reviewer. Full texts of the remaining articles were retrieved and reviewed for inclusion independently by two of the reviewers, with conflicts resolved by discussion involving a third reviewer.

### Data extraction

Data extraction was conducted independently by two of the reviewers using a pre-specified form. The data extracted included general information, study characteristics, participant characteristics, interventions or exposures, outcomes, study results and main findings. Any discrepancies during data extraction were resolved by discussion involving a third reviewer.

### Assessment of ROB

For randomised trials, we assessed the ROB using the ROB-2 tool.^27^ For cluster RCTs, we used the ROB-2 tool for cluster-randomised studies.^28^ For non-randomised intervention studies, we assessed the ROB using the ROBINS-I tool.^29^ For observational cohort studies, we assessed the ROB using the ROBINS-E tool.^30^ Two reviewers independently assessed the ROB using the above tools, with disagreements resolved by a third reviewer.

### Data synthesis

We undertook a narrative synthesis of all included studies and presented the results in tables grouped by study design. We conducted meta-analyses if a consistently defined exposure-outcome association or intervention-outcome effect was reported in at least three studies. For binary outcomes, we used ORs or risk ratios (RRs) in the analysis. We used RRs when studies directly provided them. For studies that only reported ORs, we used ORs to ensure a consistent representation of the findings. We did not convert ORs to RRs (or vice versa) because this could introduce potential inaccuracies when the baseline risk is unknown or varies substantially between populations. For continuous outcomes, we used mean differences or standardised mean differences in the analysis. SEs or 95% CIs were used as measures of confidence. We conducted meta-analyses separately by RCTs, non-randomised intervention studies and observational cohort studies. We used results adjusted for potential confounders whenever available. We used fixed-effect and random-effects models with the inverse variance method to compute the pooled estimates. We used both fixed-effect and random-effects models to assess the robustness of pooled estimates. Random-effects models were used as the primary approach in reporting and interpretation given the expected heterogeneity in study populations, settings and designs. We assessed statistical heterogeneity using the I^2^ value.^31^ We used funnel plots to assess the presence of small study effects when there were at least 10 studies. We conducted meta-analyses using the Comprehensive Meta-Analysis Software V.3 (Biostat, Englewood, New Jersey, USA).^32^

### Certainty of evidence

We evaluated the certainty of evidence through the Grading of Recommendations, Assessment, Development and Evaluation (GRADE) approach using GRADEPro GDT.^33^

### Registration and reporting

This work was registered with the International Prospective Register of Systematic Reviews (PROSPERO ID: CRD42023395937). We reported this study in accordance with the Preferred Reporting Items for Systematic Reviews and Meta-Analyses 2020 guidelines.^34^

### Patient and public involvement

Patients and the public were not involved in this systematic review and meta-analysis.

## Results

### Characteristics of the included studies

**Figure 2:**
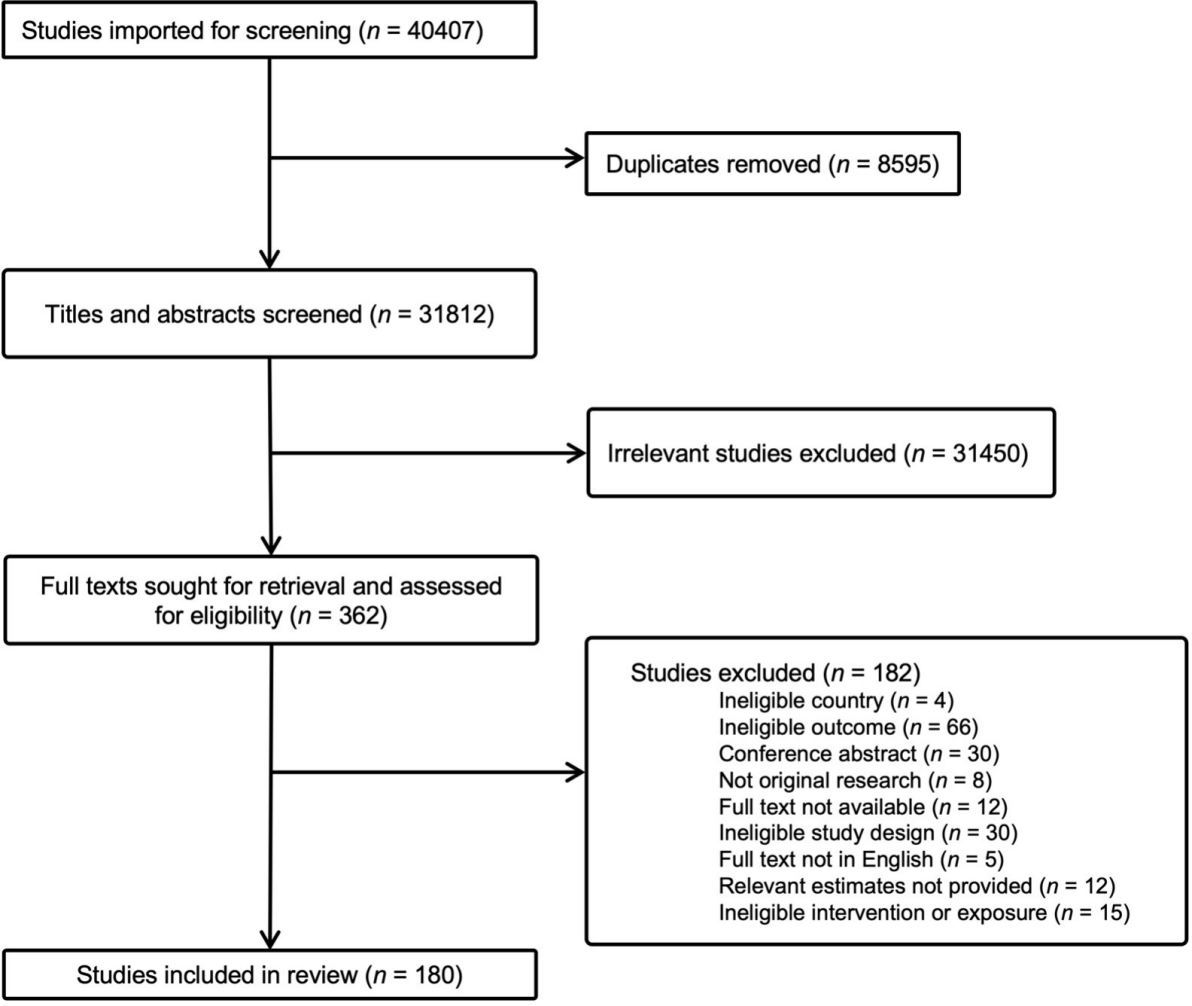
Preferred Reporting Items for Systematic Reviews and Meta-Analyses flowchart for the systematic review and meta-analysis of the impact of nutritional factors on the reproductive outcomes of women in low- and middle-income countries.

We identified 31 812 unique articles from the electronic databases, 31 450 of which were irrelevant and excluded after the title and abstract screening. Among the remaining 362 articles in the full-text review, 182 were excluded due to various exclusion criteria, resulting in 180 articles in data extraction (figure 2). These included 29 RCTs with individual randomisation, 13 cluster RCTs, 5 non-randomised intervention studies and 133 observational cohort studies.

Among the 47 intervention studies, 16 studies were conducted in sub-Saharan Africa, 9 in South Asia, 5 in Latin America and the Caribbean, 12 in the Middle East and North Africa and 5 in East Asia and the Pacific (online supplemental table 5). Intervention-outcome combinations that have been frequently studied are the effect of prenatal multiple micronutrient supplementation (MMS) on miscarriage (8 studies with quantitative estimates to enable meta-analysis) and the effect of MMS on stillbirth (15 studies) (online supplemental table 6). These intervention effects were subsequently examined in meta-analyses.

Among the 133 observational cohort studies (which included secondary, observational analyses of parent RCTs), 24 studies were conducted in sub-Saharan Africa, 24 in South Asia, 14 in Latin America and the Caribbean, 8 in Middle East and North Africa, 56 in East Asia and Pacific, 4 in Europe and Central Asia and 3 studies spanning multiple world regions (online supplemental table 7). Exposure-outcome combinations that have been frequently evaluated included preconceptional body mass index (BMI) and miscarriage (17 studies with quantitative estimates to enable meta-analysis), anaemia during pregnancy and miscarriage (4 studies), preconceptional BMI and early pregnancy loss or early miscarriage (5 studies), anaemia during pregnancy and stillbirth (17 studies), preconceptional BMI and stillbirth (7 studies), BMI during early pregnancy and stillbirth (5 studies), preconceptional BMI and biochemical pregnancy (5 studies), clinical pregnancy (15 studies), implantation rate (6 studies) and live birth rate (14 studies) (online supplemental table 8). These associations were subsequently examined in meta-analyses.

Online supplemental table 9 summarises the definitions of different outcomes in the identified studies. Early pregnancy loss is commonly defined as a biochemical pregnancy without subsequent ultrasound signs of a viable pregnancy. Early miscarriage is commonly defined as a loss of pregnancy before reaching 12 weeks of gestation; early miscarriage represents a subset of all miscarriages (ie, loss of pregnancy before reaching 28 weeks of gestation). We collapsed them as the same outcome in the analysis since they are conceptually the same construct.

### Intervention studies

#### Prenatal MMS and miscarriage/stillbirth

**Figure 3:**
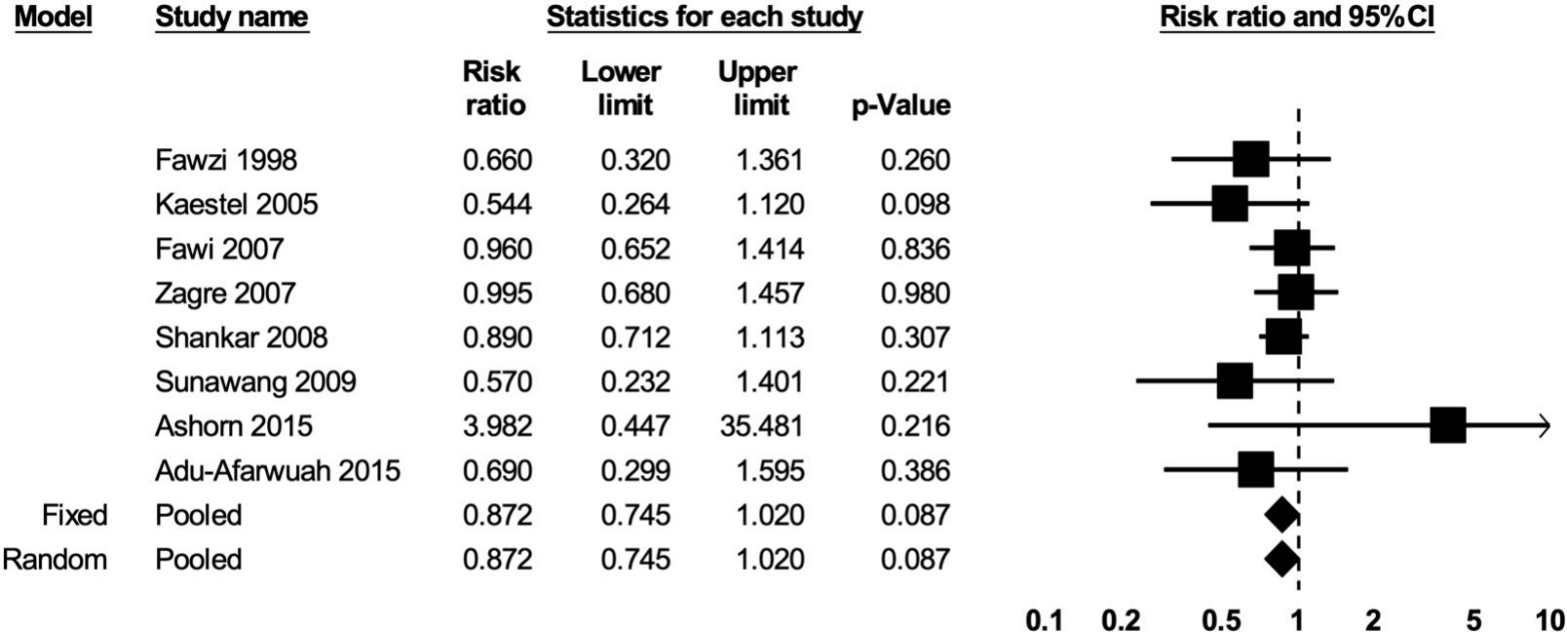
The effect of prenatal MMS on miscarriage. Heterogeneity: τ^2^=0; Q value=5.95, df=7 (p=0.55); I^2^=0%. MMS, multiple micronutrient supplementation.

**Figure 4:**
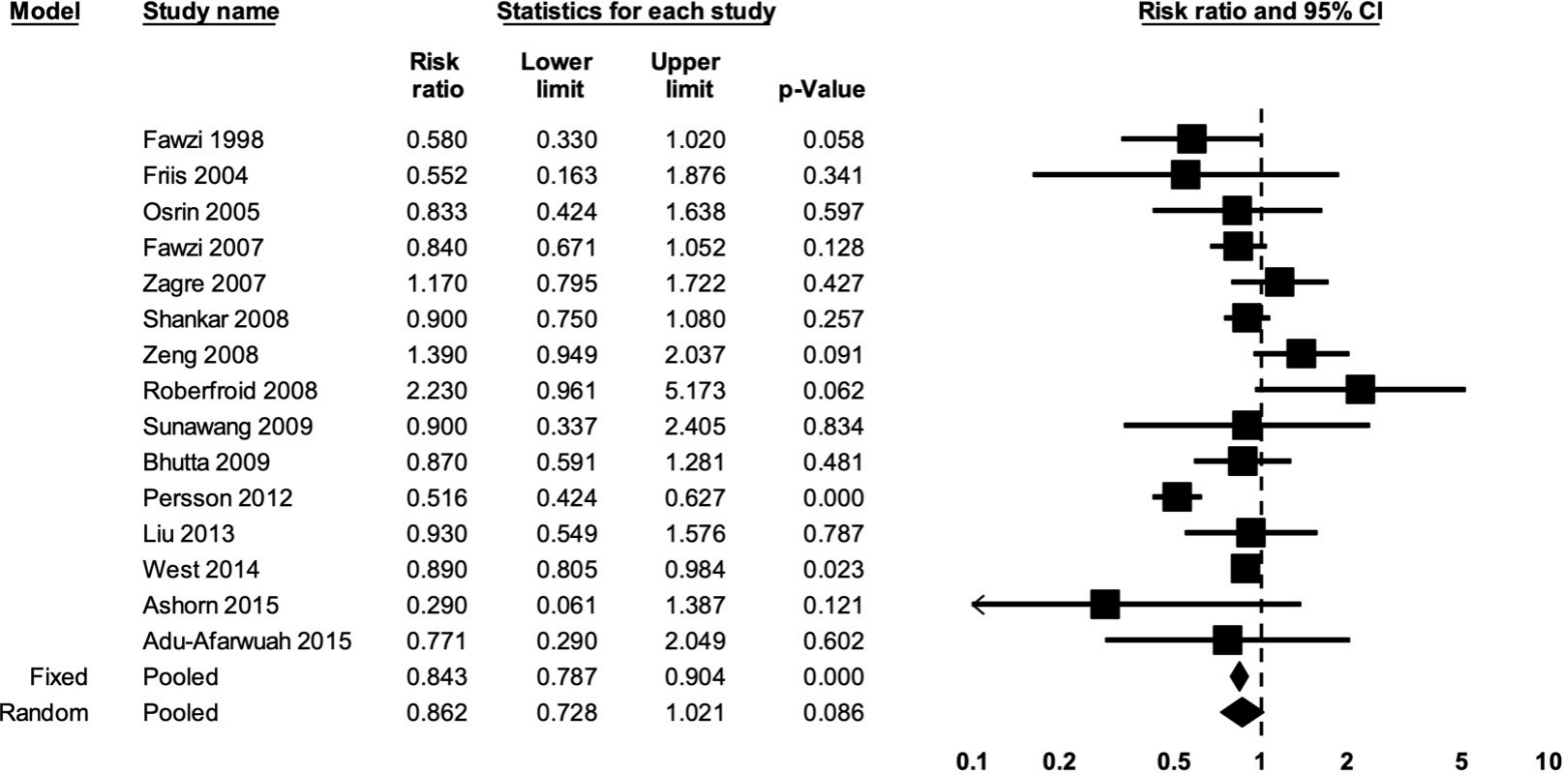
The effect of prenatal MMS on stillbirth. Heterogeneity: τ^2^=0.05; Q value=44.48, df=14 (p<0.001); I^2^=68.52%. MMS, multiple micronutrient supplementation.

Based on the random-effects model, there was no clear evidence for an effect of prenatal MMS on the risk of miscarriage compared with control (eight studies; RR: 0.87; 95% CI 0.75, 1.02; p=0.09; I^2^=0%; moderate certainty of evidence based on GRADE) (figure 3). There was no clear evidence for an effect of prenatal MMS on the risk of stillbirth compared with control (15 studies; RR: 0.86; 95% CI 0.73, 1.02; p=0.09; I^2^=69%; low certainty of evidence) (figure 4).

#### Narrative results from intervention studies

From the intervention studies, prenatal zinc,^35^,^38^ iron,^35 39^ vitamin D^40^ and vitamin A supplementation^41^,^43^ did not appear to affect the risk of miscarriage, stillbirth or fetal loss. Based on an RCT in Iran^44^ and a non-randomised intervention study in Egypt,^45^ a high dosage of folic acid supplementation of 5 mg per day from preconception or early pregnancy appeared to reduce the risk of miscarriage compared with 400–500 µg per day. Few studies have examined the effects of micronutrient supplementation on outcomes including menstrual disorders, clinical pregnancy, infertility, live birth rate and age at menarche (online supplemental tables 5 and 6).

### Observational studies

#### BMI during preconception and/or pregnancy and miscarriage/stillbirth

Underweight, normal weight, overweight and obesity were commonly defined as BMI<18.5 kg/m^2^, 18.5 to <25 kg/m^2^, 25 to <30 kg/m^2^ and ≥30 kg/m^2^, respectively. Compared with preconceptional normal weight, overweight during preconception was associated with a 9% greater risk of miscarriage (14 studies; RR: 1.09; 95% CI 1.02, 1.17; p=0.01; I^2^=40%; very low certainty of evidence) (online supplemental figure 1). Obesity during preconception was associated with a 27% greater risk of miscarriage (12 studies; RR: 1.27; 95% CI 1.10, 1.47; p=0.001; I^2^=65%; very low certainty of evidence) (online supplemental figure 2). Underweight during preconception showed no clear association with miscarriage (13 studies; RR: 1.03; 95% CI 0.93, 1.14; p=0.58; I^2^=65%; very low certainty of evidence) (online supplemental figure 3). Preconceptional overweight was associated with an 11% greater risk of early pregnancy loss or early miscarriage (RR: 1.11; 95% CI 1.02, 1.21; p=0.02; I^2^=0%; very low certainty of evidence) (online supplemental figure 4). Preconceptional obesity was associated with a 40% greater risk of early pregnancy loss or early miscarriage (RR: 1.40; 95% CI 1.20, 1.63; p<0.001; I^2^=0%; very low certainty of evidence) (online supplemental figure 5). There was no clear association between preconceptional underweight and the risk of early pregnancy loss or early miscarriage (online supplemental figure 6).

**Figure 5:**
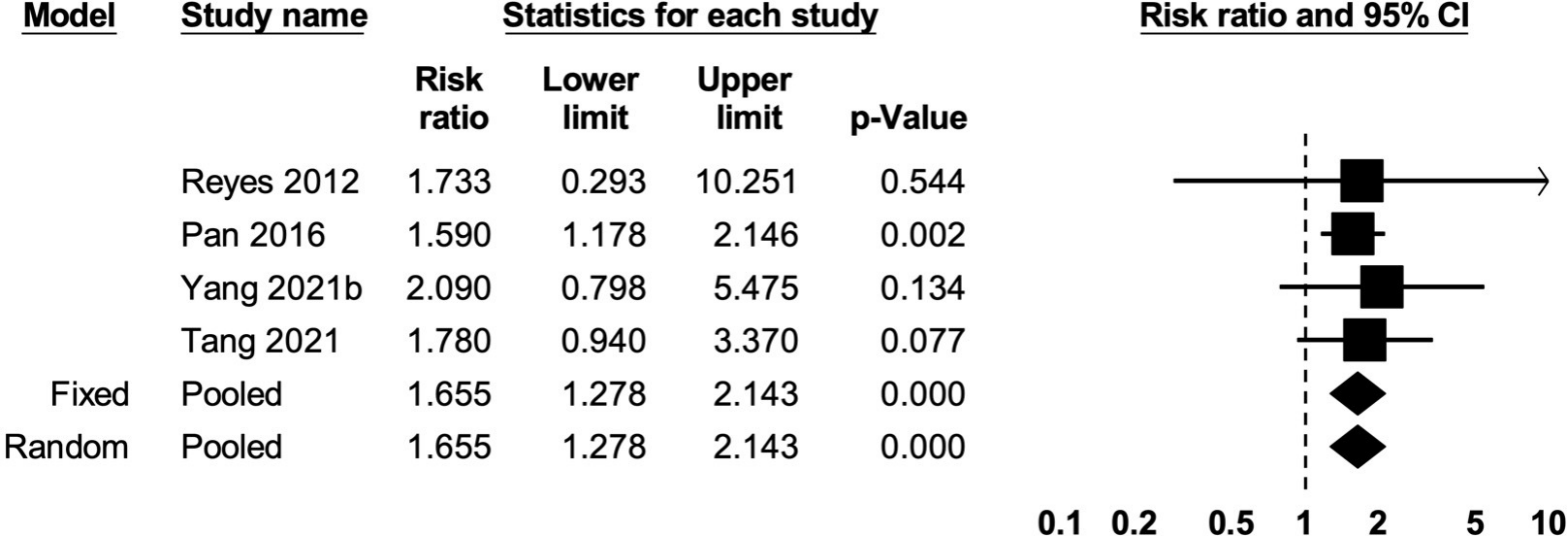
The association between obesity during preconception and stillbirth. Heterogeneity: τ^2^=0; Q value=0.35, df=3 (p=0.95); I^2^=0%.

Compared with normal weight before pregnancy, preconceptional overweight was associated with an 18% greater risk of stillbirth (five studies; RR: 1.18; 95% CI 1.03, 1.34; p=0.01; I^2^=0%; very low certainty of evidence) (online supplemental figure 7). Preconceptional obesity was associated with a 66% greater risk of stillbirth (four studies; RR: 1.66; 95% CI 1.28, 2.14; p<0.001; I^2^=0%; very low certainty of evidence) (figure 5). Underweight during preconception showed no clear association with stillbirth (five studies; RR: 0.93; 95% CI 0.79, 1.11; p=0.43; I^2^=31%; very low certainty of evidence) (online supplemental figure 8). Underweight, overweight and obesity during early pregnancy showed no clear associations with the risk of stillbirth compared with normal weight (online supplemental figures 9–11; very low certainty of evidence).

#### Anaemia during pregnancy and miscarriage/stillbirth

**Figure 6:**
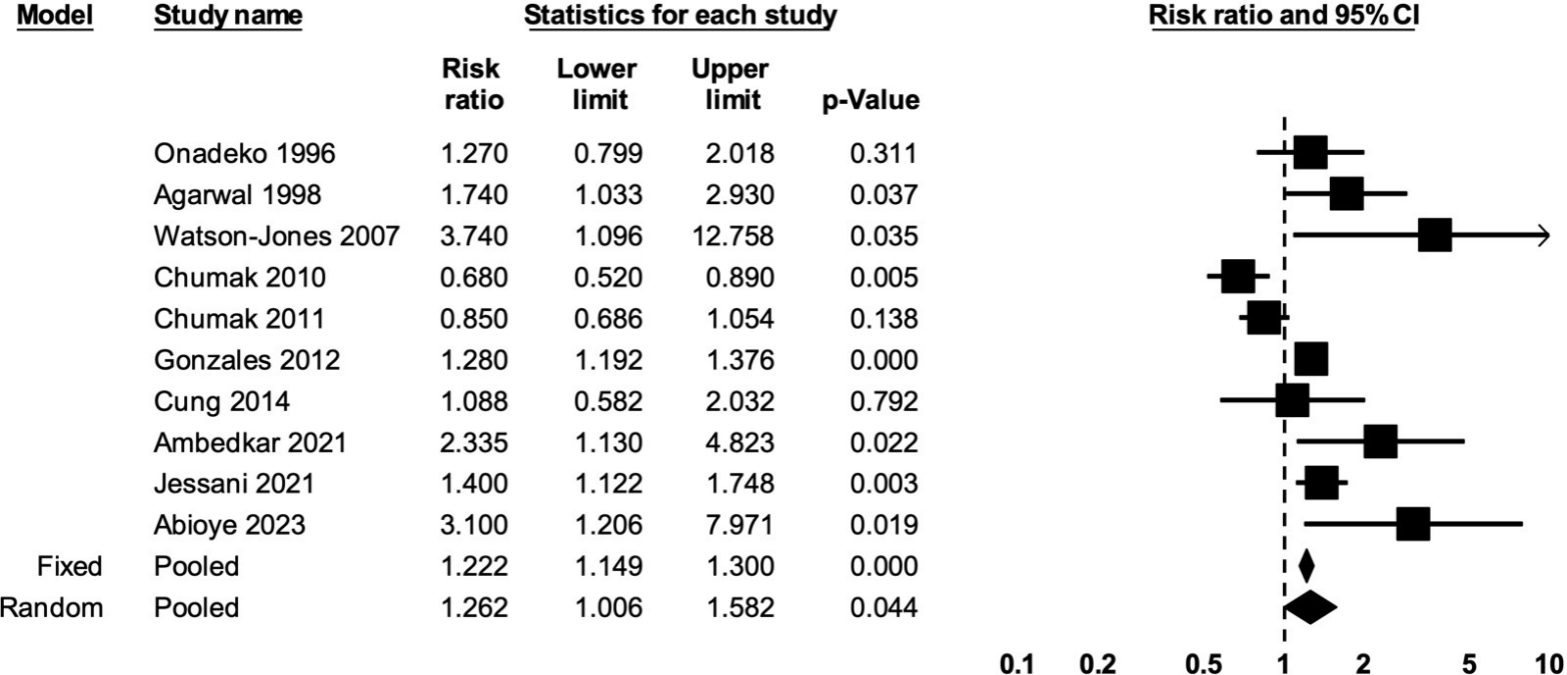
The association between any anaemia during pregnancy and stillbirth. Heterogeneity: τ^2^=0.08; Q-value=44.25, df=9 (p<0.001); I^2^=76.66%.

Any anaemia during pregnancy was associated with an 88% greater risk of stillbirth (10 studies; RR: 1.26; 95% CI 1.01, 1.58; p=0.04; I^2^=77%; very low certainty of evidence) (figure 6). Neither mild nor moderate anaemia showed clear associations with stillbirth compared with no anaemia (online supplemental figures 12 and 13); very low certainty of evidence for both). Any anaemia during pregnancy showed no clear association with the risk of miscarriage (three studies; RR: 1.63; 95% CI 0.51, 5.20; p=0.41; I^2^=64%; very low certainty of evidence) (online supplemental figure 14).

#### Preconceptional BMI and implantation, pregnancy and live birth rates

Compared with normal weight before pregnancy, preconceptional underweight was associated with 9% lower odds of implantation (three studies; OR=0.91; 95% CI 0.86, 0.96; p<0.001; I^2^=0%; very low certainty of evidence) (online supplemental figure 15), preconceptional overweight was associated with 5% lower odds of implantation (four studies; OR=0.95; 95% CI 0.91, 0.98; p=0.006; I^2^=0%; very low certainty of evidence) (online supplemental figure 16) and preconceptional obesity was associated with 41% lower odds of implantation (five studies; OR=0.59; 95% CI 0.40, 0.86; p=0.006; I^2^=65%; very low certainty of evidence) (online supplemental figure 17). Preconceptional underweight, overweight and obesity showed no clear associations with biochemical pregnancy, commonly defined as pregnancy confirmed by the presence of serological human chorionic gonadotropin (online supplemental figures 18–20; very low certainty of evidence). Preconceptional underweight, overweight and obesity also showed no clear associations with clinical pregnancy rate, defined as pregnancy confirmed by ultrasound (online supplemental figures 21–23; very low certainty of evidence). Preconceptional underweight and obesity had no clear associations with live birth rates (online supplemental figures 24 and 25; very low certainty of evidence). Preconceptional overweight was associated with 4% lower odds of live birth (OR: 0.96; 95% CI 0.93, 1.00; p=0.04; I^2^=0%; very low certainty of evidence) (online supplemental figure 26). Funnel plots for analyses with at least 10 studies were shown in online supplemental figures 27–34; no small study effects were detected based on the funnel plots.

#### Narrative results from observational studies

From the observational cohort studies, a wide array of exposures and outcomes have been studied (online supplemental tables 7 and 8). A few studies reported that overweight or obesity during early pregnancy was associated with a greater risk of miscarriage.^46^,^49^ Several studies also reported that undernutrition, such as stunting during childhood or adolescence, was associated with a later onset of menarche.^50^,^56^ In contrast, girls with overweight or obesity during childhood or adolescence might experience an earlier onset of menarche.^57 58^ Women with stunting during childhood or adolescence might reach menarche later than those without stunting or with less severe stunting.^50 51 53 55 56 59^

### Risk of bias

Among the 29 RCTs with individual randomisation, 20 had a low ROB, 8 had some concerns and 1 had a high ROB (online supplemental figure 35). The primary domain of bias for the RCTs with individual randomisation was bias arising from the randomisation process, with seven studies having some concerns and one study having a high ROB in this domain. Among the 13 cluster RCTs, 8 had a low ROB and 5 had some concerns (online supplemental figure 36). The primary domain of bias for the cluster RCTs was bias in the selection of the reported results, with seven studies having some concerns in this domain. Among the five non-randomised intervention studies, two had a low ROB, and three had a moderate ROB (online supplemental figure 37). The primary domain of bias for the non-randomised intervention studies was bias in the selection of the reported results, with three studies having some concerns in this domain. Among the 133 observational cohort studies, 19 (14.3%) had a low ROB, 56 (42.1%) a moderate ROB, 33 (24.8%) a serious ROB and 25 (18.8%) a critical ROB (online supplemental table 10). The primary domains of bias among the observational cohort studies were the lack of any attempt to control for confounding and the sufficient potential for confounding that an unadjusted result should not be considered further, which resulted in a study having a critical ROB. The GRADE table summarising the certainty of evidence is shown in online supplemental table 11. Key findings from the meta-analyses with the certainty of evidence are summarised in table 1.

**Table 1:** Summary of key findings from the meta-analyses on the impact of nutritional factors on female reproductive outcomes in low- and middle-income countries

Nutritional factor	Outcome	Certainty of evidence
Prenatal MMS	No clear effect on miscarriage (RR: 0.87; 95% CI 0.75, 1.02)	Moderate
Prenatal MMS	No clear effect on stillbirth (RR: 0.86; 95% CI 0.73, 1.02)	Low
Preconceptional overweight	Associated with a greater risk of miscarriage (RR: 1.09; 95% CI 1.02, 1.17)	Very low
Preconceptional overweight	Associated with a greater risk of early pregnancy loss/early miscarriage (RR: 1.11; 95% CI 1.02, 1.21)	Very low
Preconceptional overweight	Associated with a greater risk of stillbirth (RR: 1.18; 95% CI 1.03, 1.34)	Very low
Preconceptional overweight	Associated with lower odds of implantation (OR: 0.95; 95% CI 0.91, 0.98)	Very low
Preconceptional obesity	Associated with a greater risk of miscarriage (RR: 1.27; 95% CI 1.10, 1.47)	Very low
Preconceptional obesity	Associated with a greater risk of early pregnancy loss/early miscarriage (RR: 1.40; 95% CI 1.20, 1.63)	Very low
Preconceptional obesity	Associated with a greater risk of stillbirth (RR: 1.66; 95% CI 1.28, 2.14)	Very low
Preconceptional obesity	Associated with lower odds of implantation (OR: 0.59; 95% CI 0.40, 0.86)	Very low
Preconceptional underweight	Associated with lower odds of implantation (OR: 0.91; 95% CI 0.86, 0.96)	Very low
Any anaemia during pregnancy	Associated with a greater risk of stillbirth (RR: 1.26; 95% CI 1.01, 1.58)	Very low

MMSmultiple micronutrient supplementationRRrisk ratio

## Discussion

This systematic review and meta-analysis evaluated the impact of nutritional factors on the reproductive outcomes of women in LMICs. We found a large number of studies representing heterogeneous nutritional interventions, nutritional factors and outcomes, and evidence synthesis was feasible for relatively few effects or associations. Preconceptional overweight and obesity were associated with miscarriage and stillbirth. Any anaemia during pregnancy was associated with a greater risk of stillbirth. This review highlights the paucity of high-quality evidence from RCTs regarding preconceptional nutrition and fertility outcomes.

The lack of clear benefits of prenatal MMS on miscarriage or stillbirth is consistent with previous systematic reviews and meta-analyses.^60 61^ The WHO’s updated recommendation also states that prenatal MMS has little to no benefits on the risk of stillbirth.^16^ However, stillbirth is a rare outcome, and individual studies rarely have adequate statistical power to specifically detect the effects of interventions on stillbirth. While our study and previous meta-analyses^60 61^ showed results that barely failed to achieve statistical significance, the 95% CIs of the estimates do not preclude important benefits of prenatal MMS on stillbirth. Further, prenatal MMS reduces the risks of small-for-gestational-age births and low birth weight and may also prevent preterm birth compared with iron and folic acid supplementation, which leads to calls for the switch to MMS as the standard of antenatal care.^60^,^62^ Discourse regarding the optimal composition and dosage of the micronutrients provided in MMS is also ongoing.

There is evidence that preconceptional underweight, overweight or obesity may be associated with greater risks of adverse pregnancy outcomes, including early pregnancy loss, miscarriage and stillbirth; however, the certainty of this evidence is very low. It is also worth noting that the existing studies on preconceptional nutrition have predominantly focused on the narrow window immediately before conception, often among women seeking assisted reproductive technologies. There is a lack of evidence regarding the impact of nutritional status at earlier life stages, such as adolescence on reproductive outcomes. The specific mechanisms of how underweight, overweight or obesity adversely affect fertility and reproductive outcomes are unclear and likely complex. Potential mechanisms for the adverse effects of overweight and obesity may include the lipotoxic effects that reduce oocyte quality,^10 11^ obesity-induced anovulation,^13^ impaired embryo development and implantation^10^ or the presence of underlying medical conditions such as polycystic ovary syndrome.^13^ Conversely, underweight may impact reproductive outcomes by compromising oocyte maturation and disrupting embryo-endometrial interactions and implantation.^12^ There is also evidence that anaemia during pregnancy may be associated with a greater risk of stillbirth, yet the certainty of this evidence is also very low. Potential pathways through which micronutrient deficiencies such as iron-deficiency anaemia affect female reproduction include delayed menarche^9^ and impaired cellular proliferation and placental development.^14^ Maternal nutritional status at the time of conception may be more crucial for fetal development than nutrition during pregnancy, as organogenesis occurs early before many are aware of the pregnancy.^14^ These findings underline the importance of preconceptional nutrition and call for interventions to attain optimal nutritional status before conception. For example, delaying conception to achieve a healthy weight should be considered for women with obesity or underweight.^13 14^

The findings on preconceptional nutrition from this review are largely from observational studies. Around 44% of the included observational cohort studies had serious or critical risks of bias, primarily due to a lack of adequate control for confounding. As a result, the certainty of evidence on the preconceptional nutritional status, such as BMI and anaemia, is low or very low. A recent systematic review focused on the impact of interventions during preconception and periconception to prevent low birth weight, small-for-gestational-age birth and preterm birth^26^; the review identified a scarcity of evidence on the impact of preconceptional and periconceptional interventions on these adverse pregnancy outcomes.^26^ The present work complements this recent review by examining other reproductive outcomes and again highlights the critical need for RCTs to rigorously examine the effectiveness of nutritional interventions during preconception (including during the postpartum period between the current and subsequent pregnancies). In addition to interventions aimed at directly improving nutritional status, educational and family planning interventions and policies are also crucial for preventing early marriage, reducing adolescent pregnancy and achieving appropriate birth intervals.^63 64^ Such family planning interventions ultimately also contribute to improved nutritional status which in turn enables optimal outcomes for future pregnancies.^19 65^

Women and girls in LMICs face considerable barriers to achieving adequate nutrition due to cultural norms and gender inequalities, which often prioritise men’s and boys’ nutritional needs.^66^ Holistic interventions must consider these structural challenges and adopt gender-sensitive approaches to ensure equitable access to nutrition. Advancing gender equality through women’s empowerment will complement nutritional interventions and foster mutually reinforcing benefits for nutrition and reproductive health outcomes.

This review highlights several gaps that should be addressed in future research. First, there is a dearth of RCTs that evaluated preconceptional nutritional interventions compared with the more extensive research on antenatal nutritional interventions. Second, certain nutritional factors, such as dietary patterns and micronutrient intake, remain underexplored in relation to female reproductive outcomes. Third, certain intervention-outcome combinations warrant further investigation in the context of LMICs. For example, evidence from high-income countries suggests that interventions such as omega-3 fatty acid supplementation and dietary modifications may impact reproductive health, yet similar studies in LMICs are scarce.^67^ Finally, given the critical role of nutrition in early life, there is limited evidence or age-stratified results on how nutritional factors differentially impact women of different age periods, such as among adolescents. Nutritional needs and their effects on reproductive outcomes may also vary by the duration of deficiencies. For example, acute undernutrition during adolescence may disrupt menstrual cycle regularity and delay menarche, while chronic micronutrient deficiencies, such as prolonged folate or iron deficiencies may contribute to adverse pregnancy outcomes later in life. However, few studies have examined how the cumulative effects of nutritional status across different life stages influence reproductive health trajectories. Future research should prioritise age-stratified analyses and consider the interplay between short-term versus long-term nutritional exposures to inform interventions tailored to women at different reproductive stages.

The main strengths of this systematic review and meta-analysis included the coverage of a wide array of nutritional factors and women’s reproductive outcomes from both intervention studies and observational cohort studies. The inclusion of studies without date restrictions allowed for a comprehensive synthesis of evidence. Changes in access to infertility treatment, family planning services and fertility trends in LMICs may have influenced findings over time. Future research should consider examining temporal trends to account for these evolving contextual factors. This review also has some limitations. First, some of the exposures and outcomes have not been defined consistently in the studies, which complicated the evidence synthesis. For example, while underweight, overweight and obesity have frequently been defined using the WHO cut-offs, some studies also used region-specific cut-offs. Similarly, the definitions of miscarriage, stillbirth and early pregnancy loss have not been uniformly defined in the studies included. Third, we focused on databases with broad coverage of the literature (ie, PubMed, EMBASE, Web of Science and the Cochrane Library) and did not include additional regional databases and LMIC-focused repositories. Our search strategy excluded studies written in languages other than English. These restrictions in the database and language might have led to the omission of relevant evidence.^68 69^ Future reviews should consider a broader inclusion of multilingual sources to enhance comprehensiveness. Finally, we focused on maternal nutrition and did not examine the impact of paternal nutrition. Historically, research and public health programmes have overwhelmingly framed women and girls primarily as ‘mothers-to-be’ throughout their life course, emphasising their reproductive roles while often neglecting their individual health and well-being. This framing is inherently limited and reinforces gendered expectations while overlooking the critical role of paternal health in reproduction. Paternal exposures to nutritional and environmental factors may influence sperm quality, fertility and pregnancy outcomes, yet remain under-researched.^70^ Therefore, a more inclusive programmatic approach to preconceptional care would incorporate paternal health and promote shared responsibility and gender equity in reproductive health.

## Conclusion

This systematic review provides a synthesis of the impact of nutrition on the reproductive outcomes of women in LMICs. This review underscores the critical role of optimal preconceptional and prenatal nutrition in fertility and reproduction. This work highlights the need for randomised controlled trials that rigorously evaluate the effectiveness of preconceptional and prenatal nutritional interventions on reproductive and pregnancy outcomes among women and adolescent girls in community settings.

## supplementary material

10.1136/bmjgh-2024-015713 online supplemental file 1

## Data Availability

All data relevant to the study are included in the article or uploaded as supplementary information.
