# Supplementary material for: Nutrition and reproductive potential of women in low- and middle-income countries: a systematic review and meta-analysis
Source: BMJ Glob Health. 2025 Apr 2;10(Suppl 1):e015713. doi: 10.1136/bmjgh-2024-015713 (PMC11966992; doi:10.1136/bmjgh-2024-015713)
Supplement: online supplemental file 1 [file bmjgh-10-Suppl_1-s001.pdf]

SUPPLEMENTAL MATERIALS

Model	Study name	Risk ratio	Lower limit	Upper limit	p-Value
	Zhang 2010	1.628	0.986	2.687	0.057
	Reyes 2012	0.560	0.193	1.625	0.286
	Pan 2016	1.020	0.967	1.076	0.470
	Zhou 2016	1.710	1.040	2.811	0.034
	Shen 2016	0.726	0.360	1.463	0.370
	Cai 2017	1.150	0.816	1.621	0.425
	MacKenna 2017	1.000	0.915	1.093	1.000
	Sheng 2017	2.432	0.894	6.618	0.082
	Pan 2018	0.931	0.510	1.698	0.815
	Zhang 2019	1.210	1.060	1.381	0.005
	Qu 2021	1.100	0.991	1.221	0.074
	Hu 2021	1.200	0.973	1.480	0.088
	Zheng 2022	1.066	0.897	1.266	0.467
	Gudipally 2023	0.830	0.377	1.826	0.643
Fixed	Pooled	1.055	1.016	1.095	0.006
Random	Pooled	1.090	1.017	1.167	0.014

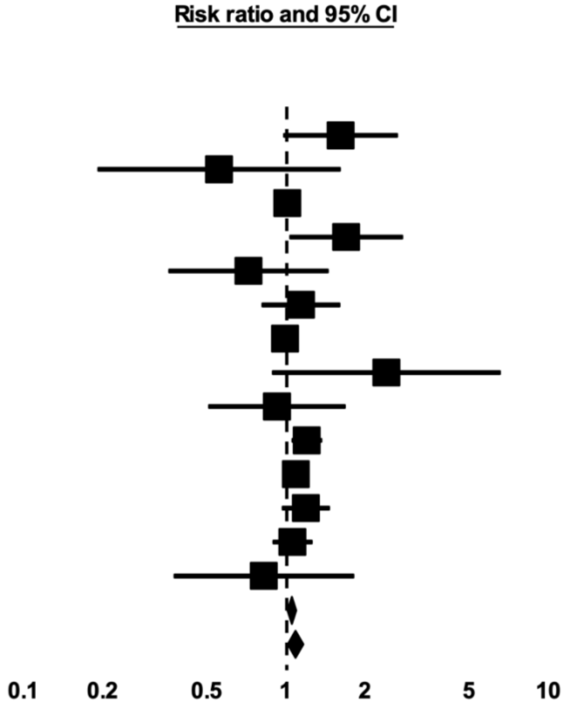

Supplemental Figure 1. The association between overweight during preconception and miscarriage. Heterogeneity: τ^2 = 0.01; Q-value = 21.52, df = 13 (*p* = 0.06); I^2 = 39.58%.

Model	Study name	Statistics for each study			
		Risk ratio	Lower limit	Upper limit	p-Value
	Zhang 2010	1.330	0.206	8.567	0.764
	Reyes 2012	0.900	0.351	2.307	0.826
	Pan 2016	1.130	1.017	1.256	0.023
	Zhou 2016	2.010	1.099	3.676	0.023
	Shen 2016	0.998	0.386	2.580	0.997
	MacKenna 2017	1.010	0.936	1.090	0.798
	Sheng 2017	0.469	0.027	8.109	0.602
	Pan 2018	1.150	0.492	2.687	0.747
	Zhang 2019	1.660	1.322	2.084	0.000
	Qu 2021	1.400	1.168	1.678	0.000
	Hu 2021	1.250	0.808	1.934	0.317
	Zheng 2022	1.453	1.066	1.981	0.018
Fixed	Pooled	1.126	1.066	1.190	0.000
Random	Pooled	1.271	1.101	1.467	0.001

Risk ratio and 95% CI

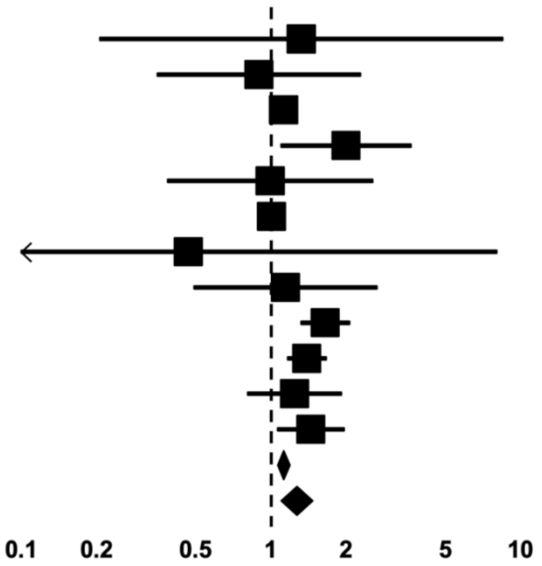

Supplemental Figure 2. The association between obesity during preconception and miscarriage. Heterogeneity: $\tau^2 = 0.03$; Q-value = 31.61, df = 11 ($p = 0.001$); $I^2 = 65.20\%$.

Model	Study name	Statistics for each study				Risk ratio and 95% CI
		Risk ratio	Lower limit	Upper limit	p-Value	
	Li 2010	0.802	0.367	1.750	0.579	
	Pan 2016	1.110	1.057	1.166	0.000	
	Zhou 2016	2.050	1.301	3.231	0.002	
	Cai 2017	1.530	1.130	2.071	0.006	
	MacKenna 2017	0.940	0.790	1.118	0.485	
	Sheng 2017	2.515	0.537	11.787	0.242	
	Pan 2018	0.185	0.012	2.985	0.234	
	Zhang 2019	1.030	0.854	1.242	0.757	
	Fang 2020	0.942	0.845	1.050	0.279	
	Qu 2021	0.940	0.807	1.095	0.428	
	Hu 2021	0.930	0.746	1.159	0.519	
	Zheng 2022	0.859	0.682	1.081	0.196	
	Gudipally 2023	0.940	0.590	1.499	0.795	
Fixed	Pooled	1.058	1.017	1.100	0.005	
Random	Pooled	1.028	0.931	1.136	0.580	

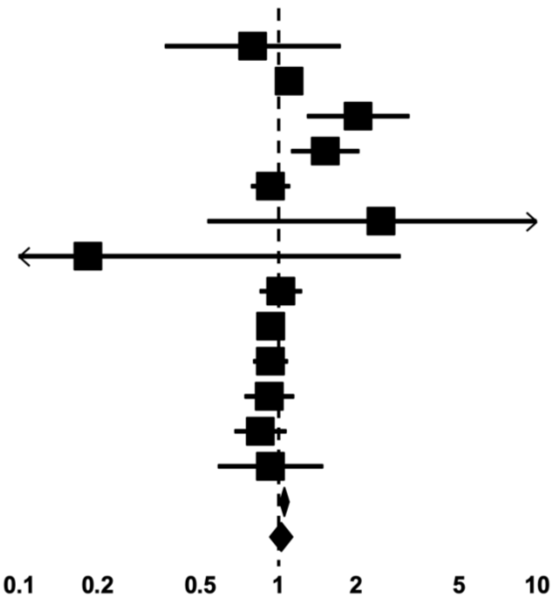

Supplemental Figure 3. The association between underweight during preconception and miscarriage. Heterogeneity: $\tau^2 = 0.02$; Q-value = 33.92, df = 12 (p = 0.001); I^2 = 64.62%.

Model	Study name	Statistics for each study			
		Risk ratio	Lower limit	Upper limit	p-Value
	Zhang 2010	1.173	0.452	3.044	0.744
	Qu 2021	1.110	0.992	1.242	0.069
	Yang 2021b	1.090	0.941	1.262	0.249
	Chen 2022	1.247	0.884	1.760	0.209
Fixed	Pooled	1.112	1.020	1.212	0.016
Random	Pooled	1.112	1.020	1.212	0.016

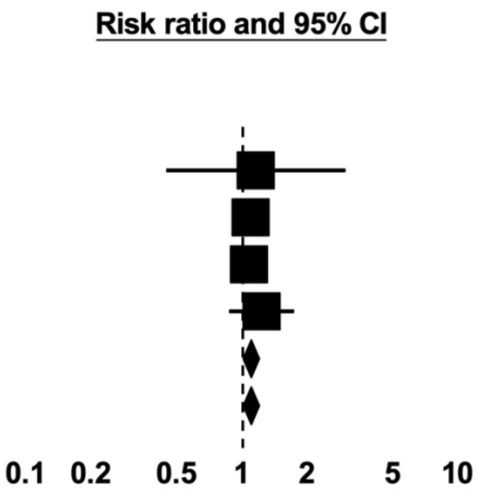

Supplemental Figure 4. The association between overweight during preconception and early pregnancy loss or early miscarriage. Heterogeneity: τ^2 = 0; Q-value = 0.51, df = 3 (*p* = 0.92); I^2 = 0%.

Model	Study name	Risk ratio	Lower limit	Upper limit	p-Value
	Zhang 2010	1.557	0.097	24.941	0.754
	Qu 2021	1.360	1.120	1.651	0.002
	Yang 2021b	1.470	1.136	1.903	0.003
	Chen 2022	1.105	0.173	7.058	0.916
Fixed	Pooled	1.397	1.197	1.630	0.000
Random	Pooled	1.397	1.197	1.630	0.000

(Header spanning Risk ratio through p-Value: Statistics for each study)

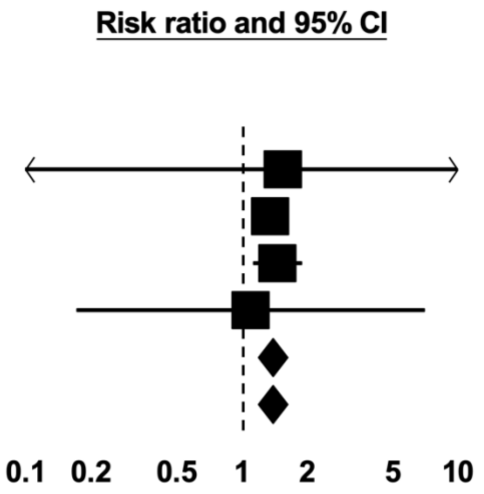

Supplemental Figure 5. The association between obesity during preconception and early pregnancy loss or early miscarriage.

Heterogeneity: $\tau^2 = 0$; Q-value = 0.29, df = 3 (p = 0.96); I^2 = 0%.

Model	Study name	Risk ratio	Lower limit	Upper limit	p-Value
	Li 2010	1.724	0.741	4.009	0.206
	Qu 2021	0.990	0.842	1.163	0.903
	Yang 2021b	0.820	0.611	1.101	0.187
	Chen 2022	0.914	0.565	1.476	0.712
Fixed	Pooled	0.960	0.839	1.097	0.546
Random	Pooled	0.958	0.830	1.105	0.554

(Statistics for each study)

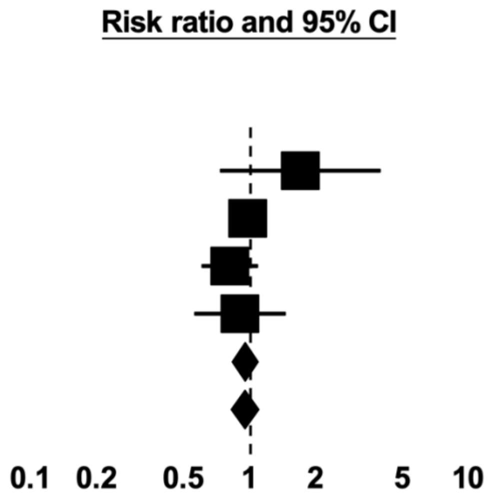

Supplemental Figure 6. The association between underweight during preconception and early pregnancy loss or early miscarriage. Heterogeneity: $\tau^2 = 0.001$; Q-value = 3.13, df = 3 ($p = 0.37$); $I^2 = 4.08\%$.

Model	Study name	Statistics for each study			
		Risk ratio	Lower limit	Upper limit	p-Value
	Hauger 2008	1.070	0.822	1.393	0.615
	Reyes 2012	1.763	0.298	10.430	0.532
	Pan 2016	1.130	0.951	1.342	0.164
	Yang 2021b	1.580	0.878	2.843	0.127
	Tang 2021	1.440	1.018	2.036	0.039
Fixed	Pooled	1.175	1.033	1.338	0.014
Random	Pooled	1.175	1.033	1.338	0.014

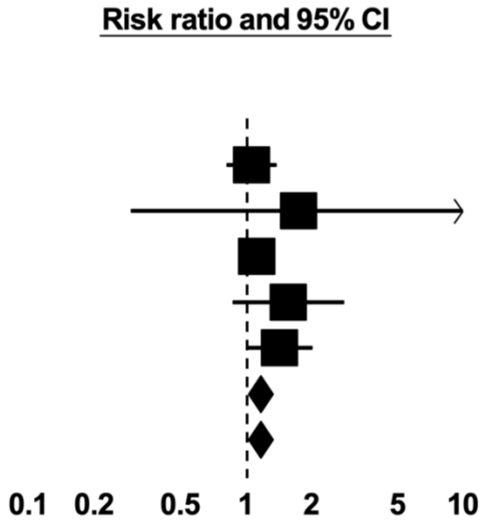

Supplemental Figure 7. The association between overweight during preconception and stillbirth. Heterogeneity: $\tau^2 = 0$; Q-value = 3.18, df = 4 ($p = 0.53$); $I^2 = 0\%$.

Model	Study name	Risk ratio	Lower limit	Upper limit	p-Value
		Statistics for each study			
	Hauger 2008	0.730	0.432	1.235	0.240
	Li 2010	2.463	0.225	26.997	0.461
	Pan 2016	1.050	0.890	1.239	0.564
	Chen 2021	1.000	0.781	1.281	1.000
	Tang 2021	0.730	0.534	0.998	0.048
Fixed	Pooled	0.964	0.853	1.090	0.559
Random	Pooled	0.932	0.785	1.107	0.425

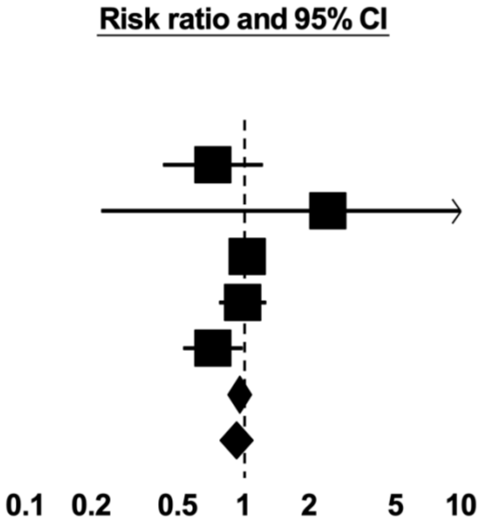

Supplemental Figure 8. The association between underweight during preconception and stillbirth. Heterogeneity: τ^2 = 0.01; Q-value = 5.81, df = 4 (*p* = 0.21); I^2 = 31.18%.

Model	Study name	Statistics for each study			
		Risk ratio	Lower limit	Upper limit	p-Value
	Patel 2018	1.080	0.958	1.217	0.208
	Zhou 2019a	1.660	0.788	3.499	0.183
	Chaurasia 2021	1.545	0.067	35.478	0.785
Fixed	Pooled	1.092	0.970	1.229	0.143
Random	Pooled	1.092	0.970	1.229	0.143

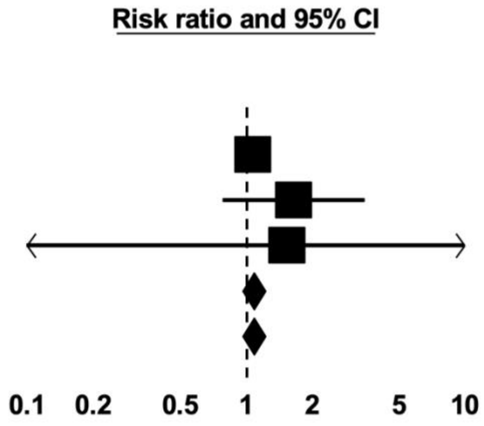

Supplemental Figure 9. The association between underweight during early pregnancy and stillbirth. Heterogeneity: τ^2 = 0; Q-value = 1.29, df = 2 (*p* = 0.52); I^2 = 0%.

Model	Study name	Risk ratio	Lower limit	Upper limit	p-Value
	Awan 2015	6.500	1.506	28.064	0.012
	Zhou 2019a	0.880	0.520	1.490	0.634
	Chaurasia 2021	1.429	0.092	22.079	0.798
Fixed	Pooled	1.116	0.685	1.816	0.660
Random	Pooled	1.888	0.440	8.102	0.392

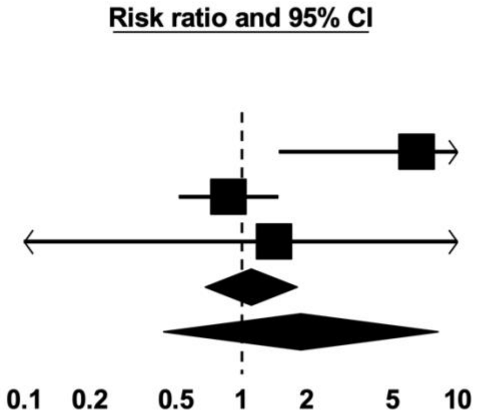

Supplemental Figure 10. The association between overweight during early pregnancy and stillbirth. Heterogeneity: τ^2 = 1.08; Q-value = 6.39, df = 2 (p = 0.04); I^2 = 68.70%.

Model	Study name	Risk ratio	Lower limit	Upper limit	p-Value
	Hauger 2008	1.070	0.737	1.554	0.722
	Zhou 2019a	1.520	0.649	3.562	0.335
	Chaurasia 2021	10.000	0.732	136.661	0.084
Fixed	Pooled	1.174	0.837	1.648	0.352
Random	Pooled	1.342	0.727	2.477	0.347

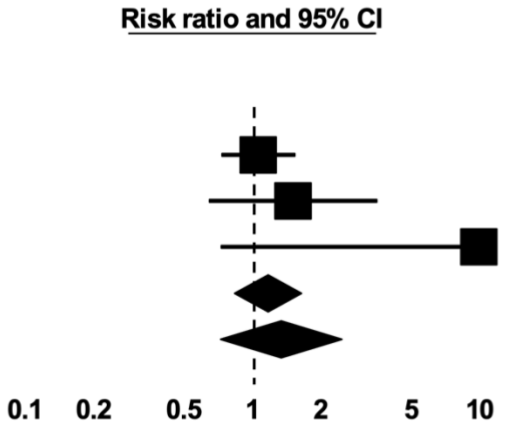

Supplemental Figure 11. The association between obesity during early pregnancy and stillbirth. Heterogeneity: $\tau^2 = 0.12$; Q-value = 3.17, df = 2 (*p* = 0.21); $I^2 = 36.89\%$.

Model	Study name	Statistics for each study			
		Risk ratio	Lower limit	Upper limit	p-Value
	Patel 2018	1.120	0.885	1.418	0.346
	Parks 2019	0.970	0.862	1.092	0.614
	Ambedkar 2021	1.185	0.376	3.734	0.772
	Shi 2022	0.590	0.571	0.610	0.000
Fixed	Pooled	0.619	0.600	0.639	0.000
Random	Pooled	0.879	0.587	1.317	0.533

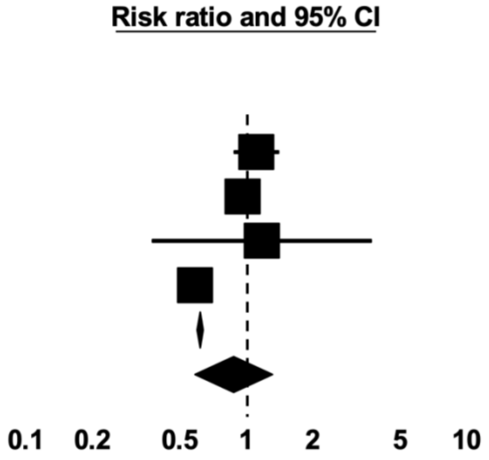

Supplemental Figure 12. The association between mild anemia during pregnancy and stillbirth. Heterogeneity: τ^2 = 0.13; Q-value = 88.70, df = 3 (p < 0.001); I^2 = 96.62%.

Model	Study name	Risk ratio	Lower limit	Upper limit	p-Value
		**Statistics for each study**			
	Parks 2019	1.090	0.981	1.211	0.109
	Ambedkar 2021	1.533	0.639	3.676	0.339
	Shi 2022	0.790	0.770	0.810	0.000
Fixed	Pooled	0.804	0.785	0.824	0.000
Random	Pooled	0.970	0.716	1.312	0.842

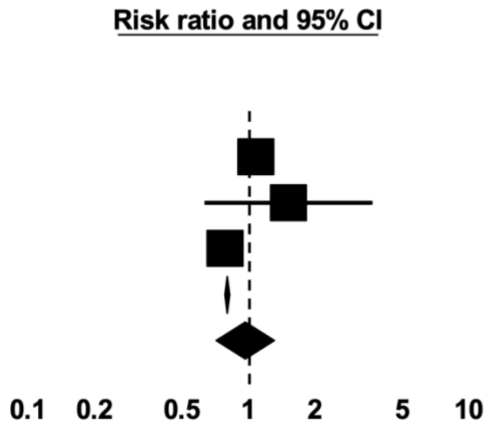

Supplemental Figure 13. The association between moderate anemia during pregnancy and stillbirth. Heterogeneity: τ^2 = 0.05; Q-value = 36.01, df = 2 (*p* < 0.001); I^2 = 94.45%.

Model	Study name	Statistics for each study				Risk ratio and 95% CI
		Risk ratio	Lower limit	Upper limit	p-Value	
	Abeysena 2010	1.300	0.387	4.371	0.672	
	Ambedkar 2021	26.848	1.628	442.900	0.021	
	Kalla 2022	0.940	0.642	1.375	0.750	
Fixed	Pooled	1.022	0.713	1.465	0.905	
Random	Pooled	1.630	0.511	5.201	0.409	

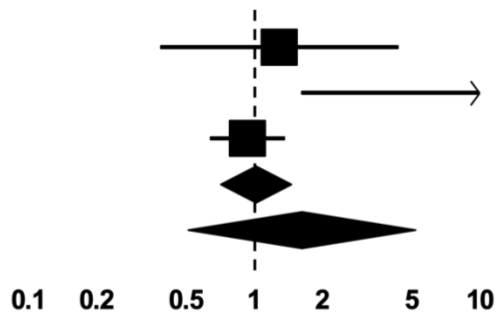

Supplemental Figure 14. The association between any anemia during pregnancy and miscarriage. Heterogeneity: τ^2 = 0.63; Q-value = 5.56, df = 2 (*p* = 0.06); I^2 = 64.03%.

Model	Study name	Statistics for each study			
		Odds ratio	Lower limit	Upper limit	p-Value
	Li 2010	0.804	0.603	1.070	0.135
	Zhang 2019	0.910	0.863	0.960	0.001
	Chen 2022	0.947	0.784	1.144	0.573
Fixed	Pooled	0.909	0.864	0.956	0.000
Random	Pooled	0.909	0.864	0.956	0.000

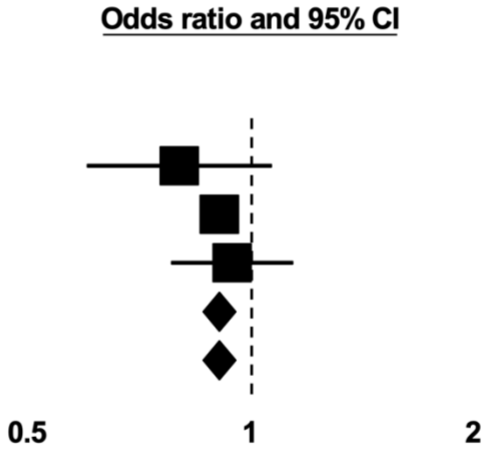

Supplemental Figure 15. The association between underweight during preconception and implantation rate. Heterogeneity: τ^2 = 0; Q-value = 0.89, df = 2 (p = 0.64); I^2 = 0%.

Model	Study name	Odds ratio	Lower limit	Upper limit	p-Value
	Shen 2016	0.857	0.633	1.162	0.322
	Zhang 2019	0.950	0.912	0.990	0.015
	Maged 2019	0.424	0.150	1.203	0.107
	Chen 2022	0.931	0.796	1.089	0.371
Fixed	Pooled	0.946	0.909	0.984	0.006
Random	Pooled	0.946	0.909	0.984	0.006

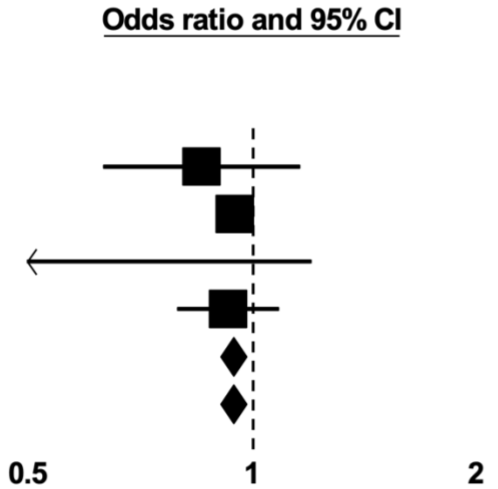

Supplemental Figure 16. The association between overweight during preconception and implantation rate. Heterogeneity: τ^2 = 0; Q-value = 2.75, df = 3 (*p* = 0.43); I^2 = 0%.

Model	Study name	Odds ratio	Lower limit	Upper limit	p-Value
	Shen 2016	0.602	0.370	0.978	0.040
	Sheng 2017	0.405	0.223	0.736	0.003
	Zhang 2019	0.800	0.733	0.873	0.000
	Maged 2019	0.212	0.071	0.634	0.005
	Chen 2022	0.837	0.392	1.786	0.646
Fixed	Pooled	0.777	0.714	0.845	0.000
Random	Pooled	0.589	0.403	0.862	0.006

Statistics for each study

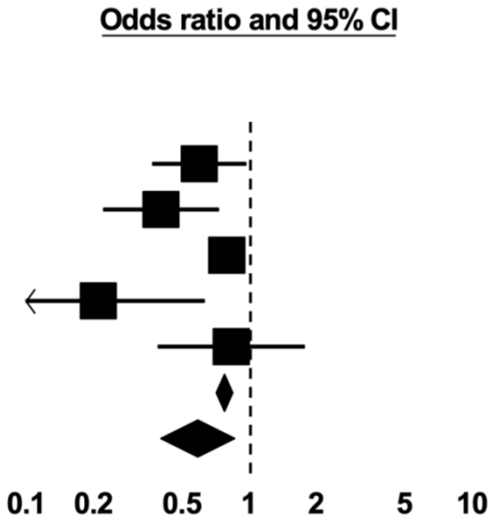

Supplemental Figure 17. The association between obesity during preconception and implantation rate. Heterogeneity: τ^2 = 0.11; Q-value =11.50, df = 4 (*p* = 0.02); I^2 = 65.23%.

Model	Study name	Odds ratio	Lower limit	Upper limit	p-Value
	Li 2010	0.827	0.581	1.177	0.292
	Sheng 2017	1.071	0.515	2.227	0.855
	Pan 2018	1.795	1.004	3.210	0.048
	Zhang 2019	0.900	0.823	0.984	0.021
Fixed	Pooled	0.911	0.837	0.992	0.032
Random	Pooled	0.985	0.767	1.265	0.906

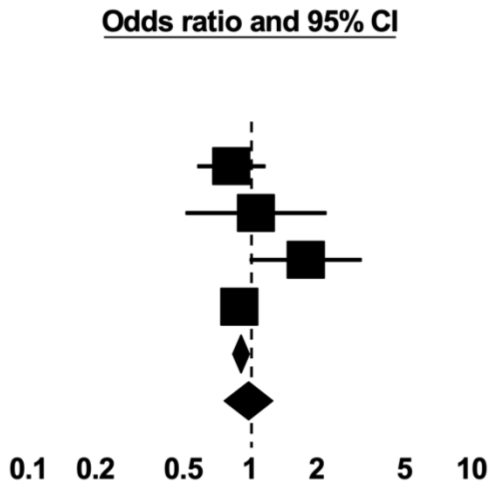

Supplemental Figure 18. The association between underweight during preconception and biochemical pregnancy rate. Heterogeneity: τ^2 = 0.03; Q-value = 5.78, df = 3 (p = 0.12); I^2 = 48.14%.

Model	Study name	Statistics for each study				Odds ratio and 95% CI
		Odds ratio	Lower limit	Upper limit	p-Value	
	Sheng 2017	1.285	0.820	2.012	0.274	
	Pan 2018	0.903	0.667	1.222	0.509	
	Zhang 2019	0.960	0.890	1.036	0.291	
	Maged 2019	0.490	0.212	1.131	0.095	
Fixed	Pooled	0.959	0.892	1.031	0.257	
Random	Pooled	0.956	0.808	1.130	0.595	

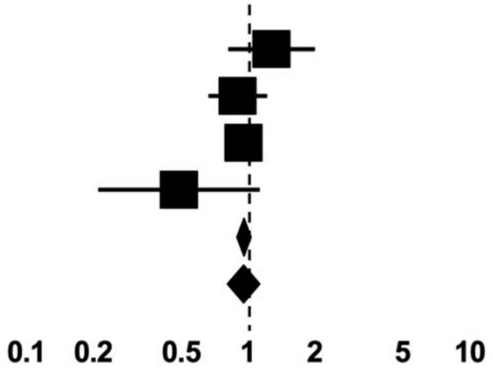

Supplemental Figure 19. The association between overweight during preconception and biochemical pregnancy rate. Heterogeneity: τ^2 = 0.01; Q-value = 4.26, df = 3 (p = 0.24); I^2 = 29.57%.

Model	Study name	Statistics for each study			
		Odds ratio	Lower limit	Upper limit	p-Value
	Sheng 2017	0.676	0.354	1.291	0.236
	Pan 2018	1.132	0.722	1.775	0.589
	Zhang 2019	0.830	0.736	0.935	0.002
	Maged 2019	0.237	0.102	0.547	0.001
Fixed	Pooled	0.822	0.735	0.920	0.001
Random	Pooled	0.712	0.463	1.094	0.121

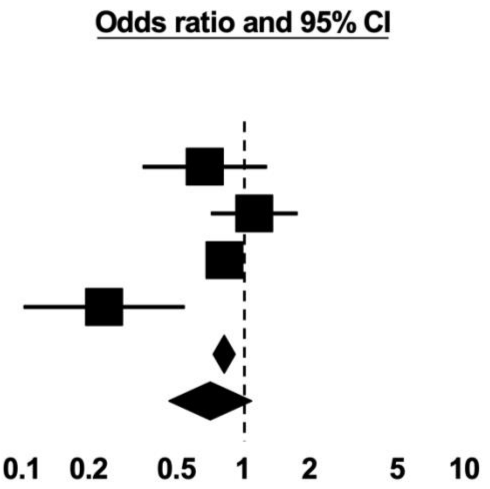

Supplemental Figure 20. The association between obesity during preconception and biochemical pregnancy rate. Heterogeneity: τ^2 = 0.13; Q-value = 10.79, df = 3 (*p* = 0.01); I^2 = 72.18%.

Model	Study name	Statistics for each study				Odds ratio and 95% CI
		Odds ratio	Lower limit	Upper limit	p-Value	
	Li 2010	0.756	0.526	1.088	0.132	
	Sharma 2014	0.647	0.133	3.135	0.588	
	Cai 2017	0.982	0.844	1.142	0.813	
	MacKenna 2017	1.030	0.905	1.173	0.656	
	Sheng 2017	0.856	0.431	1.702	0.658	
	Narasati 2017	1.159	0.788	1.705	0.454	
	Pan 2018	1.919	1.068	3.447	0.029	
	Zhang 2019	0.910	0.833	0.994	0.036	
	Zheng 2022	0.880	0.768	1.008	0.065	
	Chen 2022	0.910	0.691	1.198	0.503	
Fixed	Pooled	0.942	0.891	0.996	0.036	
Random	Pooled	0.950	0.879	1.025	0.187	

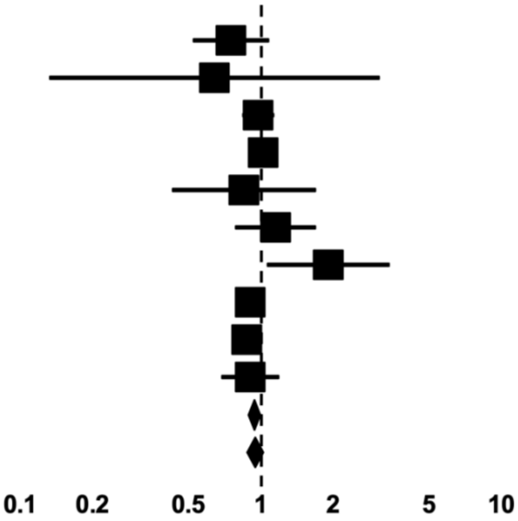

Supplemental Figure 21. The association between underweight during preconception and clinical pregnancy rate.

Heterogeneity: τ^2 = 0.003; Q-value = 12.19, df = 9 (*p* = 0.20); I^2 = 26.14%.

Model	Study name	Odds ratio	Lower limit	Upper limit	p-Value
	Zhang 2010	0.958	0.760	1.208	0.717
	Sharma 2014	1.161	0.763	1.767	0.485
	Shen 2016	0.803	0.521	1.238	0.321
	Cai 2017	0.872	0.737	1.031	0.109
	MacKenna 2017	1.000	0.960	1.042	1.000
	Sheng 2017	0.943	0.623	1.428	0.783
	Pan 2018	0.933	0.684	1.273	0.662
	Zhang 2019	0.960	0.902	1.022	0.201
	Maged 2019	0.388	0.122	1.230	0.108
	Zheng 2022	0.992	0.891	1.104	0.883
	Chen 2022	0.945	0.750	1.192	0.635
Fixed	Pooled	0.981	0.951	1.012	0.227
Random	Pooled	0.981	0.951	1.012	0.227

(Column group header: Statistics for each study)

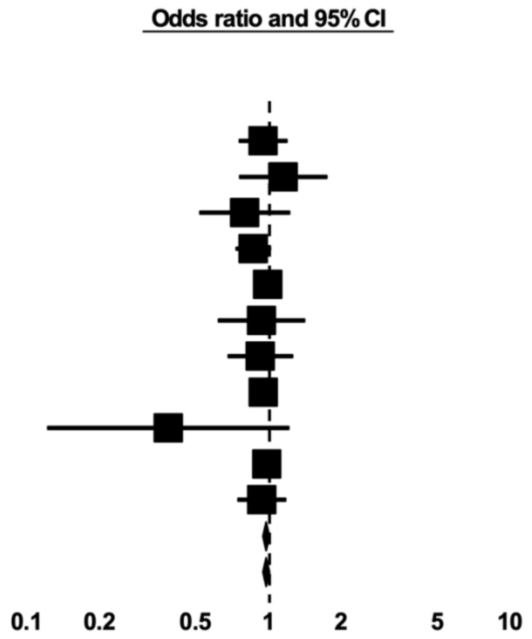

Supplemental Figure 22. The association between overweight during preconception and clinical pregnancy rate.

Heterogeneity: $\tau^2 = 0$; Q-value = 7.45, df = 10 (p = 0.68); I^2 = 0%.

Model	Study name	Odds ratio	Lower limit	Upper limit	p-Value
	Zhang 2010	0.973	0.435	2.177	0.948
	Sharma 2014	0.544	0.272	1.088	0.085
	Shen 2016	0.629	0.335	1.182	0.149
	MacKenna 2017	0.960	0.930	0.991	0.012
	Sheng 2017	0.383	0.198	0.740	0.004
	Pan 2018	1.197	0.756	1.893	0.443
	Zhang 2019	0.810	0.715	0.917	0.001
	Maged 2019	0.244	0.078	0.763	0.015
	Zheng 2022	1.125	0.908	1.393	0.281
	Chen 2022	0.683	0.242	1.927	0.471
Fixed	Pooled	0.949	0.921	0.978	0.001
Random	Pooled	0.852	0.725	1.003	0.054

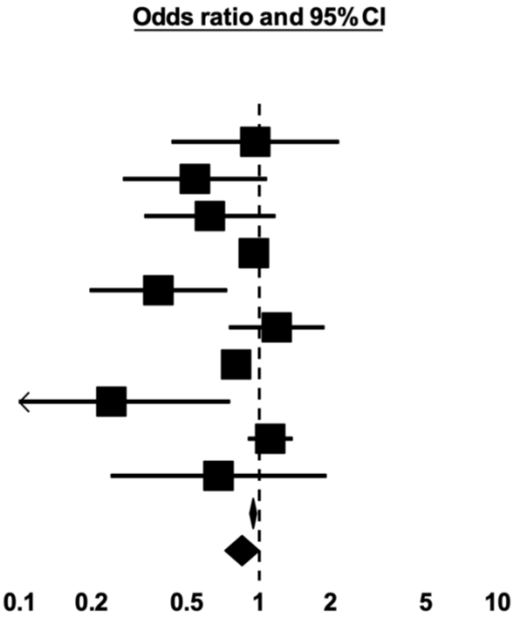

Supplemental Figure 23. The association between obesity during preconception and clinical pregnancy rate. Heterogeneity: τ^2 = 0.03; Q-value = 27.42, df = 9 (p = 0.001); I^2 = 67.17%.

Model	Study name	Statistics for each study			
		Odds ratio	Lower limit	Upper limit	p-Value
	Li 2010	0.841	0.572	1.238	0.381
	Sharma 2014	0.790	0.162	3.842	0.770
	Cai 2017	0.800	0.680	0.941	0.007
	MacKenna 2017	1.040	0.906	1.194	0.578
	Pan 2018	2.294	1.267	4.156	0.006
	Zhang 2019	0.910	0.833	0.994	0.036
	Zheng 2022	0.935	0.820	1.066	0.315
	Chen 2022	1.081	0.827	1.413	0.570
Fixed	Pooled	0.935	0.882	0.990	0.022
Random	Pooled	0.956	0.857	1.066	0.416

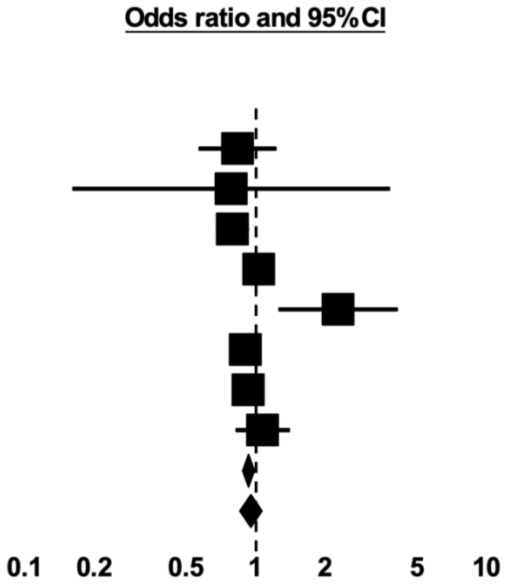

Supplemental Figure 24. The association between underweight during preconception and live birth rate. Heterogeneity: τ^2 = 0.01; Q-value = 16.42, df = 7 (p = 0.02); I^2 = 57.38%.

Model	Study name	Odds ratio	Lower limit	Upper limit	p-Value
	Zhang 2010	0.986	0.415	2.344	0.975
	Sharma 2014	1.115	0.597	2.082	0.732
	Shen 2016	0.679	0.355	1.299	0.242
	MacKenna 2017	0.960	0.930	0.990	0.010
	Pan 2018	1.155	0.707	1.886	0.565
	Zhang 2019	0.700	0.616	0.795	0.000
	Zheng 2022	0.948	0.765	1.174	0.625
	Chen 2022	0.943	0.334	2.661	0.912
Fixed	Pooled	0.944	0.916	0.972	0.000
Random	Pooled	0.889	0.751	1.053	0.174

(Statistics for each study)

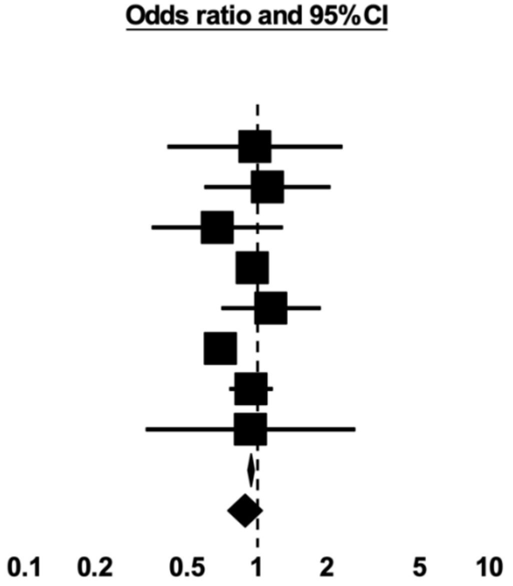

Supplemental Figure 25. The association between obesity during preconception and live birth rate. Heterogeneity: τ^2 = 0.025; Q-value = 24.19, df = 7 (*p* = 0.001); I^2 = 71.06%.

Model	Study name	Statistics for each study			
		Odds ratio	Lower limit	Upper limit	p-Value
	Zhang 2010	0.878	0.680	1.132	0.315
	Sharma 2014	1.127	0.725	1.751	0.595
	Shen 2016	0.932	0.604	1.438	0.750
	Cai 2017	0.920	0.770	1.100	0.359
	MacKenna 2017	1.000	0.951	1.051	1.000
	Pan 2018	0.925	0.662	1.291	0.646
	Zhang 2019	0.920	0.862	0.982	0.012
	Zheng 2022	0.971	0.871	1.083	0.596
	Chen 2022	0.903	0.724	1.125	0.363
Fixed	Pooled	0.964	0.931	0.999	0.043
Random	Pooled	0.964	0.931	0.999	0.043

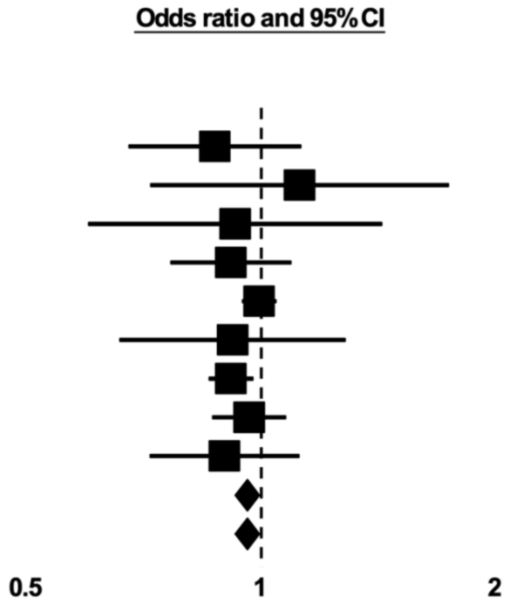

Supplemental Figure 26. The association between overweight during preconception and live birth rate. Heterogeneity: $\tau^2 = 0$; Q-value = 5.74, df = 8 (p = 0.68); I^2 = 0%.

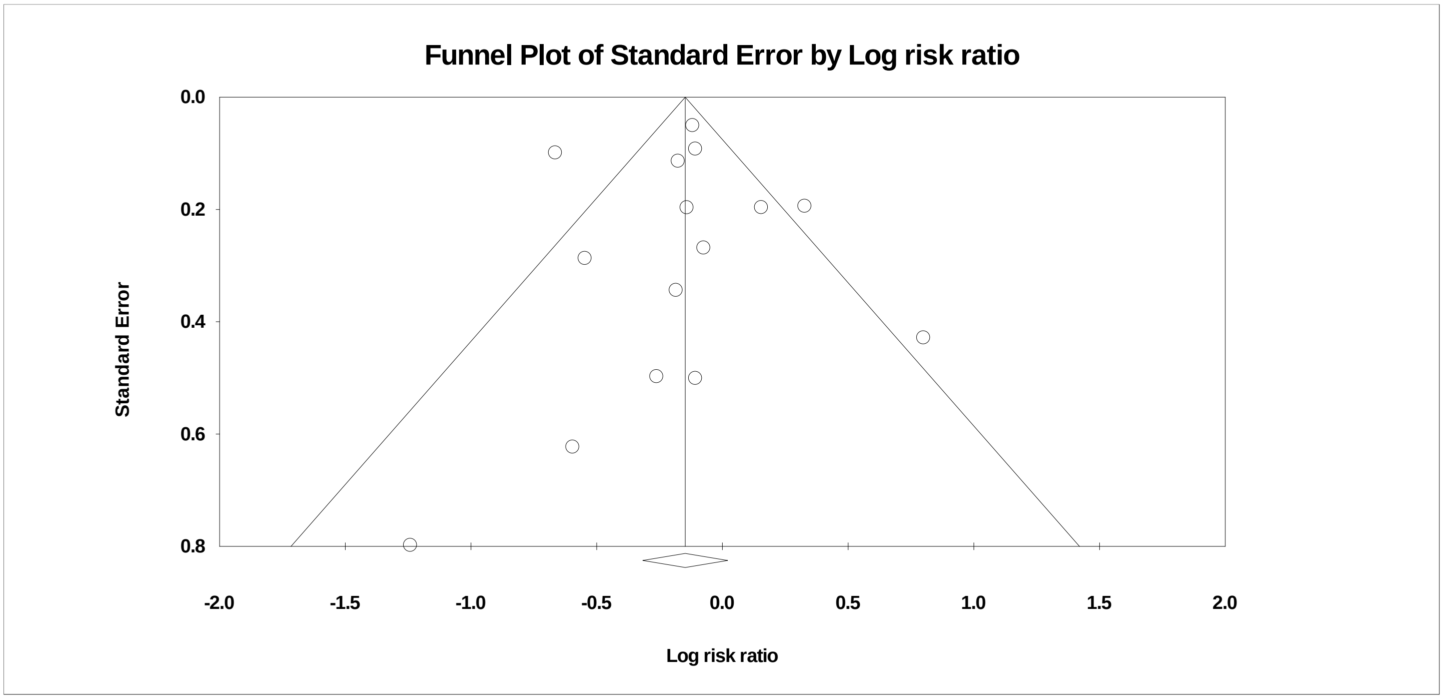

Supplemental Figure 27. Funnel plot for the effect of multiple micronutrient supplementation on stillbirth.

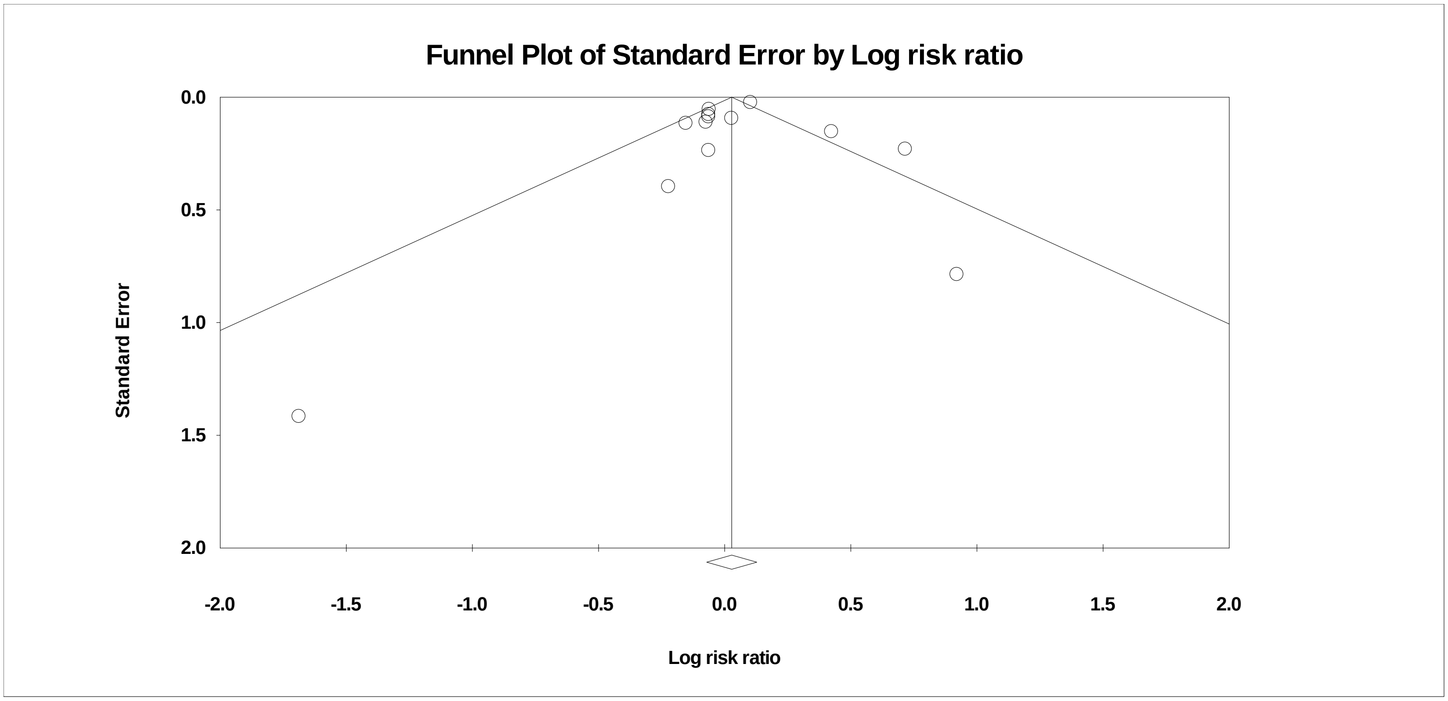

Supplemental Figure 28. Funnel plot for the association between preconceptional underweight and miscarriage.

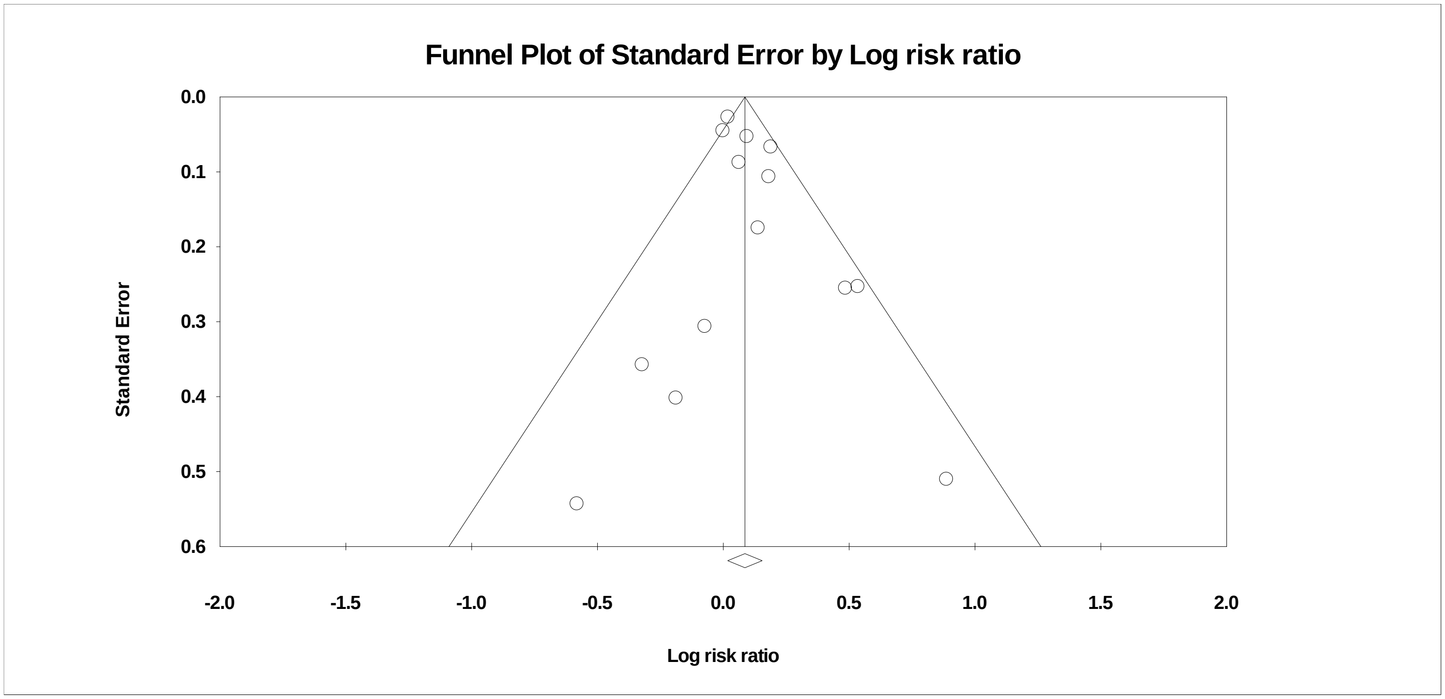

Supplemental Figure 29. Funnel plot for the association between preconceptional overweight and miscarriage.

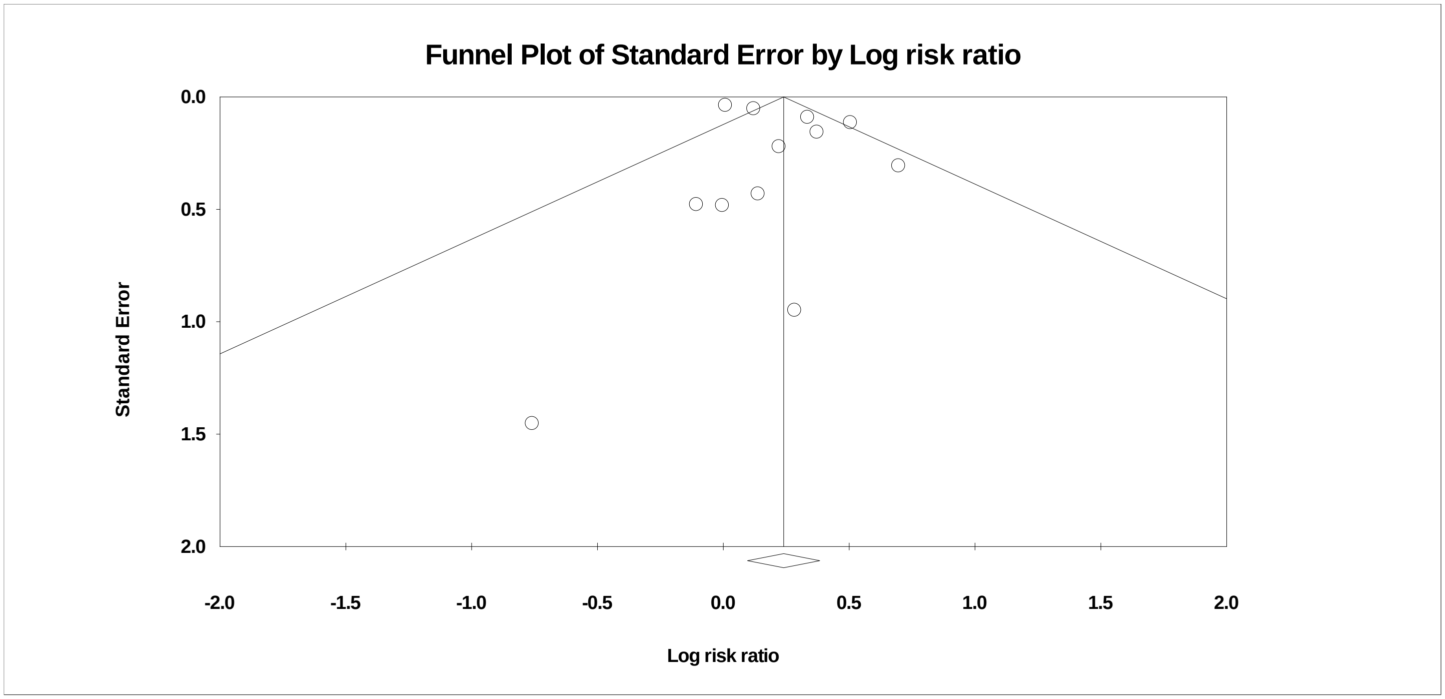

Supplemental Figure 30. Funnel plot for the association between preconceptional obesity and miscarriage.

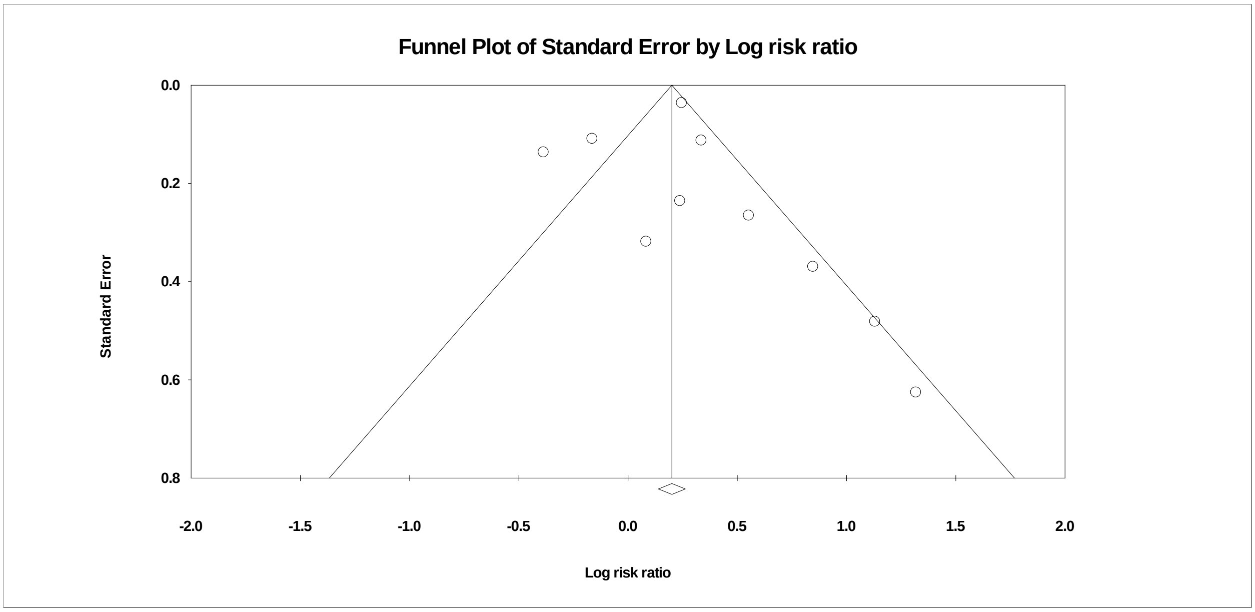

Supplemental Figure 31. Funnel plot for the association between any anemia during pregnancy and stillbirth.

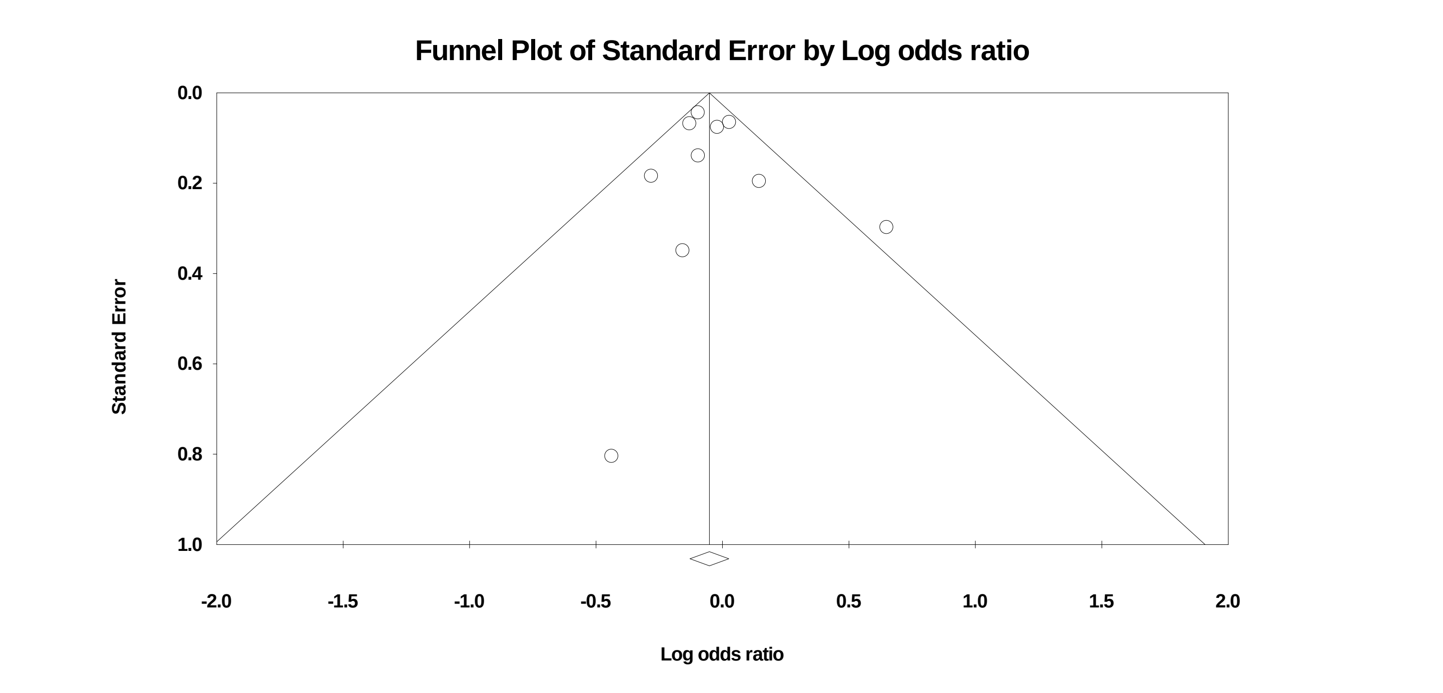

Supplemental Figure 32. Funnel plot for the association between preconceptional underweight and clinical pregnancy.

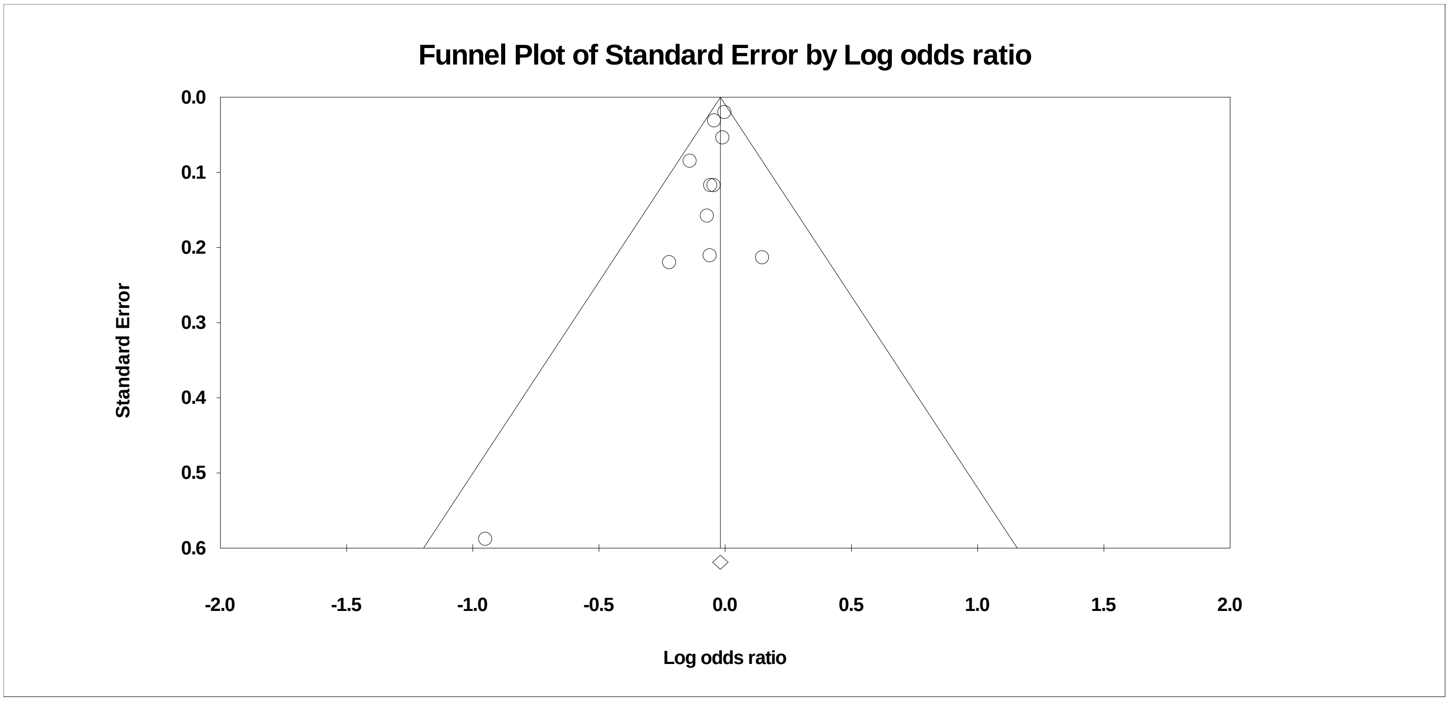

Supplemental Figure 33. Funnel plot for the association between preconceptional overweight and clinical pregnancy.

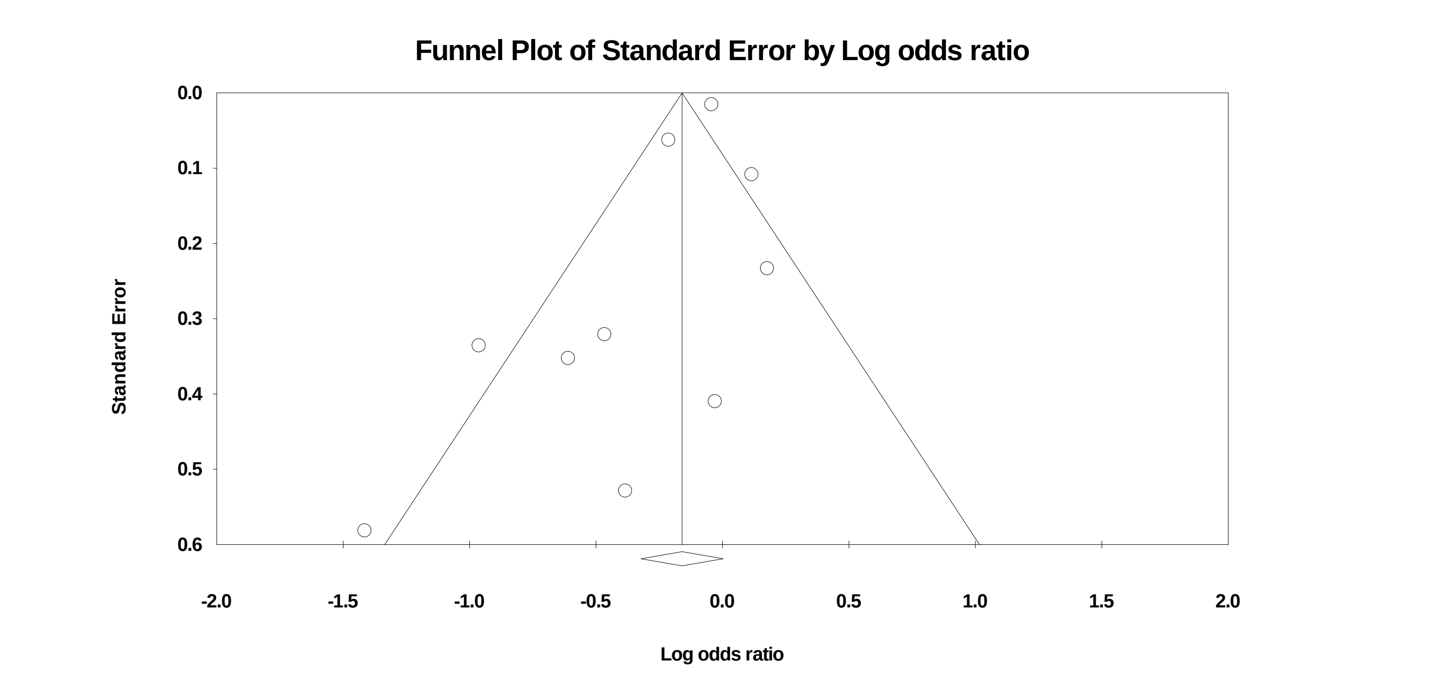

Supplemental Figure 34. Funnel plot for the association between preconceptional obesity and clinical pregnancy.

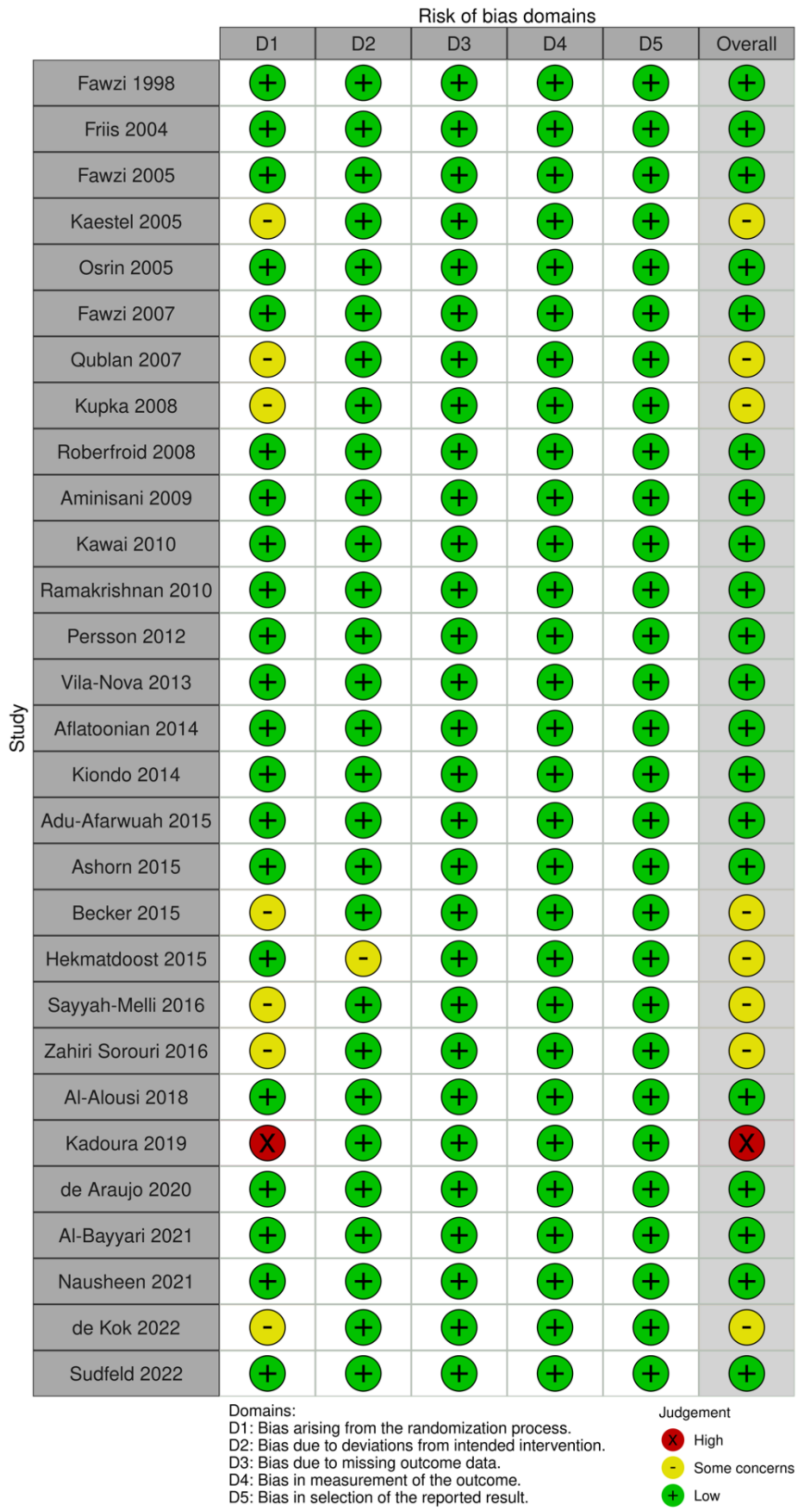

Supplemental Figure 35. Risk of bias assessment for the individually randomized controlled trials.

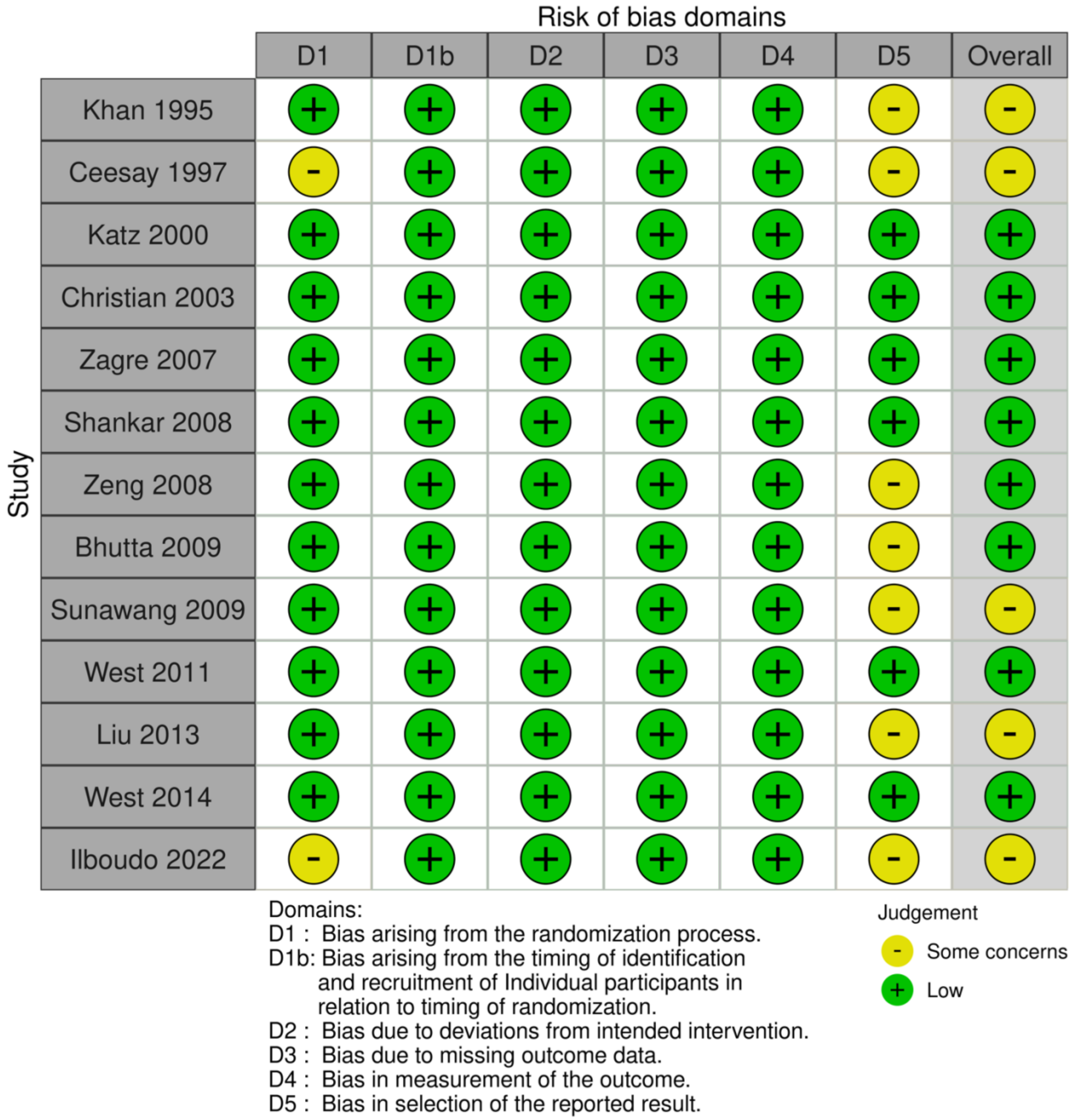

Supplemental Figure 36. Risk of bias assessment for the cluster randomized controlled trials.

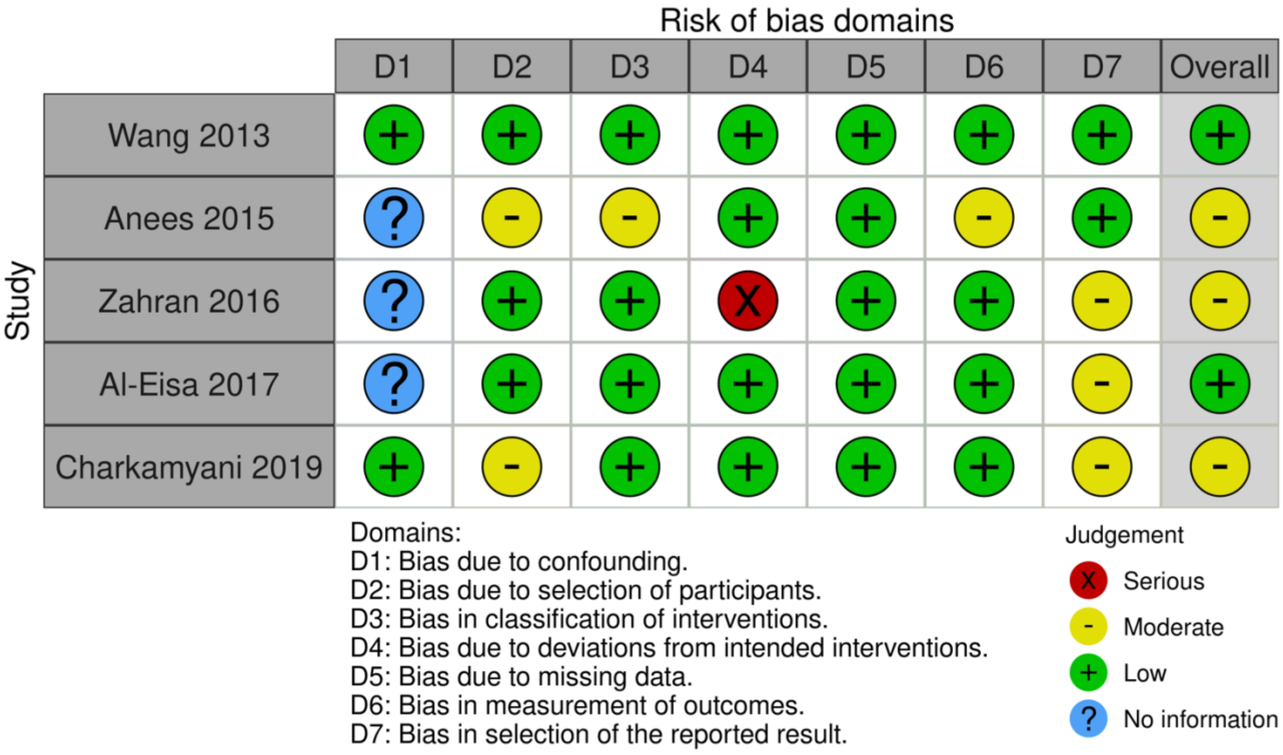

Supplemental Figure 37. Risk of bias assessment for the non-randomized intervention studies.

Supplemental Table 1. PubMed search strategy for the impact of nutrition on reproductive outcomes of women in low- and middle-income countries

No.	Concept	Search terms
1	Intervention Studies	"Clinical Trials as Topic"[Mesh] OR “Randomized Controlled Trial”[pt] OR Clinical Trial[pt] OR “Controlled Clinical Trial”[pt] OR "randomized controlled trials as topic"[MeSH] OR intervention*[tiab] OR "random allocation"[MeSH] OR random*[tiab] OR trial*[tiab] OR "Clinical Trial Protocols as Topic"[Mesh] OR "Clinical Trial Protocol"[pt] OR "Clinical Study"[pt] OR "Clinical Studies as Topic"[Mesh] OR "Therapeutic Uses"[Mesh] OR "therapeutic use"[Subheading]
2	Cohort Studies	“Cohort Studies”[MeSH] OR cohort*[tiab] or prospective[tiab] or longitudinal[tiab]
3	Micronutrients	"Micronutrients"[Mesh] OR micronutrient*[tiab] OR "Minerals"[Mesh] OR Mineral*[tiab] OR "Vitamins"[Mesh] OR vitamin*[tiab] OR "Dietary Supplements"[Mesh] OR "Calcium"[Mesh] OR calcium[tiab] OR "Magnesium"[Mesh] OR magnesium[tiab] OR "Phosphorus, Dietary"[Mesh] OR phosphorus[tiab] OR "Potassium, Dietary"[Mesh] OR potassium[tiab] OR "Boron"[Mesh] OR boron[tiab] OR "Cobalt"[Mesh] OR cobalt[tiab] OR "Chromium"[Mesh] OR chromium[tiab] OR "Copper"[Mesh] OR copper[tiab] OR "Iodine"[Mesh] OR iodine[tiab] OR "Iron"[Mesh] OR iron[tiab] OR "Ferritins"[Mesh] OR ferritin[tiab] OR "Transferrin"[Mesh] OR transferrin[tiab] OR "Hematocrit"[Mesh] OR hematocrit[tiab] OR haematocrit[tiab] OR "Manganese"[Mesh] OR manganese[tiab] OR "Molybdenum"[Mesh] OR molybdenum[tiab] OR "Selenium"[Mesh] OR selenium[tiab] OR "Zinc"[Mesh] OR zinc[tiab] OR “Thiamine”[Mesh] OR thiamin*[tiab] OR “Riboflavin”[Mesh] OR riboflavin[tiab] OR “Niacin”[Mesh] OR niacin[tiab] OR “Pantothenic Acid"[Mesh] OR “pantothenic acid”[tiab] OR "Pyridoxine"[Mesh] OR pyridox*[tiab] OR "Biotin"[Mesh] OR biotin[tiab] OR "Folic Acid"[Mesh] OR folate[tiab] OR “folic acid”[tiab] OR "Vitamin B 12"[Mesh] OR cobalamin*[tiab] OR "Methylmalonic Acid"[Mesh] OR “Methylmalonic acid*”[tiab] OR "Homocysteine"[Mesh] OR homocysteine[tiab] OR "Vitamin A"[Mesh] OR retinol[tiab] OR "Ascorbic Acid"[Mesh] OR “ascorbic acid”[tiab] OR calciferol*[tiab] OR "Ergocalciferols"[Mesh] OR ergocalciferol[tiab] OR "Vitamin D"[Mesh] OR cholecaliferol[tiab] OR "Tocopherols"[Mesh] OR tocopherol*[tiab] OR "Tocotrienols"[Mesh] OR tocotrienol*[tiab] OR "Vitamin K"[Mesh] OR phylloquinone[tiab] OR menaquinone[tiab] OR "Choline"[Mesh] OR Choline[tiab] OR "Carotenoids"[Mesh] OR carotenoid*[tiab] OR carotene[tiab] OR "Hemoglobins"[Mesh] OR hemoglobin*[tiab] OR haemoglobin*[tiab] OR "Anemia"[Mesh] OR anemia[tiab] OR anaemia[tiab] OR anemic[tiab] OR anaemic[tiab]
4	Macronutrients	macronutrient*[tiab] OR "Carbohydrates"[Mesh] OR carbohydrate*[tiab] OR "Glucose"[Mesh] OR glucose[tiab] OR "Dietary Fiber"[Mesh] OR fiber*[tiab] OR fat[tiab] OR "Fatty Acids"[Mesh] OR “fatty acid*”[tiab] OR “Omega-3”[tiab] OR “Omega-6”[tiab] OR "Lipids"[Mesh] OR lipid*[tiab] OR "Triglycerides"[Mesh] OR triglyceride*[tiab] OR "Cholesterol"[Mesh] OR cholesterol*[tiab] OR "Proteins"[Mesh] OR protein*[tiab] OR "Amino Acids"[Mesh] OR “amino acid*”[tiab]
5	Dietary intake (generic terms)	"Nutrients"[Mesh] OR nutrient*[tiab] OR "Diet"[Mesh] OR diet[tiab] OR dietary[tiab] OR "Food"[Mesh] OR food*[tiab] OR "Eating"[Mesh] OR eating[tiab] OR "Energy Intake"[Mesh] OR caloric[tiab] OR calorie[tiab]
6	Anthropometry	"Body Weight"[Mesh] OR Weight[tiab] OR "Body Mass Index"[Mesh] OR "body mass index"[tiab] OR BMI[tiab] OR "Obesity"[Mesh] OR obesity[tiab] OR obese[tiab] OR "Thinness"[Mesh] OR underweight[tiab] OR "Malnutrition"[Mesh] OR malnutrition[tiab] OR malnourished[tiab] OR undernutrition[tiab] OR undernourished[tiab] OR "Weight Gain"[Mesh] OR "Gestational Weight Gain"[Mesh] OR "Body Weight Changes"[Mesh] OR "Body-Weight Trajectory"[Mesh] OR "Overweight"[Mesh] OR overweight[tiab] OR "Anthropometry"[Mesh] OR anthropometry[tiab] OR anthropometric[tiab] OR "Body Height"[Mesh] OR height[tiab] OR stature[tiab] OR stunting[tiab] OR stunted[tiab] OR "Overnutrition"[Mesh] OR overnutrition[tiab] OR “over-nutrition”[tiab] OR "Nutritional Status"[Mesh] OR nutrition[tiab] OR “BMI-for-age”[tiab] OR “height-for-age”[tiab] OR “weight-for-age”[tiab]
7	Fertility, fecundity, menarche, menstruation	"Fertility"[Mesh] OR fertility[tiab] OR fecundity[tiab] OR infecundity[tiab] OR fecundability[tiab] OR “number of pregnancies”[tiab] OR “number of children”[tiab] OR “number of offspring”[tiab] OR "Fertilization"[Mesh] OR fertilization[tiab] OR fertilisation[tiab] OR "Infertility"[Mesh] OR infertility[tiab] OR sterility[tiab] OR conception[tiab] OR "Ovulation"[Mesh] OR ovulation[tiab] OR imbedding[tiab] OR "Embryo Implantation"[Mesh] OR implantation[tiab] OR "Menarche"[Mesh] OR menarche[tiab] OR "Menstruation"[Mesh] OR menstrua*[tiab] OR amenorrh*[tiab] oligomenorrh*[tiab] OR "polymenorrh*"[tiab] OR "menorrhagia"[tiab]
8	Miscarriage	"Abortion, Spontaneous"[Mesh] OR miscarriage*[tiab] OR “spontaneous abortion*”[tiab] OR “pregnancy loss”[tiab]
9	Stillbirth	"Stillbirth"[Mesh] OR stillbirth*[tiab] OR "Fetal Death"[Mesh] OR “fetal death*”[tiab] OR “fetal loss”[tiab]
10	Adolescent girls and women	"Women"[Mesh] OR women[tiab] OR woman[tiab] OR "Adolescent"[Mesh] OR Adolescent*[tiab] OR teen[tiab] OR teens[tiab] OR teenager*[tiab] OR girl*[tiab] OR "Mothers"[Mesh] OR mother*[tiab]
11	Low- and middle-income countries	"Developing Countries"[MeSH] OR “developing countr*”[tiab] OR “developing nation*”[tiab] OR “less developed countr*”[tiab] OR “less developed nation*”[tiab] OR “third world nation*”[tiab] OR “third world countr*”[tiab] OR “under developed nation*”[tiab] OR “underdeveloped nation*”[tiab] OR “under developed countr*”[tiab] OR “underdeveloped countr*”[tiab] OR “middle income countr*”[tiab] OR “middle-income countr*”[tiab] OR “middle income nation*”[tiab] OR “middle-income nation*”[tiab] OR “low income countr*”[tiab] OR “low-income countr*”[tiab] OR “low income nation*”[tiab] OR “low-income nation*”[tiab] OR “poor countr*”[tiab] OR “poor nation*”[tiab] OR LMIC[tiab] OR LMICs[tiab] OR "Africa"[MeSH] OR "Asia"[MeSH] OR "South America"[MeSH] OR "Latin America"[MeSH] OR "Central America"[MeSH] OR africa[tiab] OR asia[tiab] OR “south america*”[tiab] OR “latin america*”[tiab] OR “central america*”[tiab] OR Afghanistan*[tiab] OR Albania*[tiab] OR Algeria*[tiab] OR Samoa*[tiab] OR Angola*[tiab] OR Armenia*[tiab] OR Azerbaijan*[tiab] OR Bangladesh*[tiab] OR Bengali[tiab] OR Belarus*[tiab] OR Belize[tiab] OR Benin[tiab] OR Bhutan*[tiab] OR Bolivia*[tiab] OR Bosnia*[tiab] OR Herzegovina*[tiab] OR Botswana*[tiab] OR Brazil*[tiab] OR Bulgaria*[tiab] OR “Burkina Faso”[tiab] OR Burkinabe[tiab] OR Burundi*[tiab] OR “Cabo Verd*”[tiab] OR “Cape Verd*”[tiab] OR Cambodia*[tiab] OR Cameroon*[tiab] OR “Central African*”[tiab] OR Chad*[tiab] OR China[tiab] OR Chinese[tiab] OR Colombia*[tiab] OR Comoros[tiab] OR Congo[tiab] OR “Costa Rica*”[tiab] OR “Cote d'Ivoire”[tiab] OR “Ivory Coast”[tiab] OR Cuba[tiab] OR Cuban[tiab] OR Djibouti[tiab] OR Dominica*[tiab] OR Ecuador[tiab] OR Egypt*[tiab] OR “El Salvador*”[tiab] OR Eritrea*[tiab] OR Ethiopia*[tiab] OR Fiji*[tiab] OR Gabon*[tiab] OR Gambia*[tiab] OR Georgia*[tiab] OR Ghana*[tiab] OR Grenada*[tiab] OR Guatemala*[tiab] OR Guinea*[tiab] OR Guyan*[tiab] OR Haiti*[tiab] OR Hondura*[tiab] OR India[tiab] OR Indian*[tiab] OR Indonesia*[tiab] OR Iran*[tiab] OR Iraq*[tiab] OR Jamaica*[tiab] OR Jordan*[tiab] OR Kazakh*[tiab] OR Kenya*[tiab] OR Kiribati[tiab] OR “People's Republic of Korea”[tiab] OR “North Korea”[tiab] OR Kosovo[tiab] OR Kosovar*[tiab] OR Kyrgyz*[tiab] OR Lao[tiab] OR Laos[tiab] OR Laotian*[tiab] OR Lebanon[tiab] OR Lebanes*[tiab] OR Lesotho[tiab] OR Liberia*[tiab] OR Libya*[tiab] OR Macedonia*[tiab] OR Madagascar*[tiab] OR Malawi*[tiab] OR Malaysia*[tiab] OR Maldives[tiab] OR Mali[tiab] OR “Marshall Island*”[tiab] OR “Mexico”[MeSH] OR Mexico[tiab] OR Mexican*[tiab] OR Micronesia*[tiab] OR Moldova*[tiab] OR Mongolia*[tiab] OR Montenegr*[tiab] OR Morocc*[tiab] OR Mozambique[tiab] OR Myanmar[tiab] OR Burmese*[tiab] OR Burma[tiab] OR Namibia*[tiab] OR Nepal*[tiab] OR Nicaragua*[tiab] OR Niger*[tiab] OR Pakistan*[tiab] OR Paraguay*[tiab] OR Peru*[tiab] OR Philippin*[tiab] OR Rwanda*[tiab] OR “Sao Tome”[tiab] OR Principe[tiab] OR Senegal*[tiab] OR Serbia*[tiab] OR “Sierra Leone*”[tiab] OR “Solomon Island*”[tiab] OR Somalia*[tiab] OR “South Africa*”[tiab] OR “Sri Lanka”[tiab] OR “St Lucia”[tiab] OR “Saint Lucia”[tiab] OR “St Vincent”[tiab] OR “Saint Vincent”[tiab] OR Grenad*[tiab] OR Sudan*[tiab] OR Suriname*[tiab] OR Swaziland*[tiab] OR Eswatini*[tiab] OR Syria*[tiab] OR Tajik*[tiab] OR Tanzania*[tiab] OR Zanzibar[tiab] OR Thai*[tiab] OR Timor*[tiab] OR Togo*[tiab] OR Tonga*[tiab] OR Tunisia*[tiab] OR Turkey[tiab] OR Turkish[tiab] OR Turkmen*[tiab] OR Tuvalu*[tiab] OR Uganda*[tiab] OR Ukrain*[tiab] OR Uzbeki*[tiab]

		OR Vanuatu*[tiab] OR Venezuela*[tiab] OR Vietnam*[tiab] OR "Viet nam*"[tiab] OR "West Bank"[tiab] OR Gaza*[tiab] OR Palestin*[tiab] OR Yemen*[tiab] OR Zambia*[tiab] OR Zimbabw*[tiab] OR "Western Sahara"[tiab] OR Argentin*[tiab] OR Russia*[tiab] OR Maurit*[tiab] OR Palau[tiab]
12	Animal studies	"Animals"[Mesh] NOT ("Animals"[Mesh] AND "Humans"[Mesh])
Total	(1 OR 2) AND (3 OR 4 OR 5 OR 6) AND (7 OR 8 OR 9) AND 10 AND 11 NOT 12	

Supplemental Table 2. Embase search strategy for the impact of nutrition on reproductive outcomes of women in low- and middle-income countries

No.	Concept	Search terms
1	Intervention Studies	'clinical trial'/exp OR 'randomized controlled trial'/exp OR 'clinical trial (topic)'/exp OR 'randomized controlled trial (topic)'/exp OR 'intervention'/exp OR intervention*:ab,ti OR 'randomization'/exp OR random*:ab,ti OR 'trial'/exp OR trial*:ab,ti OR 'clinical trial protocol'/exp OR 'clinical study'/exp
2	Cohort Studies	'cohort analysis'/exp OR 'prospective study'/exp OR cohort*:ab,ti OR prospective:ab,ti OR 'longitudinal study'/exp OR longitudinal:ab,ti
3	Micronutrients	'trace element'/exp OR 'micronutrient intake'/exp OR micronutrient*:ab,ti OR 'mineral'/exp OR 'minerals plus multivitamins'/exp OR 'mineral deficiency'/exp OR 'mineral supplementation'/exp OR Mineral*:ab,ti OR 'vitamin'/exp OR vitamin*:ab,ti OR 'vitamin deficiency'/exp OR 'dietary supplement'/exp OR 'vitamin supplementation'/exp OR 'calcium'/exp OR calcium:ab,ti OR 'magnesium'/exp OR magnesium:ab,ti OR 'phosphorus'/exp OR phosphorus:ab,ti OR 'potassium'/exp OR potassium:ab,ti OR 'boron'/exp OR boron:ab,ti OR 'cobalt'/exp OR cobalt:ab,ti OR 'chromium'/exp OR chromium:ab,ti OR 'copper'/exp OR copper:ab,ti OR 'iodine'/exp OR iodine:ab,ti OR 'iron'/exp OR iron:ab,ti OR 'ferritin'/exp OR ferritin:ab,ti OR 'transferrin'/exp OR transferrin:ab,ti OR 'hematocrit'/exp OR hematocrit:ab,ti OR haematocrit:ab,ti OR 'manganese'/exp OR manganese:ab,ti OR 'molybdenum'/exp OR molybdenum:ab,ti OR 'selenium'/exp OR selenium:ab,ti OR 'zinc'/exp OR zinc:ab,ti OR 'thiamine'/exp OR thiamin*:ab,ti OR 'riboflavin'/exp OR riboflavin:ab,ti OR 'nicotinic acid'/exp OR niacin:ab,ti OR 'pantothenic acid'/exp OR 'pantothenic acid':ab,ti OR 'pyridoxine'/exp OR pyridox*:ab,ti OR 'biotin'/exp OR biotin:ab,ti OR 'folic acid'/exp OR folate:ab,ti OR 'folic acid':ab,ti OR 'cyanocobalamin'/exp OR cobalamin*:ab,ti OR 'methylmalonic acid'/exp OR 'Methylmalonic acid*':ab,ti OR 'homocysteine'/exp OR homocysteine:ab,ti OR 'retinol'/exp OR retinol:ab,ti OR 'ascorbic acid'/exp OR 'ascorbic acid':ab,ti OR 'ergocalciferol'/exp OR calciferol*:ab,ti OR ergocalciferol:ab,ti OR 'vitamin D'/exp OR cholecaliferol:ab,ti OR 'tocopherol'/exp OR tocopherol*:ab,ti OR 'alpha tocotrienol'/exp OR tocotrienol*:ab,ti OR 'vitamin K group'/exp OR phylloquinone:ab,ti OR menaquinone:ab,ti OR 'choline'/exp OR Choline:ab,ti OR 'carotenoid'/exp OR carotenoid*:ab,ti OR carotene:ab,ti OR 'hemoglobin'/exp OR hemoglobin*:ab,ti OR haemoglobin*:ab,ti OR 'anemia'/exp OR anemia:ab,ti OR anaemia:ab,ti OR anemic:ab,ti OR anaemic:ab,ti
4	Macronutrients	'macronutrient'/exp OR 'macronutrient intake'/exp OR macronutrient*:ab,ti OR 'carbohydrate'/exp OR carbohydrate*:ab,ti OR 'glucose'/exp OR glucose:ab,ti OR 'dietary fiber'/exp OR 'dietary fiber*':ab,ti OR 'fat'/exp OR fat:ab,ti OR 'fatty acid'/exp OR 'fatty acid*':ab,ti OR 'Omega-3':ab,ti OR 'Omega-6':ab,ti OR 'lipid'/exp OR lipid*:ab,ti OR 'triacylglycerol'/exp OR triglyceride*:ab,ti OR 'cholesterol'/exp OR cholesterol*:ab,ti OR 'protein'/exp OR protein*:ab,ti OR 'amino acid'/exp OR 'amino acid*':ab,ti
5	Dietary intake (generic terms)	'nutrient'/exp OR nutrient*:ab,ti OR 'diet'/exp OR diet:ab,ti OR 'dietary intake'/exp OR dietary:ab,ti OR 'food'/exp OR 'food intake'/exp OR food*:ab,ti OR 'eating'/exp OR eating:ab,ti OR 'caloric intake'/exp OR caloric:ab,ti OR calorie:ab,ti
6	Anthropometry	'body weight'/exp OR Weight:ab,ti OR 'body mass'/exp OR 'body mass index':ab,ti OR BMI:ab,ti OR 'obesity'/exp OR obesity:ab,ti OR obese:ab,ti OR 'underweight'/exp OR underweight:ab,ti OR 'malnutrition'/exp OR malnutrition:ab,ti OR malnourished:ab,ti OR undernutrition:ab,ti OR undernourished:ab,ti OR 'body weight gain'/exp OR 'gestational weight gain'/exp OR 'body weight change'/exp OR 'weight trajectory (body weight)'/exp OR overweight:ab,ti OR 'anthropometry'/exp OR anthropometry:ab,ti OR anthropometric:ab,ti OR 'body height'/exp OR height:ab,ti OR stature:ab,ti OR 'stunting'/exp OR stunting:ab,ti OR stunted:ab,ti OR 'overnutrition'/exp OR overnutrition:ab,ti OR 'over-nutrition':ab,ti OR 'nutritional status'/exp OR 'nutrition'/exp OR nutrition:ab,ti OR 'BMI-for-age':ab,ti OR 'height-for-age':ab,ti OR 'weight-for-age':ab,ti
7	Fertility, fecundity, menarche, menstruation	'female fertility'/exp OR fertility:ab,ti OR fecundity:ab,ti OR infecundity:ab,ti OR fecundability:ab,ti OR 'number of pregnancies':ab,ti OR 'number of children':ab,ti OR 'number of offspring':ab,ti OR 'fertilization'/exp OR fertilization:ab,ti OR fertilisation:ab,ti OR 'female infertility'/exp OR infertility:ab,ti OR sterility:ab,ti OR 'conception'/exp OR conception:ab,ti OR 'ovulation'/exp OR ovulation:ab,ti OR imbedding:ab,ti OR 'embryo implantation':ab,ti OR 'menarche'/exp OR menarche:ab,ti OR 'menstruation'/exp OR menstrua*:ab,ti OR 'amenorrhea and oligomenorrhea'/exp OR amenorrh*:ab,ti oligomenorrh*:ab,ti OR 'polymenorrh*':ab,ti OR 'menorrhagia':ab,ti OR 'menorrhagia and metrorrhagia'/exp
8	Miscarriage	'spontaneous abortion'/exp OR miscarriage*:ab,ti OR 'spontaneous abortion*':ab,ti OR 'pregnancy loss':ab,ti
9	Stillbirth	'stillbirth'/exp OR stillbirth*:ab,ti OR 'fetal death*':ab,ti OR 'fetal loss':ab,ti
10	Adolescent girls and women	'female'/exp OR women:ab,ti OR woman:ab,ti OR 'adolescent'/exp OR adolescent*:ab,ti OR teen:ab,ti OR teens:ab,ti OR teenager*:ab,ti OR girl*:ab,ti OR 'mother'/exp OR mother*:ab,ti
11	Low- and middle-income countries	'developing country'/exp OR 'developing countr*':ab,ti OR 'developing nation*':ab,ti OR 'less developed countr*':ab,ti OR 'less developed nation*':ab,ti OR 'third world nation*':ab,ti OR 'third world countr*':ab,ti OR 'under developed nation*':ab,ti OR 'underdeveloped nation*':ab,ti OR 'under developed countr*':ab,ti OR 'underdeveloped countr*':ab,ti OR 'middle income countr*':ab,ti OR 'middle-income countr*':ab,ti OR 'middle income nation*':ab,ti OR 'middle-income nation*':ab,ti OR 'low income countr*':ab,ti OR 'low-income countr*':ab,ti OR 'low income nation*':ab,ti OR 'low-income nation*':ab,ti OR 'poor countr*':ab,ti OR 'poor nation*':ab,ti OR lmic:ab,ti OR lmics:ab,ti OR 'Africa'/exp OR 'Asia'/exp OR 'South America'/exp OR 'South and Central America'/exp OR 'Central America'/exp OR 'Central American'/exp OR africa:ab,ti OR asia:ab,ti OR 'south america*':ab,ti OR 'latin america*':ab,ti OR 'central america*':ab,ti OR Afghanistan*:ab,ti OR Albania*:ab,ti OR Algeria*:ab,ti OR Samoa*:ab,ti OR Angola*:ab,ti OR Armenia*:ab,ti OR Azerbaijan*:ab,ti OR Bangladesh*:ab,ti OR Bengali:ab,ti OR Belarus*:ab,ti OR Belize:ab,ti OR Benin:ab,ti OR Bhutan*:ab,ti OR Bolivia*:ab,ti OR Bosnia*:ab,ti OR Herzegovina*:ab,ti OR Botswana*:ab,ti OR Brazil*:ab,ti OR Bulgaria*:ab,ti OR 'Burkina Faso':ab,ti OR Burkinabe:ab,ti OR Burundi*:ab,ti OR 'Cabo Verd*':ab,ti OR 'Cape Verd*':ab,ti OR Cambodia*:ab,ti OR Cameroon*:ab,ti OR 'Central African*':ab,ti OR Chad*:ab,ti OR China:ab,ti OR Chinese:ab,ti OR Colombia*:ab,ti OR Comoros:ab,ti OR Congo:ab,ti OR 'Costa Rica*':ab,ti OR 'Cote d`Ivoire':ab,ti OR 'Ivory Coast':ab,ti OR Cuba:ab,ti OR Cuban:ab,ti OR Djibouti:ab,ti OR Dominica*:ab,ti OR Ecuador:ab,ti OR Egypt*:ab,ti OR 'El Salvador*':ab,ti OR Eritrea*:ab,ti OR Ethiopia*:ab,ti OR Fiji*:ab,ti OR Gabon*:ab,ti OR Gambia*:ab,ti OR Georgia*:ab,ti OR Ghana*:ab,ti OR Grenada*:ab,ti OR Guatemala*:ab,ti OR Guinea*:ab,ti OR Guyan*:ab,ti OR Haiti*:ab,ti OR Hondura*:ab,ti OR India:ab,ti OR Indian*:ab,ti OR Indonesia*:ab,ti OR Iran*:ab,ti OR Iraq*:ab,ti OR Jamaica*:ab,ti OR Jordan*:ab,ti OR Kazakh*:ab,ti OR Kenya*:ab,ti OR Kiribati:ab,ti OR 'People`s Republic of Korea':ab,ti OR 'North Korea':ab,ti OR Kosovo:ab,ti OR Kosovar*:ab,ti OR Kyrgyz*:ab,ti OR Lao:ab,ti OR Laos:ab,ti OR Laotian*:ab,ti OR Lebanon:ab,ti OR Lebanes*:ab,ti OR Lesotho:ab,ti OR Liberia*:ab,ti OR Libya*:ab,ti OR Macedonia*:ab,ti OR Madagascar*:ab,ti OR Malawi*:ab,ti OR Malaysia*:ab,ti OR Maldives:ab,ti OR Mali:ab,ti OR 'Marshall Island*':ab,ti OR 'Mexico'/exp OR Mexico:ab,ti OR Mexican*:ab,ti OR Micronesia*:ab,ti OR Moldova*:ab,ti OR Mongolia*:ab,ti OR Montenegr*:ab,ti OR Morocc*:ab,ti OR Mozambique:ab,ti OR Myanmar:ab,ti OR Burmese*:ab,ti OR Burma:ab,ti OR Namibia*:ab,ti OR Nepal*:ab,ti OR Nicaragua*:ab,ti OR Niger*:ab,ti OR Pakistan*:ab,ti OR Paraguay*:ab,ti OR Peru*:ab,ti OR Philippin*:ab,ti OR Rwanda*:ab,ti OR 'Sao Tome':ab,ti OR Principe:ab,ti OR Senegal*:ab,ti OR Serbia*:ab,ti OR 'Sierra Leone*':ab,ti OR 'Solomon Island*':ab,ti OR Somalia*:ab,ti OR 'South Africa*':ab,ti OR 'Sri Lanka':ab,ti OR 'St Lucia':ab,ti OR 'Saint Lucia':ab,ti OR 'St Vincent':ab,ti OR 'Saint Vincent':ab,ti OR Grenad*:ab,ti OR Sudan*:ab,ti OR Suriname*:ab,ti OR Swaziland*:ab,ti OR Eswatini*:ab,ti OR Syria*:ab,ti OR Tajik*:ab,ti OR Tanzania*:ab,ti OR Zanzibar:ab,ti OR Thai*:ab,ti OR Timor*:ab,ti OR Togo*:ab,ti OR Tonga*:ab,ti OR Tunisia*:ab,ti OR Turkey:ab,ti OR Turkish:ab,ti OR Turkmen*:ab,ti OR Tuvalu*:ab,ti OR Uganda*:ab,ti OR Ukrain*:ab,ti OR Uzbeki*:ab,ti OR Vanuatu*:ab,ti OR

		Venezuela*:ab,ti OR Vietnam*:ab,ti OR ‘Viet nam*’:ab,ti OR ‘West Bank’:ab,ti OR Gaza*:ab,ti OR Palestin*:ab,ti OR Yemen*:ab,ti OR Zambia*:ab,ti OR Zimbabw*:ab,ti OR ‘Western Sahara’:ab,ti OR Argentin*:ab,ti OR Russia*:ab,ti OR Maurit*:ab,ti OR palau:ab,ti
12	Animal studies	'animal'/exp NOT ('animal'/exp AND 'human'/exp)
Total	(1 OR 2) AND (3 OR 4 OR 5 OR 6) AND (7 OR 8 OR 9) AND 10 AND 11 NOT 12	

Supplemental Table 3. Web of Science search strategy for the impact of nutrition on reproductive outcomes of women in low- and middle-income countries

No.	Concept	Search terms
1	Intervention Studies	TS="clinical trial*" OR TS="randomized controlled trial*" OR TS=intervention* OR TS=random* OR TS=trial*
2	Cohort Studies	TS="cohort Stud*" OR TS=cohort* OR TS=prospective OR TS=longitudinal
3	Micronutrients	TS=micronutrient* OR TS=Mineral* OR TS=vitamin* OR TS="Dietary Supplement*" OR TS=calcium OR TS=magnesium OR TS=phosphorus OR TS=potassium OR TS=boron OR TS=cobalt OR TS=chromium OR TS=copper OR TS=iodine OR TS=iron OR TS=ferritin OR TS=transferrin OR TS=hematocrit OR TS=haematocrit OR TS=manganese OR TS=molybdenum OR TS=selenium OR TS= zinc OR TS=thiamin* OR TS=riboflavin OR TS=niacin OR TS="pantothenic acid" OR TS=pyridox* OR TS=biotin OR TS=folate OR TS="folic acid" OR TS="Vitamin B-12" OR TS="Vitamin B12" OR TS=cobalamin* OR TS="methylmalonic acid*" OR TS= homocysteine OR TS="vitamin A" OR TS=retinol OR TS="ascorbic acid" OR TS=calciferol* OR TS=ergocalciferol* OR TS="Vitamin D" OR TS=cholecalciferol OR TS=tocopherol* OR TS=tocotrienol* OR TS="Vitamin K" OR TS=phylloquinone OR TS=menaquinone OR TS=Choline OR TS=carotenoid* OR TS=carotene OR TS=hemoglobin* OR TS=haemoglobin* OR TS=anemia OR TS=anaemia OR TS=anemic OR TS=anaemic
4	Macronutrients	TS=macronutrient* OR TS=carbohydrate* OR TS=glucose OR TS= fiber* OR TS=fat OR TS="fatty acid*" OR TS="omega-3" OR TS="omega-6" OR TS=lipid* OR TS=triglyceride* OR TS=cholesterol* OR TS=protein* OR TS="amino acid*"
5	Dietary intake (generic terms)	TS=nutrient* OR TS=diet OR TS=dietary OR TS=food* OR TS=eating OR TS="energy intake" OR TS=caloric OR TS=calorie
6	Anthropometry	TS="body weight" OR TS=Weight OR TS="body mass index" OR TS=BMI OR TS=obesity OR TS=obese OR TS="Thinness" OR TS=underweight OR TS=malnutrition OR TS=malnourished OR TS=undernutrition OR TS=undernourished OR TS="weight gain" OR TS="gestational weight gain" OR TS=overweight OR TS=anthropometry OR TS=anthropometric OR TS=height OR TS=stature OR TS=stunting OR TS=stunted OR TS=overnutrition OR TS="over-nutrition" OR TS="nutritional status" OR TS=nutrition OR TS="BMI-for-age" OR TS="height-for-age" OR TS="weight-for-age"
7	Fertility, fecundity, menarche, menstruation	TS=fertility OR TS=fecundity OR TS=infecundity OR TS=fecundability OR TS="number of pregnancies" OR TS="number of children" OR TS="number of offspring" OR TS=fertilization OR TS=fertilisation OR TS=infertility OR TS=sterility OR TS=conception OR TS=ovulation OR TS=imbedding OR TS=implantation OR TS=menarche OR TS=menstrua* OR TS=amenorrh* OR TS=oligomenorrh* OR TS=polymenorrh* OR TS=menorrhagia
8	Miscarriage	TS=miscarriage* OR TS="spontaneous abortion*" OR TS="pregnancy loss"
9	Stillbirth	TS=stillbirth* OR TS="fetal death*" OR TS="fetal loss*"
10	Adolescent girls and women	TS=women OR TS=woman OR TS=Adolescent* OR TS=teen OR TS=teens OR TS=teenager* OR TS=girl* OR TS=mother*
11	Low- and middle-income countries	TS="developing countr*" OR TS= "developing nation*" OR TS= "less developed countr*" OR TS= "less developed nation*" OR TS= "third world nation*" OR TS= "third world countr*" OR TS= "under developed nation*" OR TS= "underdeveloped nation*" OR TS= "under developed countr*" OR TS= "underdeveloped countr*" OR TS= "middle income countr*" OR TS= "middle-income countr*" OR TS= "middle income nation*" OR TS="middle-income nation*" OR TS= "low income countr*" OR TS= "low-income countr*" OR TS= "low income nation*" OR TS= "low-income nation*" OR TS= "poor countr*" OR TS= "poor nation*" OR TS=LMIC OR TS=LMICs OR TS="Africa" OR TS="Asia" OR TS="South America" OR TS="Latin America" OR TS="Central America" OR TS=africa OR TS=asia OR TS= "south america*" OR TS= "latin america*" OR TS= "central america*" OR TS=Afghanistan* OR TS=Albania* OR TS=Algeria* OR TS=Samoa* OR TS=Angola* OR TS=Armenia* OR TS=Azerbaijan* OR TS=Bangladesh* OR TS=Bengali OR TS=Belarus* OR TS=Belize OR TS=Benin OR TS=Bhutan* OR TS=Bolivia* OR TS=Bosnia* OR TS=Herzegovina* OR TS=Botswana* OR TS=Brazil* OR TS=Bulgaria* OR TS= "Burkina Faso" OR TS=Burkinabe OR TS=Burundi* OR TS= "Cabo Verd*" OR TS= "Cape Verd*" OR TS=Cambodia* OR TS=Cameroon* OR TS="Central African*" OR TS=Chad* OR TS=China OR TS=Chinese OR TS=Colombia* OR TS=Comoros OR TS=Congo OR TS= "Costa Rica*" OR TS= "Cote d'Ivoire" OR TS= "Ivory Coast" OR TS=Cuba OR TS=Cuban OR TS=Djibouti OR TS=Dominica* OR TS=Ecuador OR TS=Egypt* OR TS= "El Salvador*" OR TS=Eritrea* OR TS=Ethiopia* OR TS=Fiji* OR TS=Gabon* OR TS=Gambia* OR TS=Georgia* OR TS=Ghana* OR TS=Grenada* OR TS=Guatemala* OR TS=Guinea* OR TS=Guyan* OR TS=Haiti* OR TS=Hondura* OR TS=India OR TS=Indian* OR TS=Indonesia* OR TS=Iran* OR TS=Iraq* OR TS=Jamaica* OR TS=Jordan* OR TS=Kazakh* OR TS=Kenya* OR TS=Kiribati OR TS= "People's Republic of Korea" OR TS="North Korea" OR TS=Kosovo OR TS=Kosovar* OR TS=Kyrgyz* OR TS=Lao OR TS=Laos OR TS=Laotian* OR TS=Lebanon OR TS=Lebanes* OR TS=Lesotho OR TS=Liberia* OR TS=Libya* OR TS=Macedonia* OR TS=Madagascar* OR TS=Malawi* OR TS=Malaysia* OR TS=Maldives OR TS=Mali OR TS= "Marshall Island*" OR TS=Mexico OR TS=Mexican* OR TS=Micronesia* OR TS=Moldova* OR TS=Mongolia* OR TS=Montenegr* OR TS=Morocc* OR TS=Mozambique OR TS=Myanmar OR TS=Burmese* OR TS=Burma OR TS=Namibia* OR TS=Nepal* OR TS=Nicaragua* OR TS=Niger* OR TS=Pakistan* OR TS=Paraguay* OR TS=Peru* OR TS=Philippin* OR TS=Rwanda* OR TS= "Sao Tome" OR TS=Principe OR TS=Senegal* OR TS=Serbia* OR TS="Sierra Leone*" OR TS="Solomon Island*" OR TS=Somalia* OR TS= "South Africa*" OR TS= "Sri Lanka" OR TS= "St Lucia" OR TS= "Saint Lucia" OR TS="St Vincent" OR TS="Saint Vincent" OR TS=Grenad* OR TS=Sudan* OR TS=Suriname* OR TS=Swaziland* OR TS=Eswatini* OR TS=Syria* OR TS=Tajik* OR TS=Tanzania* OR TS=Zanzibar OR TS=Thai* OR TS=Timor* OR TS=Togo* OR TS=Tonga* OR TS=Tunisia* OR TS=Turkey OR TS=Turkish OR TS=Turkmen* OR TS=Tuvalu* OR TS=Uganda* OR TS=Ukrain* OR TS=Uzbeki* OR TS=Vanuatu* OR TS=Venezuela* OR TS=Vietnam* OR TS= "Viet nam*" OR TS= "West Bank" OR TS=Gaza* OR TS=Palestin* OR TS=Yemen* OR TS=Zambia* OR TS=Zimbabw* OR TS= "Western Sahara" OR TS=Argentin* OR TS=Russia* OR TS=Maurit* OR TS=Palau
12	Animal studies	TS= "Animals" NOT (TS= "Animals" AND TS="Humans")
Total	(1 OR 2) AND (3 OR 4 OR 5 OR 6) AND (7 OR 8 OR 9) AND 10 AND 11 NOT 12	

Supplemental Table 4. Cochrane Library search strategy for the impact of nutrition on reproductive outcomes of women in low- and middle-income countries

No.	Concept	Search terms	
A	Intervention Studies	1	MeSH descriptor: [Clinical Trials as Topic] explode all trees
		2	("randomized controlled trial"):pt
		3	(clinical trial):pt
		4	("controlled clinical trial"):pt
		5	MeSH descriptor: [Randomized Controlled Trials as Topic] explode all trees
		6	(intervention*):ti,ab,kw
		7	MeSH descriptor: [Random Allocation] explode all trees
		8	(random*):ti,ab,kw
		9	(trial*):ti,ab,kw
		10	MeSH descriptor: [Clinical Trial Protocols as Topic] explode all trees
		11	("clinical trial protocol"):pt
		12	("clinical study"):pt
		13	MeSH descriptor: [Clinical Studies as Topic] explode all trees
		14	MeSH descriptor: [Therapeutic Uses] explode all trees
		15	MeSH descriptor: [] explode all trees and with qualifier(s): [therapeutic use - TU]
		16	OR 1-15
B	Cohort Studies	17	MeSH descriptor: [Cohort Studies] explode all trees
		18	(cohort*):ti,ab,kw
		19	(prospective):ti,ab,kw
		20	(longitudinal):ti,ab,kw
		21	OR 17-20
C	Micronutrients	22	MeSH descriptor: [Micronutrients] explode all trees
		23	(micronutrient*):ti,ab,kw
		24	MeSH descriptor: [Minerals] explode all trees
		25	(mineral*):ti,ab,kw
		26	MeSH descriptor: [Vitamins] explode all trees
		27	(vitamin*):ti,ab,kw
		28	MeSH descriptor: [Dietary Supplements] explode all trees
		29	MeSH descriptor: [Calcium] explode all trees
		30	(calcium):ti,ab,kw
		31	MeSH descriptor: [Magnesium] explode all trees
		32	(magnesium):ti,ab,kw
		33	MeSH descriptor: [Phosphorus, Dietary] explode all trees
		34	(potassium):ti,ab,kw
		35	MeSH descriptor: [Boron] explode all trees
		36	(boron):ti,ab,kw
		37	MeSH descriptor: [Cobalt] explode all trees
		38	(cobalt):ti,ab,kw
		39	MeSH descriptor: [Chromium] explode all trees
		40	(chromium):ti,ab,kw
		41	MeSH descriptor: [Copper] explode all trees
		42	(copper):ti,ab,kw
		43	MeSH descriptor: [Iodine] explode all trees
		44	(iodine):ti,ab,kw
		45	MeSH descriptor: [Iron] explode all trees
		46	(iron):ti,ab,kw
		47	MeSH descriptor: [Ferritins] explode all trees
		48	(ferritin):ti,ab,kw
		49	MeSH descriptor: [Transferrins] explode all trees
		50	(transferrin):ti,ab,kw
		51	MeSH descriptor: [Hematocrit] explode all trees
		52	(hematocrit):ti,ab,kw
		53	(haematocrit):ti,ab,kw
		54	MeSH descriptor: [Manganese] explode all trees
		55	(manganese):ti,ab,kw
		56	MeSH descriptor: [Molybdenum] explode all trees
		57	(molybdenum):ti,ab,kw
		58	MeSH descriptor: [Selenium] explode all trees
		59	(selenium):ti,ab,kw
		60	MeSH descriptor: [Zinc] explode all trees
		61	(zinc):ti,ab,kw
		62	MeSH descriptor: [Thiamine] explode all trees
		63	(thiamine):ti,ab,kw
		64	MeSH descriptor: [Riboflavin] explode all trees
		65	(riboflavin):ti,ab,kw
		66	MeSH descriptor: [Niacin] explode all trees
		67	(niacin):ti,ab,kw
		68	MeSH descriptor: [Pantothenic Acid] explode all trees
		69	("pantothenic acid"):ti,ab,kw
		70	MeSH descriptor: [Pyridoxine] explode all trees
		71	(pyridox*):ti,ab,kw

		72	MeSH descriptor: [Biotin] explode all trees
		73	(biotin):ti,ab,kw
		74	MeSH descriptor: [Folic Acid] explode all trees
		75	(folate):ti,ab,kw
		76	("folic acid"):ti,ab,kw
		77	MeSH descriptor: [Vitamin B 12] explode all trees
		78	(cobalamin*):ti,ab,kw
		79	MeSH descriptor: [Methylmalonic Acid] explode all trees
		80	("Methylmalonic Acid*"):ti,ab,kw
		81	MeSH descriptor: [Homocysteine] explode all trees
		82	(homocysteine):ti,ab,kw
		83	MeSH descriptor: [Vitamin A] explode all trees
		84	(retinol):ti,ab,kw
		85	MeSH descriptor: [Ascorbic Acid] explode all trees
		86	("ascorbic acid"):ti,ab,kw
		87	(calciferol*):ti,ab,kw
		88	MeSH descriptor: [Ergocalciferols] explode all trees
		89	(ergocalciferol):ti,ab,kw
		90	MeSH descriptor: [Vitamin D] explode all trees
		91	(cholecaliferol):ti,ab,kw
		92	MeSH descriptor: [Tocopherols] explode all trees
		93	(tocopherol*):ti,ab,kw
		94	MeSH descriptor: [Tocotrienols] explode all trees
		95	(tocotrienol*):ti,ab,kw
		96	MeSH descriptor: [Vitamin K] explode all trees
		97	("phylloquinone"):ti,ab,kw
		98	(menaquinone):ti,ab,kw
		99	MeSH descriptor: [Choline] explode all trees
		100	(choline):ti,ab,kw
		101	MeSH descriptor: [Carotenoids] explode all trees
		102	(carotenoid*):ti,ab,kw
		103	(carotene):ti,ab,kw
		104	MeSH descriptor: [Hemoglobins] explode all trees
		105	(hemoglobin*):ti,ab,kw
		106	(haemoglobin*):ti,ab,kw
		107	MeSH descriptor: [Anemia] explode all trees
		108	(anemia):ti,ab,kw
		109	(anaemia):ti,ab,kw
		110	(anemic):ti,ab,kw
		111	(anaemic):ti,ab,kw
		112	OR 22-111
D	Macronutrients	113	(macronutrient*):ti,ab,kw
		114	MeSH descriptor: [Carbohydrates] explode all trees
		115	(carbohydrate*):ti,ab,kw
		116	MeSH descriptor: [Glucose] explode all trees
		117	(glucose):ti,ab,kw
		118	MeSH descriptor: [Dietary Fiber] explode all trees
		119	(fiber*):ti,ab,kw
		120	(fat):ti,ab,kw
		121	MeSH descriptor: [Fatty Acids] explode all trees
		122	("fatty acid*"):ti,ab,kw
		123	("Omega-3"):ti,ab,kw
		124	("Omega-6"):ti,ab,kw
		125	MeSH descriptor: [Lipids] explode all trees
		126	(lipid*):ti,ab,kw
		127	MeSH descriptor: [Triglycerides] explode all trees
		128	(triglyceride*):ti,ab,kw
		129	MeSH descriptor: [Cholesterol] explode all trees
		130	(cholesterol*):ti,ab,kw
		131	MeSH descriptor: [Proteins] explode all trees
		132	(protein*):ti,ab,kw
		133	MeSH descriptor: [Amino Acids] explode all trees
		134	("amino acid*"):ti,ab,kw
		135	OR 113-134
E	Dietary intake (generic terms)	136	MeSH descriptor: [Nutrients] explode all trees
		137	(nutrient*):ti,ab,kw
		138	MeSH descriptor: [Diet] explode all trees
		139	(diet):ti,ab,kw
		140	(dietary):ti,ab,kw
		141	MeSH descriptor: [Food] explode all trees
		142	(food*):ti,ab,kw
		143	MeSH descriptor: [Eating] explode all trees
		144	(eating):ti,ab,kw
		145	MeSH descriptor: [Energy Intake] explode all trees
		146	(caloric):ti,ab,kw

		147	(calorie):ti,ab,kw
		148	OR 136-147
F	Anthropometry	149	MeSH descriptor: [Body Weight] explode all trees
		150	(weight):ti,ab,kw
		151	MeSH descriptor: [Body Mass Index] explode all trees
		152	("body mass index"):ti,ab,kw
		153	(BMI):ti,ab,kw
		154	MeSH descriptor: [Obesity] explode all trees
		155	(obesity):ti,ab,kw
		156	(obese):ti,ab,kw
		157	MeSH descriptor: [Thinness] explode all trees
		158	(underweight):ti,ab,kw
		159	MeSH descriptor: [Malnutrition] explode all trees
		160	(malnutrition):ti,ab,kw
		161	(malnourished):ti,ab,kw
		162	(undernutrition):ti,ab,kw
		163	(undernourished):ti,ab,kw
		164	MeSH descriptor: [Weight Gain] explode all trees
		165	MeSH descriptor: [Gestational Weight Gain] explode all trees
		166	MeSH descriptor: [Body Weight Changes] explode all trees
		167	MeSH descriptor: [Body-Weight Trajectory] explode all trees
		168	MeSH descriptor: [Overweight] explode all trees
		169	(overweight):ti,ab,kw
		170	MeSH descriptor: [Anthropometry] explode all trees
		171	(anthropometry):ti,ab,kw
		172	(anthropometric):ti,ab,kw
		173	MeSH descriptor: [Body Height] explode all trees
		174	(height):ti,ab,kw
		175	(stature):ti,ab,kw
		176	(stunting):ti,ab,kw
		177	(stunted):ti,ab,kw
		178	MeSH descriptor: [Overnutrition] explode all trees
		179	(overnutrition):ti,ab,kw
		180	("over-nutrition"):ti,ab,kw
		181	MeSH descriptor: [Nutritional Status] explode all trees
		182	(nutrition):ti,ab,kw
		183	("BMI-for-age"):ti,ab,kw
		184	("height-for-age"):ti,ab,kw
		185	("weight-for-age"):ti,ab,kw
		186	OR 149-185
G	Fertility, fecundity, menarche, menstruation	187	(fertility):ti,ab,kw
		188	(fecundity):ti,ab,kw
		189	(infecundity):ti,ab,kw
		190	(fecundability):ti,ab,kw
		191	("number of pregnancies"):ti,ab,kw
		192	("number of children"):ti,ab,kw
		193	("number of offspring"):ti,ab,kw
		194	(fertilization):ti,ab,kw
		195	(fertilisation):ti,ab,kw
		196	MeSH descriptor: [Infertility] explode all trees
		197	(infertility):ti,ab,kw
		198	(sterility):ti,ab,kw
		199	(conception):ti,ab,kw
		200	MeSH descriptor: [Ovulation] explode all trees
		201	(ovulation):ti,ab,kw
		202	(imbedding):ti,ab,kw
		203	MeSH descriptor: [Embryo Implantation] explode all trees
		204	(implantation):ti,ab,kw
		205	MeSH descriptor: [Menarche] explode all trees
		206	(menarche):ti,ab,kw
		207	MeSH descriptor: [Menstruation] explode all trees
		208	(menstrua*):ti,ab,kw
		209	(amenorrh*):ti,ab,kw
		210	(oligomenorrh*):ti,ab,kw
		211	(polymenorrh*):ti,ab,kw
		212	(menorrhagia):ti,ab,kw
		213	OR 187-212
H	Miscarriage	214	MeSH descriptor: [Abortion, Spontaneous] explode all trees
		215	(miscarriage*):ti,ab,kw
		216	("spontaneous abortion*"):ti,ab,kw
		217	("pregnancy loss"):ti,ab,kw
		218	OR 214-217
I	Stillbirth	219	MeSH descriptor: [Stillbirth] explode all trees
		220	(stillbirth*):ti,ab,kw
		221	MeSH descriptor: [Fetal Death] explode all trees

		222	("fetal death*"):ti,ab,kw
		223	("fetal loss"):ti,ab,kw
		224	OR 219-223
J	Adolescent girls and women	233	MeSH descriptor: [Women] explode all trees
		234	(women):ti,ab,kw
		235	(woman):ti,ab,kw
		236	MeSH descriptor: [Adolescent] explode all trees
		237	(adolescent*):ti,ab,kw
		238	(teen):ti,ab,kw
		239	(teens):ti,ab,kw
		240	(teenager*):ti,ab,kw
		241	(girl*):ti,ab,kw
		242	MeSH descriptor: [Mothers] explode all trees
		243	(mother*):ti,ab,kw
		244	OR 233-243
K	Low- and middle-income countries	245	MeSH descriptor: [Developing Countries] explode all trees
		246	("developing countr*"):ti,ab,kw
		247	("developing nation*"):ti,ab,kw
		248	("less developed countr*"):ti,ab,kw
		249	("less developed nation*"):ti,ab,kw
		250	("third world nation*"):ti,ab,kw
		251	("third world countr*"):ti,ab,kw
		252	("under developed nation*"):ti,ab,kw
		253	("underdeveloped nation*"):ti,ab,kw
		254	("under developed countr*"):ti,ab,kw
		255	("underdeveloped countr*"):ti,ab,kw
		256	("middle income countr*"):ti,ab,kw
		257	("middle-income countr*"):ti,ab,kw
		258	("middle income nation*"):ti,ab,kw
		259	("middle-income nation*"):ti,ab,kw
		260	("low income countr*"):ti,ab,kw
		261	("low-income countr*"):ti,ab,kw
		262	("low income nation*"):ti,ab,kw
		263	("low-income nation*"):ti,ab,kw
		264	("poor countr*"):ti,ab,kw
		265	("poor nation*"):ti,ab,kw
		266	(LMIC):ti,ab,kw
		267	(LMICs):ti,ab,kw
		268	MeSH descriptor: [Africa] explode all trees
		269	MeSH descriptor: [Asia] explode all trees
		270	MeSH descriptor: [South America] explode all trees
		271	MeSH descriptor: [Latin America] explode all trees
		272	MeSH descriptor: [Central America] explode all trees
		273	(africa):ti,ab,kw
		274	(asia):ti,ab,kw
		275	("south america*"):ti,ab,kw
		276	("latin america*"):ti,ab,kw
		277	("central america*"):ti,ab,kw
		278	(Afghanistan*):ti,ab,kw
		279	(Albania*):ti,ab,kw
		280	(Algeria*):ti,ab,kw
		281	(Samoa*):ti,ab,kw
		282	(Angola*):ti,ab,kw
		283	(Armenia*):ti,ab,kw
		284	(Azerbaijan*):ti,ab,kw
		285	(Bangladesh*):ti,ab,kw
		286	(Bengali):ti,ab,kw
		287	(Belarus*):ti,ab,kw
		288	(Belize):ti,ab,kw
		289	(benin):ti,ab,kw
		290	(Bhutan*):ti,ab,kw
		291	(Bolivia*):ti,ab,kw
		292	(Bosnia*):ti,ab,kw
		293	(Herzegovina*):ti,ab,kw
		294	(Botswana*):ti,ab,kw
		295	(Brazil*):ti,ab,kw
		296	(Bulgaria*):ti,ab,kw
		297	("Burkina Faso"):ti,ab,kw
		298	(Burkinabe):ti,ab,kw
		299	(Burundi*):ti,ab,kw
		300	("Cabo Verd*"):ti,ab,kw
		301	("Cape Verd*"):ti,ab,kw
		302	(Cambodia*):ti,ab,kw
		303	(Cameroon*):ti,ab,kw
		304	("Central African*"):ti,ab,kw

		305	(Chad*):ti,ab,kw
		306	(China):ti,ab,kw
		307	(Chinese):ti,ab,kw
		308	(Colombia*):ti,ab,kw
		309	(comoros):ti,ab,kw
		310	(Congo):ti,ab,kw
		311	("Costa Rica*"):ti,ab,kw
		312	("Cote d'Ivoire"):ti,ab,kw
		313	("Ivory Coast"):ti,ab,kw
		314	(Cuba):ti,ab,kw
		315	(Cuban):ti,ab,kw
		316	(Djibouti):ti,ab,kw
		317	(Dominica*):ti,ab,kw
		318	(Ecuador):ti,ab,kw
		319	(Egypt*):ti,ab,kw
		320	("El Salvador*"):ti,ab,kw
		321	(Eritrea*):ti,ab,kw
		322	(Ethiopia*):ti,ab,kw
		323	(Fiji*):ti,ab,kw
		324	(Gabon*):ti,ab,kw
		325	(Gambia*):ti,ab,kw
		326	(Georgia*):ti,ab,kw
		327	(Ghana*):ti,ab,kw
		328	(Grenada*):ti,ab,kw
		329	(Guatemala*):ti,ab,kw
		330	(Guinea*):ti,ab,kw
		331	(Guyan*):ti,ab,kw
		332	(Haiti*):ti,ab,kw
		333	(Hondura*):ti,ab,kw
		334	(India):ti,ab,kw
		335	(Indian*):ti,ab,kw
		336	(Indonesia*):ti,ab,kw
		337	(Iran*):ti,ab,kw
		338	(Iraq*):ti,ab,kw
		339	(Jamaica*):ti,ab,kw
		340	(Jordan*):ti,ab,kw
		341	(Kazakh*):ti,ab,kw
		342	(Kenya*):ti,ab,kw
		343	(Kiribati):ti,ab,kw
		344	("People's Republic of Korea"):ti,ab,kw
		345	("North Korea"):ti,ab,kw
		346	(Kosovo):ti,ab,kw
		347	(Kosovar*):ti,ab,kw
		348	(Kyrgyz*):ti,ab,kw
		349	(Lao):ti,ab,kw
		350	(Laos):ti,ab,kw
		351	(Laotian*):ti,ab,kw
		352	(Lebanon):ti,ab,kw
		353	(Lebanes*):ti,ab,kw
		354	(Lesotho):ti,ab,kw
		355	(Liberia*):ti,ab,kw
		356	(Libya*):ti,ab,kw
		357	(Macedonia*):ti,ab,kw
		358	(Madagascar*):ti,ab,kw
		359	(Malawi*):ti,ab,kw
		360	(Malaysia*):ti,ab,kw
		361	(Maldives):ti,ab,kw
		362	(Mali):ti,ab,kw
		363	("Marshall Island*"):ti,ab,kw
		364	(Mexico):ti,ab,kw
		365	MeSH descriptor: [Mexico] explode all trees
		366	(Mexican*):ti,ab,kw
		367	(Micronesia*):ti,ab,kw
		368	(Moldova*):ti,ab,kw
		369	(Mongolia*):ti,ab,kw
		370	(Montenegr*):ti,ab,kw
		371	(Morocc*):ti,ab,kw
		372	(Mozambique):ti,ab,kw
		373	(Myanmar):ti,ab,kw
		374	(Burmese*):ti,ab,kw
		375	(Burma):ti,ab,kw
		376	(Namibia*):ti,ab,kw
		377	(Nepal*):ti,ab,kw
		378	(Nicaragua*):ti,ab,kw
		379	(Niger*):ti,ab,kw

		380	(Pakistan*):ti,ab,kw
		381	(Paraguay*):ti,ab,kw
		382	(Peru*):ti,ab,kw
		383	(Philippin*):ti,ab,kw
		384	(Rwanda*):ti,ab,kw
		385	("Sao Tome"):ti,ab,kw
		386	(Principe):ti,ab,kw
		387	(Senegal*):ti,ab,kw
		388	(Serbia):ti,ab,kw
		389	("Sierra Leone*"):ti,ab,kw
		390	("Solomon Island*"):ti,ab,kw
		391	(Somalia*):ti,ab,kw
		392	("South Africa*"):ti,ab,kw
		393	("Sri Lanka"):ti,ab,kw
		394	("St Lucia"):ti,ab,kw
		395	("Saint Lucia"):ti,ab,kw
		396	("St Vincent"):ti,ab,kw
		397	("Saint Vincent"):ti,ab,kw
		398	(Grenad*):ti,ab,kw
		399	(Sudan*):ti,ab,kw
		400	(Suriname*):ti,ab,kw
		401	(Swaziland*):ti,ab,kw
		402	(Eswatini*):ti,ab,kw
		403	(Syria*):ti,ab,kw
		404	(Tajik*):ti,ab,kw
		405	(Tanzania*):ti,ab,kw
		406	(Zanzibar):ti,ab,kw
		407	(Thai*):ti,ab,kw
		408	(Timor*):ti,ab,kw
		409	(Togo*):ti,ab,kw
		410	(Tonga*):ti,ab,kw
		411	(Tunisia*):ti,ab,kw
		412	(Turkey):ti,ab,kw
		413	(Turkish):ti,ab,kw
		414	(Turkmen*):ti,ab,kw
		415	(Tuvalu*):ti,ab,kw
		416	(Uganda*):ti,ab,kw
		417	(Ukrain*):ti,ab,kw
		418	(Uzbeki*):ti,ab,kw
		419	(Vanuatu*):ti,ab,kw
		420	(Venezuela*):ti,ab,kw
		421	(Vietnam*):ti,ab,kw
		422	("Viet nam*"):ti,ab,kw
		423	("West Bank"):ti,ab,kw
		424	(Gaza*):ti,ab,kw
		425	(Palestin*):ti,ab,kw
		426	(Yemen*):ti,ab,kw
		427	(Zambia*):ti,ab,kw
		428	(Zimbabw*):ti,ab,kw
		429	("Western Sahara"):ti,ab,kw
		430	("Argentin*"):ti,ab,kw
		431	("Russia*"):ti,ab,kw
		432	(Maurit*):ti,ab,kw
		433	(Palau):ti,ab,kw
		434	OR 245-433
Total	(A OR B) AND (C OR D OR E OR F) AND (G OR H OR I) AND J AND K		

Supplemental Table 5. Characteristics of the included intervention studies

Study	Setting	Study design	Time period	Study population	Number of participants in analysis	Age[1]	Intervention	Frequency and dosage	Control	Outcomes of interest	Overall findings
Khan 1995[1]	Guatemala	Cluster RCT	1969-1992	Women residents born between 1962 and 1977 in four villages	806	Range: 15-30	Atole (high energy and high protein supplement) from childhood until 7 yrs of age	163 kcal/682 kJ and 11.5 g protein per cup of 180 mL	Fresco (low energy, no protein supplement) providing 59 kcal/247 kJ in 180 mL	Age at menarche	Supplement type was not a significant predictor of age at menarche
Ceesay 1997[2]	West Kiang, Gambia	Cluster RCT	1989-1994	Women chronically undernourished in 28 villages	1460	15-45	Dietary supplementation with high-energy groundnut biscuits from around 20 wks gestation	Two biscuits containing 4250 kJ energy, 22 g protein, 56 g fat, 47 mg calcium, and 1.8 mg iron daily	No intervention	Stillbirths	Supplementation reduced stillbirths (OR: 0.47; 95% CI: 0.23 to 0.99, $p < 0.05$)
Fawzi 1998[3]	Dar es Salaam, Tanzania	RCT[2]	1995-1997	HIV-infected pregnant women 12-27 wks gestation	1075	Not clear	Vitamin A, multivitamins without vitamin A, and multivitamins with vitamin A from pregnancy to delivery	Vitamin A: 30 mg of carotene plus 5000 IU preformed vitamin per day; Multivitamins: 20 mg B1, 20 mg B2, 25 mg B6, 100 mg niacin, 50 µg B12, 500 mg C, 30 mg E, and 0.8 mg FA per day	Placebo	Miscarriage (< 28 wks); stillbirth (≥ 28 wks); fetal death combining the two	MMS and vitamin A did not result in significant differences in miscarriage, stillbirth, or fetal death
Katz 2000[4]	Sarlahi District, Nepal	Cluster RCT	1994-1997	Newly married women of reproductive age from 270 wards (90 for each study arm)	15832	Not clear but categorized as < 20, 20-29, 30+	Vitamin A and B-carotene supplementation during preconception and pregnancy	Vitamin A (7000 μg; weekly) and B-carotene (42 mg; weekly) All capsules contained 5 mg dl-a-tocopherol	Placebo	Fetal death, miscarriage, and stillbirth	There was no significant effect of either vitamin A or B-carotene supplement on fetal loss
Christian 2003[5]	Sarlahi, Nepal	Cluster RCT	1999-2001	Pregnant women	4926	Not clear but categorized as < 20, 20-24, 25-29, 30-35, > 35	FA, FA+ iron, FA + iron + zinc, or MMS from the time of pregnancy detection through 3 mo postpartum in the case of a live birth and through ≥ 5 wks after a miscarriage or stillbirth; all also received vitamin A	One caplet per day	Vitamin A only (1000g retinol equivalents)	Miscarriage (< 28 wks)	The rates of miscarriage did not differ by treatment group
Friis 2004[6]	Harare, Zimbabwe	RCT	1996-1997	Pregnant women 22-36 wks gestation	1106	Mean: 24.2	MMS during pregnancy	Daily tablet containing approximately the RDA for pregnant or lactating women of 13 micronutrients	Placebo	Stillbirth	There were no significant differences between the MMS and placebo groups in stillbirths ($p = 0.39$)

Study	Setting	Study design	Time period	Study population	Number of participants in analysis	Age[1]	Intervention	Frequency and dosage	Control	Outcomes of interest	Overall findings
Fawzi 2005[7]	Dar es Salaam, Tanzania	RCT	2000-2002	HIV-infected pregnant women 12-27 wks gestation	400	Zinc group: 26.7 (4.9); placebo group: 27.0 (5.0)	Zinc supplementation from enrollment until the end of the study at 6 wks postpartum	Daily oral dose of either 25 mg zinc as zinc sulfate	Placebo	Miscarriage (< 28 wks); stillbirth (≥ 28 wks); fetal death combining the two	Compared with the placebo, zinc had no significant effect on fetal loss (RR: 1.39; 95% CI: 0.58, 3.86; *p* = 0.36)
Kæstel 2005[8]	Bissau, Guinea-Bissau	RCT	2001-2002	Pregnant women < 37 wks gestation	1670	Median (Q1, Q3): 23 (20, 28)	MMS during pregnancy	Daily MMS tablet containing one of 15 micronutrients (MN-1); daily MMS tablet containing two RDA (MN-2), except for iron, which was kept at one RDA	IFA	Miscarriage (< 28 wks)	There was a significantly lower risk of miscarriage in the MN-2 group (OR: 0.14; 95% CI: 0.031, 0.61) but not in the MN-1 group (OR: 0.93; 95% CI: 0.43, 2.00) compared to IFA
Osrin 2005[9]	Dhanusha and Mahottari Districts, Nepal	RCT	2002-2004	Pregnant women 12-20 wks gestation	1139	Not clear but categorized as < 20, 20-29, ≥ 30	MMS from a minimum of 12 wks gestation until delivery	Daily MMS tablets of the UNIMMAP formulation	IFA	Stillbirth (≥ 23 wks)	More stillbirths were reported in the control group than intervention group, but the difference was not significant.
Qublan 2007[10]	Irbid, Jordan	RCT	2003-2005	Patients with PCOS with a duration of infertility > 2 yrs and BMI > 29	46	Weight reduction group - mean (range): 31.5 (19-38); metformin treatment group - Mean (range): 30.8 (20-37)	Weight reduction diet and that continued until the patient resumed the first regular cycle (with a maximum of 6 mos if there was no resumption of regular cycle and no evidence of ovulation)	Weight reduction diet providing 1200-1400 kcal/d (25% proteins, 25% fat, and 50% carbohydrates plus 25-30 g of fiber per wk)	Metformin treatment	Ovulation rate and pregnancy rate	There were no significant differences between the two groups in duration of infertility; clinical and biochemical pregnancy rates and ovulation rates were similar in the two groups
Fawzi 2007[11]	Dar es Salaam, Tanzania	RCT	2001-2004	HIV-negative pregnant women 12-27 wks gestation	8379	MMS group: 25.2 (5.1); placebo group: 25.1 (5.1)	Multivitamin supplement from time of enrollment until 6 wks after delivery	Daily oral dose of multivitamins including 20 mg of vitamin B1, 20 mg of vitamin B2, 25 mg of vitamin B6, 100 mg of niacin, 50 μg of vitamin B12, 500 mg of vitamin C, 30 mg of vitamin E, and 0.8 mg of FA	Placebo	Miscarriage (< 28 wks), stillbirth (≥ 28 wks), and fetal loss combing the two	Multivitamin supplementation had no significant effects on miscarriage (RR: 0.96; 95% CI: 0.65, 1.41), stillbirth (RR: 0.84; 95% CI: 0.67, 1.05), or fetal loss (RR: 0.87; 95% CI: 0.72, 1.05)
Zagré 2007[12]	Mayahi, Niger	Cluster RCT	2004-2006	Pregnant women < 28 wks gestation	2902	Range: 13-45; mean (SD): 25 (6)	MMS during pregnancy	Daily MMS tablets of UNIMMAP formulation	IFA	Miscarriage (< 24 wks) and stillbirth	The incidence of miscarriages was comparable between groups
Kupka 2008[13]	Dar es Salaam, Tanzania	RCT	2003-2005	HIV-infected pregnant women 12-27 wks gestation	915	27.5 (4.9)	Selenium supplementation from recruitment until 6 mos after delivery	Daily tablet of 200 g elemental selenium	Placebo	Fetal death, including miscarriage (< 28 wks) and stillbirth (≥ 28 wks)	Selenium supplements did not significantly affect the risk of fetal death (*p* = 0.08)

Study	Setting	Study design	Time period	Study population	Number of participants in analysis	Age[1]	Intervention	Frequency and dosage	Control	Outcomes of interest	Overall findings
Shankar 2008[14]	Lombok, Nusa Tenggara Barat Province, Indonesia	Cluster RCT	2001-2004	Pregnant women of any gestational age	31290	Not clear but categorized as < 19, 20-34, ≥ 35	MMS during pregnancy and 90 days postpartum	30 mg iron and 400 µg FA along with 800 µg retinol, 200 IU vitamin D, 10 mg vitamin E, 70 mg ascorbic acid, 1.4 mg vitamin B1, 18 mg niacin, 1.9 mg vitamin B6, 2.6 µg vitamin B12, 15 mg zinc, 2 mg copper, 65 µg selenium, and 150 µg iodine	IFA only	Miscarriage (< 28 wks), stillbirth (≥ 28 wks), and fetal loss combining the two	MMS had no significant effects on miscarriage (*p* = 0.30), stillbirths (*p* = 0.26), or fetal loss (*p* = 0.14) compared to IFA
Zeng 2008[15]	Shaanxi Province, China	Cluster RCT	2002-2006	Pregnant women	5416	Around 25 (4)	MMS during pregnancy	Daily MMS of the UNIMMAP formulation	FA and IFA	Stillbirth (≥ 28 wks)	The risk of fetal loss did not differ across MMS, IFA, and FA arms
Roberfroid 2008[16]	Hounde Health District, Burkina Faso	RCT	2004-2006	Pregnant women	1260	Around 24.5 (6)	MMS during pregnancy and 3 mos after delivery	Daily MMS of the UNIMMAP formulation	IFA	Stillbirth (> 28 wks)	There was no difference in miscarriage between groups and a marginally greater risk of stillbirth (OR: 2.23; 95% CI: 0.97, 5.22; *p* = 0.06) in the intervention group
Sunawang 2009[17]	Indramayu, West Java, Indonesia	Cluster RCT	2001-2003	Pregnant women of 12-20 wks gestation	843	MMS group: 25.4 (6.1); IFA group: 26.1 (6.5)	MMS from 12-20 wks gestation and continued up to 30 days postpartum	Daily; contains the recommended daily allowance of 15 micronutrients according to the UNICEF/UNU/WHO recommended formula, including 30 mg of ferrous fumarate	IFA	Miscarriage (< 28 wks) and stillbirth (≥ 28 wks)	Miscarriage and stillbirth did not differ significantly between MMS and IFA arms
Bhutta 2009[18]	Bilal Colony, Karachi, Pakistan (urban); Kot Diji district, rural Sindh, Pakistan (rural)	Cluster RCT	2002-2004	Pregnant women < 16 wks gestation	2378	Urban: 25.3 (5.2); rural: 25.9 (5.3)	MMS during pregnancy	UNIMMAP formulation designed to provide 100% of the recommended dietary allowance of key vitamins and minerals, containing 30 mg of iron and 400 µg of FA along with 800 µg of retinol, 200 IU of vitamin D, 10 mg of vitamin E, 70 mg of ascorbic acid, 1.4 mg of vitamin B1, 18 mg of niacin, 1.4 mg of vitamin B2, 1.9 mg of vitamin B6, 2.6 µg of vitamin B12, 15 mg of zinc, 2 mg of copper, 65 µg of selenium	IFA only	Stillbirth (≥ 28 wks)	The rate of stillbirths was comparable in the two groups (RR: 0.87; 95% CI, 0.59, 1.28)
Aminisani 2009[19]	Ardabil Province, Iran	RCT	2004-2005	Pregnant women 16-20 wks gestation	179	Zinc group: 24.5 (4.8); placebo group: 23.9 (5.2)	Zinc Supplementation during the last two trimesters of pregnancy	50 mg daily elemental zinc	Placebo	Stillbirth	Of the 179 women who completed the follow-up until delivery, 4 (2 in the zinc-supplemented and 2 in the placebo group) delivered a

Study	Setting	Study design	Time period	Study population	Number of participants in analysis	Age[1]	Intervention	Frequency and dosage	Control	Outcomes of interest	Overall findings
											stillborn fetus (RR: 1.01; 95% CI: 0.15, 7.20)
Ramakrishnan 2010[20]	Cuernavaca, Mexico	RCT	2005-2007	Pregnant women with 18-22 wks gestation	973	18-35	DHA supplementation from 18-22 wks gestation through delivery	2 capsules daily (200 mg DHA in each capsule, 400 mg daily)	Placebo	Stillbirth (≥ 28 wks)	5 stillbirths were recorded (3 in control group and 2 in DHA group), with no significant differences between groups
Kawai 2010[21]	Dar es Salaam, Tanzania	RCT	2002-2004	HIV-infected pregnant women 12-27 wks gestation	1129	Multiple RDA multivitamins group: 26.3 (4.7); single RDA multivitamins group: 26.6 (4.9)	Either single or multiple RDA multivitamins from enrollment until 6 wks after delivery	Daily oral dose; the multiple RDA supplements contained doses at 3 times the RDA for vitamin E, 7 times the RDA for vitamin C, and > 10 times the RDA for several B vitamins	Single RDA multivitamin group	Miscarriage (< 28 wks), stillbirth (≥ 28 wks), and fetal loss combing the two	There was no difference in the risk of miscarriage (p = 0.17), stillbirths (p = 0.52), or fetal death (p = 0.99)
West 2011[22]	Northwestern Bangladesh	Cluster RCT	2001-2007	Pregnant women 13-45 yrs	59666	Range: 13-45	Vitamin A (retinol equivalents as retinyl palmitate or all-trans beta carotene from the first trimester through 12 wks postpartum	Weekly; 7000 ug retinol equivalents as retinyl palmitate or 42 mg of all-trans beta carotene	Placebo	Stillbirth (≥ 28 wks)	Weekly provision of one RDA of vitamin A or equivalent as beta carotene did not decrease the risk of stillbirth
Persson 2012[23]	Matlab, Bangladesh	RCT	2001-2009	Pregnant women < 14 wks gestation	2900	Around 26 (6)	MMS and food supplementation during pregnancy	Daily capsules of 30 mg of iron and 400 µg of FA, 60 mg of iron and 400 µg of FA, or MMS containing a daily allowance of 15 micronutrients, including 30 mg of iron and 400 µg of FA, was combined with food supplementation (608 kcal 6 days per week) randomized to either early invitation (9 wks gestation) or usual invitation (20 wks gestation)		Miscarriage (< 28 wks); sstillbirth (≥ 28 wks),	The risk of fetal loss including miscarriage and stillbirths did not significantly differ across groups
Vila-Nova 2013[24]	Bauru, Curitiba, Lajeado, Porto Alegre, Recife, and Salvador in Brazil	RCT	2001-2010	Women affected by non-syndromic cleft lip and cleft palate or have had a previous child with non-syndromic	268	16-45	FA supplementation from enrollment through the end of the first trimester	4.0 mg of FA daily	0.4 mg of FA daily	Miscarriage (≤ 20 wks)	Miscarriage rates were not significantly different between the two FA groups (p = 0.49)

Study	Setting	Study design	Time period	Study population	Number of participants in analysis	Age[1]	Intervention	Frequency and dosage	Control	Outcomes of interest	Overall findings
				orofacial clefts							
Wang 2013[25]	Henan, Guizhou, Hunan, and Jilin Provinces in China	Non-randomized intervention study	Not clear	Adult females not pregnant but planned to get pregnant in the next 6 mos	52043	Intervention group: 25.39 (3.94); control group: 24.87 (3.79)	MMS at least 3 mos before pregnancy throughout the first trimester	Each capsule contained 23 vitamins and micronutrients, including 400 µg FA, 563 IU vitamin A, 200 IU vitamin D2, 1.4 mg vitamin B1, 1.4 mg vitamin B2, 3 µg vitamin B12, 60 mg vitamin C, 8 mg vitamin E, 100 µg biotin, 14 mg niacinamide, 4 mg pantothenic acid, 100 mg calcium, 10 mg iron, 2 mg copper, 10 mg zinc, 77 mg phosphorus, 30 mg magnesium, 3 mg manganese, 30 µg selenium, 100 µg molybdenum, and 4 mg potassium. One capsule a day, after a meal	No intervention	Stillbirth	Periconceptional multiple micronutrient supplementation was associated with a lower incidence of stillbirth
Liu 2013[26]	Hebei Province, China	Cluster RCT	2006-2009	Pregnant women no more than 20 wks gestation	17897	23.7 (2.9)	MMS during pregnancy	Daily MMS of the UNIMMAP formulation	FA and IFA	Stillbirth (≥ 28 wks)	The risk of stillbirth did not differ by supplement group
Kiondo 2014[27]	Kampala, Uganda	RCT	2011-2012	Pregnant women with 12-22 wks gestation	833	Range: 15-42	Vitamin C supplementation from the second trimester of pregnancy until delivery	1000 mg vitamin C daily	Placebo	Stillbirth (> 24 wks)	High-dose vitamin C supplementation did not reduce the risk of stillbirth in low-risk women with poor nutritional status. (RR: 1.01, 95% CI: 0.54-1.87)
West 2014[28]	Gaibandha and Rangpur, Bangladesh	Cluster RCT	2007-2012	Pregnant women	44567	Not clear but categorized as < 20, 20-29, ≥ 30	MMS from early pregnancy to 12 wks postpartum	Daily MMS containing vitamins A (770 µg retinol activity equivalents), D (5 µg, or 200 IU), E (15 mg), B1 (thiamine, 1.4mg), B2 (riboflavin, 1.4mg), B3 (niacin, 18mg), B6 (pyridoxine, 1.9 mg), B9 (FA, 600 µg), B12 (cyanocobalamin, 2.6 µg), and C (85 mg); iron (27 mg); zinc (12 mg); copper (1000 µg); selenium (60 µg); and iodine (220 µg)	IFA only	Stillbirth (≥ 24 wks)	There was a significant reduction in stillbirth (RR: 0.89; 95% CI, 0.81, 0.99; *p* = 0.02) in MMS group compared to IFA group
Aflatoonian 2014[29]	Yazd, Iran	RCT	2013-2014	Women with infertility who had	106	Total range: 20-40; vitamin D	Vitamin D supplementation	50000 IU vitamin D capsule weekly	No intervention	Chemical pregnancy and	There were no significant impacts of vitamin D supplementation on chemical

Study	Setting	Study design	Time period	Study population	Number of participants in analysis	Age[1]	Intervention	Frequency and dosage	Control	Outcomes of interest	Overall findings
				undergone IVF/ICSI with cryopreservation of embryos; all had insufficient serum vitamin D (< 30 ng/ml)		group: 28.45 (3.74); control group: 29.56 (4.68)	for 6-8 wks during preconception			clinical pregnancy	(*p* = 1.00) or clinical (*p* = 0.81) pregnancy
Hekmatdoost 2015[30]	Tehran, Iran	RCT	2011-2014	Women with 3 or more idiopathic recurrent miscarriage	135	20-45	FA supplementation or MTHF supplementation from at least 8 wks before conception to the 20th wk of the pregnancy	1 mg of either FA or 5-MTHF daily	FA and MTHF served as each other's control	Ongoing pregnancy at the 20th wk	There was no significant difference in pregnancy rate between two groups
Becker 2015[31]	Rio Grande do Sul, Brazil	RCT	2012-2013	Women with infertility	26	18-35	Hypocaloric diet with a low glycemic index (GI < 55) and low glycemic load (daily value < 80) during preconception for 12 wks	The calorie content of the diet was individualized; each patient received an equivalent of 20 kcal/kg current body weight	Usual diet	Number of oocytes; clinical pregnancy rate; live birth rates	The low GI diet group had 85.4% more oocytes retrieved than the control group (*p* = 0.039). There was a moderate negative correlation between the number of oocytes retrieved with BMI (r^2 = - 0.542, *p* = 0.020), percentage of body fat (r^2 = - 0.475, *p* = 0.040), and leptin concentration (r^2 = - 0.515, *p* = 0.024). The clinical pregnancy rate was 21.4% in the low GI diet group because 3 of 14 patients experienced a spontaneous pregnancy during the follow-up; there were no pregnancies in the control group
Anees 2015[32]	Skardu, Muzaffarabad, and Rawalpindi in Pakistan	Non-randomized intervention study	Not clear	Pregnant women of lower socioeconomic status	460	Mean ± SEM around 26 ± 1.5 yrs	Iodine supplementation from first trimester	A single dose of 2 capsules of iodized oil (400 mg of iodine) taken orally in the first trimester from wks 6-8 of pregnancy	Subjects in non-endemic areas of Rawalpindi and clinically in euthyroid status and who had no previous known thyroid disorders served as a control for the study. Subjects of the goiter-endemic areas of Muzaffarabad and	Miscarriage; stillbirths	In the non-endemic area group, only one stillbirth was recorded. In the untreated endemic area group, two miscarriages and three stillbirths were reported. In the iodized oil administered endemic areas group, no miscarriage and no stillbirth was reported

Study	Setting	Study design	Time period	Study population	Number of participants in analysis	Age[1]	Intervention	Frequency and dosage	Control	Outcomes of interest	Overall findings
									Skardu with and without thyroid enlargement served as a non-supplemented control group for endemic areas		
Ashorn 2015[33]	Mangochi, Malawi	RCT	2011-2013	Pregnant women < 20 wks gestation	1307	25 (6)	MMS and LNS during pregnancy	Daily MMS containing twice the dosage of the UNIMMAP formulation for many of the micronutrients	IFA	Miscarriage (< 22 wks) and stillbirth (≥ 22 wks)	There were no significant differences in miscarriage between groups. There was a significant difference in stillbirths across groups (p = 0.006)
Adu-Afarwuah 2015[34]	Somanya-Kpong, Ghana	RCT	2009-2011	Pregnant women < 20 wks gestation	1057	IFA group: 26.5 (5.3); MMS group: 26.9 (5.7); LNS group: 26.5 (5.4)	MMS and LNS during pregnancy	Daily MMS containing twice the dosage of the UNIMMAP formulation for many of the micronutrients	IFA	Miscarriage (< 28 wks) and stillbirth (≥ 28 wks)	There were no significant differences between groups in miscarriages or stillbirths
Zahran 2016[35]	Assiut, Egypt	Non-randomized intervention study	2013-2014	Women with recurrent early pregnancy loss	300	Intervention group: 27.91 (5.68); Range: 20-40 Control group: 26.16 (5.05); Range: 19-42	Phase 1: FA, doxycycline hyclate; Phase 2: low-dose aspirin, progesterone during preconception and pregnancy	Folic acid: 5 mg once daily Doxycycline HCl: 100 mg twice daily Low-dose aspirin: 75 mg daily Progesterone: 200 mg daily	Some received folic acid plus low-dose aspirin in the first 3 mos or beyond this period with or without oral or vaginal progesterone. None received the pre-pregnancy regimen	Live birth, miscarriage, IUFD	There was a significant reduction in miscarriage (23.33% vs. 40.00%; p < 0.04) in the intervention group compared to the control group
Sayyah-Melli 2016[36]	Tabriz, Iran	RCT	2008-2013	Healthy nulliparous pregnant women with singleton pregnancy	410	20-30	FA supplementation from early pregnancy until delivery	5 mg FA daily	0.5 mg FA daily	Early miscarriage and late miscarriage	Early miscarriage was significantly higher among those receiving 0.5 mg FA than those receiving 5 mg FA (RR: 0.95; 95% CI: 0.92, 0.98; p = 0.001)
Zahiri Sorouri 2016[37]	Rasht, Iran	RCT	2010-2012	Healthy women at 16 wks gestation	540	Zinc group: 27.89 (4.17); control group: 28.44 (4.34)	Zinc supplementation from the 16th wk of gestation until delivery	400 μg FA and 30 mg iron (ferrous sulfate), with 15-mg zinc daily	400 μg FA and 30 mg iron (ferrous sulfate) without zinc supplementation	Miscarriage	There was no significant difference in miscarriage between the two groups (p = 0.772)
Al-Eisa 2017[38]	Mansoura, Egypt	Non-randomized intervention study	2013-2014	Women with obesity, with or without PCOS	90	20-35	Supervised aerobic training during preconception	Treadmill walking of 45 minutes 3 times per wk for 12 wks	Control Group: 1) had a BMI of 20-29; 2) healthy women tuba or unexplained infertility; 3) regular ovulatory cycles (25-35 days); 4) no endocrine abnormalities; 5)	FSH; AFC; ovarian volume	Weight loss from the intervention was significantly correlated to the improvement in reproductive function (p < 0.05)

Study	Setting	Study design	Time period	Study population	Number of participants in analysis	Age[1]	Intervention	Frequency and dosage	Control	Outcomes of interest	Overall findings
									normal ultrasonic ovarian morphology		
Al-Alousi 2018[39]	Najaf Al-Ashraf, Iraq	RCT	2017-2018	Women with subfertility	115	20-39	EPA and DHA during preconception for 8 wks	A 1000 mg capsule every day containing 180 mg EPA and 120 mg DHA	Placebo	Duration of infertility; number of follicles; number of retrieved oocytes; fertilization rate; number of metaphase II oocytes; number of injected oocytes; number of 2PM zygote; cleavage rate; total embryo; number of embryo in grades 1, 2, and 3; number of embryo transfer; endometrial thickness; AFC	The ratio of follicle/retrieved oocyte, the number of metaphase II oocytes, fertilization rate, and grade I embryo were higher in the group that received EPA/DHA than in the placebo group. Fertilization rate was higher in EPA/DHA group in comparison with the placebo group ($p < 0.05$)
Charkamyani 2019[40]	Tehran, Iran	Non-randomized intervention study	2017-2018	Women treated with IVF	170	19-45	Exercise training intervention program from 16-20 wks gestation and continuing for 90 days	3 exercise weekly classes for 60 minutes (10 min walking, 30 min aerobic exercise, 10 min strength-conditioning exercise, 10 min relaxation exercise)	No intervention	Miscarriage; infertility duration; fetal death	No significant difference was observed between control and intervention groups in any of the outcomes
Kadoura 2019[41]	Damascus, Syria	RCT	2016-2017	Women with PCOS	34	18-30	Metformin, calcium carbonate, and vitamin D3 (cholecalciferol), all given orally for 8 wks during preconception	The metformin dose was increased stepwise (starting with 500 mg once daily for the 1st wk and 500 mg twice daily in the second wk, followed by 500 mg 3 times daily from the 3rd wk onward). The dose of calcium carbonate (1000 mg/daily) and vitamin D3 (6000 IU/daily) remained constant throughout the study period	Metformin and placebo	LH; FSH; LH/FSH; improvement in menstrual irregularity	Calcium and vitamin D supplements could support metformin effect on regulation of menstrual cycle irregularity in vitamin D-deficient/insufficient PCOS patients, but this effect was not associated with any significant changes in gonadotropins (serum levels of LH, FSH, and LH/FSH)

Study	Setting	Study design	Time period	Study population	Number of participants in analysis	Age[1]	Intervention	Frequency and dosage	Control	Outcomes of interest	Overall findings
de Araújo, 2020[42]	Northeastern Brazil	RCT	2014-2017	Pregnant women with singleton pregnancy and 12-20 wks gestation and had at least one risk factor or adverse perinatal outcomes	829	18-45	Magnesium citrate supplementation from 12 to 20 wks gestation until delivery	300 mg/d elemental magnesium citrate	Placebo	Stillbirth (> 20 wks)	Oral magnesium supplementation did not reduce adverse perinatal or maternal outcomes in high-risk singleton pregnancies
Al-Bayyari 2021[43]	Irbid, Jordan	RCT	Started in 2014	Reproductive-age women with overweight and PCOD	58	23.7 (5.2)	Vitamin D3 supplementation for 12 wks	50,000 IU of vitamin D3 or placebo per wk (1 tablet per wk taken at home)	Placebo	Ovarian volume; follicle number; regularity of menstrual cycle	In the vitamin D group, significant changes were observed in ovarian volume, follicle numbers, and the regularity of the menstrual cycle ($p < 0.001$). In the placebo group, no significant changes in these outcomes were observed
Nausheen 2021[44]	Karachi, Pakistan	RCT	2013-2015	Pregnant women < 16 wks gestation with singleton pregnancy	257	26.03 (4.3)	Vitamin D3 supplementation from between 12-16 wks gestation to delivery	4000 IU Vitamin D/day (group A); 2000 IU Vitamin D/day (group B)	400 IU Vitamin D/day (group C)	Stillbirth	There were 2 stillbirths, both in the control group C (400/IU/day of vitamin D3 supplementation).
Sudfeld 2022[45]	Dar es Salaam, Tanzania	RCT	2015-2019	HIV-infected pregnant women 12-27 wks gestation	2097	Adult; not further defined but categorized as 18-24, 25-34, and 35+	Vitamin D3 supplementation from the second trimester of pregnancy (12-27 wks) until 1 yr postpartum.	Daily 3,000 IU vitamin D3 oral supplements (cholecalciferol)	Placebo	Fetal death, miscarriage (< 28 wks), and stillbirth (≥ 28 wks)	There was no difference in the risk of fetal death, miscarriage, or stillbirth between the vitamin D3 and placebo groups
de Kok 2022[46]	Hauts-Bassins region, Burkina Faso	RCT	2019-2021	Pregnant women with < 21 wks gestation	1708	15-40	Fortified BEP + IFA during pregnancy	Daily, 72 g fortified BEP (an LNS in the form of an energy-dense peanut paste fortified with multiple micronutrients)	IFA only (65 mg iron and 400 ug FA daily)	Fetal death (< 22 wks); fetal death ≥ 22 wks and < 28 wks); stillbirth (≥ 28 wks)	There were no significant differences (all $p > 0.1$) in fetal loss or stillbirth between groups
Ilboudo 2022[47]	Sindou, Burkina Faso	Cluster RCT	2015-2016	Pregnant women in the first or second trimester of pregnancy	617	Not clear but categorized as < 18 for adolescents and ≥ 18 for adults	Home-based visits focused on nutritional counseling, including management of anemia in pregnancy and	Monthly intervention	The usual package of activities of the national program without home visits	Miscarriage (< 22 wks) and stillbirth (≥ 22 wks)	Personalized home-based support for pregnant women, focusing on nutritional counseling, could reduce stillbirths but not miscarriage

Study	Setting	Study design	Time period	Study population	Number of particip ants in analysis	Age[1]	Intervention	Frequency and dosage	Control	Outcomes of interest	Overall findings
							training on nutrition				

AFC, antral follicle count; BEP, balanced energy and protein; BMI, body mass index; CI, confidence interval; DHA, docosahexaenoic acid; EPA, eicosapentaenoic acid; FA, folic acid; FSH, follicle-stimulating hormone; GI, glycemic index; HIV, human immunodeficiency virus; ICSI, intracytoplasmic sperm injection; IDA, iron deficiency anemia; IFA, iron and folic acid; IVF, in vitro fertilization; LH, luteinizing hormone; LNS, lipid-based nutrient supplement; MMS, multiple micronutrient supplementation; MTHF, methyltetrahydrofolate; OR, odds ratio; PCOS, polycystic ovary syndrome; Q1, 25th percentile; Q3, 75th percentile; UNIMMAP, United Nations International Multiple Micronutrient Preparation; RDA, recommended dietary allowance; RCT, randomized controlled trial; RR, risk ratio; SEM, standard error of the mean.

[1] The values are range or means (standard deviations) unless stated otherwise. The units for age are years unless stated otherwise.

[2] Randomized controlled trials with individual randomization unless indicated otherwise.

Supplemental Table 6. Intervention-outcome combinations examined in the intervention studies

Outcome	Intervention	Number of Studies (number of studies with quantitative estimates for meta-analysis)
Miscarriage	MMS	9 (8)
	Folic acid	4 (2)
	Zinc	3 (2)
	Vitamin D	1
	Vitamin A	2
	Iron	1
	Iodine	1
	Nutrition counseling and education	1
	Physical activity intervention	1
	Lipid-based nutrient supplement	1
Stillbirth	MMS	17 (15)
	Zinc	2
	Vitamin D	2
	BEP	2
	Vitamin A	3
	Folic acid	2
	DHA	1
	Vitamin C	1
	Magnesium	1
	Iron	2
	Iodine	1
	Nutrition counseling and education	1
	Lipid-based nutrient supplement	1
Composite outcome of fetal death (including stillbirth and miscarriage)	MMS	4
	Vitamin A	2
	Zinc	2
	Selenium	1
	Vitamin D	1
	BEP	1
	Folic acid	1
	Iron	1
	Physical activity intervention	1
Ovulation rate	Weight reduction diet	1
Pregnancy rate	Weight reduction diet	1
	Vitamin D	1
	Low glycemic index diet	1
Number of oocytes	Low glycemic index diet	1
	EPA/DHA	1
Number of follicles	EPA/DHA	1
	Vitamin D	1
Live birth rate	Low glycemic index diet	1
	Folic acid	1
Duration of infertility	EPA/DHA	1
	Physical activity intervention	1
Fertilization rate	EPA/DHA	1
Number of metaphase II oocytes	EPA/DHA	1
Number of injected oocytes	EPA/DHA	1
Cleavage rate	EPA/DHA	1
Total embryo	EPA/DHA	1
Number of embryos in grades 1, 2, and 3	EPA/DHA	1
Number of embryo transfer	EPA/DHA	1
Endometrial thickness	EPA/DHA	1
Antral follicle count	EPA/DHA	1
	Physical activity intervention	1
LH	Calcium and vitamin D	1
FSH	Calcium and vitamin D	1
	Physical activity intervention	1
LH/FSH	Calcium and vitamin D	1
Menstrual (ir)regularity	Calcium and vitamin D	1
	Vitamin D	1
Ovarian volume	Vitamin D	1
	Physical activity intervention	1
Age at menarche	High energy and high protein supplement	1

BEP, balanced energy and protein; DHA, docosahexaenoic acid; EPA, eicosapentaenoic acid; FSH, follicle-stimulating hormone; LH, luteinizing hormone; MMS, multiple micronutrient supplementation.

Supplemental Table 7. Characteristics of the included observational cohort studies

Study	Setting	Time period	Study population	Number of participants in analysis	Age[1]	Exposure	Outcomes of interest	Overall findings
Pharoah 1976[48]	New Guinea	1966-1974	Pregnant women	106	Range: 20-50	Iodine deficiency during pregnancy	Stillbirth	More stillbirths and infant deaths occurred among the offspring of women who showed biochemical evidence of iodine deficiency
Popkin 1993[49]	Cebu, Philippines	1983-1984	Participants from CLHNS, a community-based, prospective study of more than 3,000 women from urban and rural communities in the Metro Cebu area	2434	Not clear	BMI and maternal fat intake during postpartum period	Time to menses; Time from menses to conception	Low BMI and lower dietary fat intake were associated with a greater duration of postpartum amenorrhea
Kurz 1993[50]	Guatemala	1973-1975	Mother-infant pairs from the INCAP study in four villages	282	Range: 14-47; mean (SD): 27.9 (7.0)	Nutritional status during pregnancy	Length of postpartum amenorrhea	Greater maternal triceps skinfold thickness at 3 mo postpartum was associated with a higher risk of the mother resuming menstruation (OR: 1.64; 95% CI: 1.02-2.65). Maternal triceps skinfold thickness, arm circumference, thigh skinfold thickness, and maternal BMI were all predictors of length of amenorrhea (at $p < 0.1$ level)
Khan 1996[51]	Rural Guatemala	1969-1992	Female children of participants of the INCAP study in four villages	250	Not clear	Stunting at 3 yrs of age, defined using HAZ and classified as none (> -2.0 SD), moderate (-2.0 SD to -3.0 SD), and severe (< -3.0 SD); energy intake (kcal/d) during childhood	Age at menarche	Women with severe stunting during early childhood reached menarche about 6 mos later than those with less stunting. Greater energy intake from home diet was associated with earlier menarche
Onadeko 1996[52]	Ibadan, Nigeria	1985-1986	Pregnant women	4649	Not clear	Hb concentration during pregnancy	Stillbirth	Maternal Hb concentration during pregnancy was not significantly associated with stillbirth
Simondon 1998[53]	Dakar, Senegal	Not clear	Girls born in the area between January 1978 and October 1984 who were still alive and had not emigrated in February 199	1650	Range: 12-17	Preschool stunting	Age at menarche	Girls with stunting experienced a significant delay in age at menarche of 1.6 yrs compared to the tallest girls
Agarwal 1998[54]	Varanasi, India	1988-1992	Pregnant women	8111	Range: 30.4-43.5	Mid-arm circumference; Hb concentration; dietary pattern during pregnancy	Stillbirth (≥ 28 wks); miscarriage	The stillbirths showed significant relationship with maternal weight, height, and dietary protein intake ($p < 0.001$ for all). Greater mid-arm circumference and greater Hb concentration were also associated with a lower risk of miscarriage
Ramakrishnan 1999[55]	Guatemala	1966-1994	All women of reproductive age who were residents of the four INCAP study villages	240	Mean age at follow-up: 23.47; range: 19.22-28.76	Dietary supplement (Atole or Fresco), and stunting	Age at menarche; first pregnancy; first birth	The median time intervals from menarche to first intercourse and from first intercourse to first birth were 1.68 and 0.06 yrs shorter among those who received Atole than those who received Fresco. All fertility outcomes were significantly delayed in the Fresco village. Median age at first birth was 1.04 yrs earlier among those without severe stunting than those with severe

Study	Setting	Time period	Study population	Number of participants in analysis	Age[1]	Exposure	Outcomes of interest	Overall findings
								stunting. These differences, however, were not significant (*p* = 0.25)
Adair 2001[56]	Metro Cebu, Philippines	1983-1999	Girls born from the CLHNS study	966	Girls were measured immediately after birth, bimonthly until 2 yrs of age, and during follow-up surveys at ages 8 to 9, 11 to 12, and 14 to 15 yrs	Weight, length, early postnatal growth rates, premenarcheal body composition, current diet, maternal characteristics while pregnant with the study child	Age at menarche	Girls born long and light at birth had the highest hazard (HR: 1.61; 95% CI: 1.27-2.04) and the earliest age at menarche
Gindler 2001[57]	Jiaxing City, China	1993-1995	Women who became pregnant for the first time	23806	FA group: 23.5 (1.5); no FA pills group: 23.8 (2.1)	Use of FA pills before or during pregnancy	Miscarriage (< 20 wks)	The rates of miscarriage were similar for women with no folic acid use and those with any pattern of folic acid use during preconception or pregnancy
Khan 2001[58]	Abbottabad, India	1992-1993	Pregnant women	73	Not clear	Anemia during pregnancy	Fetal deaths including stillbirths	Mothers with nutritional or iron deficiency anemia had higher rates of stillbirths than mothers without anemia
Osman 2001[59]	Maputo, Mozambique	Not clear	Pregnant women ≤ 21 wks gestation	Not clear; out of a total of 908 enrolled, 8% were lost to follow-up at birth, and 9.6% did not have birthweight data thus the sample size might be 748	Not clear	Weight, weight gain, height, MUAC, BMI during pregnancy	IUFD	Low weight, height, MUAC, and BMI were not significantly associated with IUFD. Low weight gain was associated with greater odds of IUFD
Chalumeau 2002[60]	Ivory Coast, Mali, Senegal, Niger, Mauritania, Burkina Faso	1994-1996	Singleton pregnancies	19809	Not clear but categorized into 2 groups (≥ 35 and < 35)	Maternal height and iron supplementation during pregnancy	Stillbirths (> 22 wks)	Short maternal stature (< 150 cm) was a risk factor for late stillbirth (OR: 2.3; 95% CI: 1.2, 4.2; *p* = 0.007). No significant association was found with use of iron supplementation (*p* = 0.15)
Villamor 2004[61]	Dar es Salaam, Tanzania	Not clear	HIV-positive pregnant women 12-27 wks gestation	1002	Range: 15-45; mean (SD): 24.6 (4.8)	GWG during pregnancy	Fetal death, including miscarriages (< 28 wks) and stillbirths (≥ 28 wks)	Weight loss during pregnancy was associated with a greater risk of fetal death (RR: 1.83; 95% CI: 0.93, 3.57), with the association greater for weight loss during the second trimester. Similar but weaker associations were found with low weight gain during pregnancy
Lone 2004[62]	Karachi, Pakistan	2001-2002	Pregnant women with < 16 wks gestation and singleton pregnancy	629	Anemia group: 26.85 (4.77); non-anemia group: 27.08 (4.65)	Anemia during pregnancy	Stillbirths (> 24 wks)	The risk of IUFD was 3.7 times higher among women with anemia, but the association was not significant
Kupka 2005[63]	Dar es Salaam, Tanzania	1995-1997	HIV-positive pregnant women 12-27 wks gestation	670	Around 25 (5)	Plasma selenium status during pregnancy	Fetal death, including stillbirth (≥ 28 wks) and miscarriage (< 28 wks)	Risk of fetal death was significantly elevated in the lowest tertile of plasma selenium (RR: 1.94; 95% CI: 1.08, 3.49) relative to the highest tertile
Watson-Jones 2007[64]	Mwanza, Tanzania	1997-2000	Pregnant women with singleton pregnancy	1536	Mean: 23.8	Short stature (height < 156 cm) and anemia during pregnancy	IUFD (≤ 22 wks) and stillbirths (> 22 wks)	Stillbirth was associated with short stature (OR: 2.64; 95% CI: 1.1, 6.3; *p* = 0.03) and maternal anemia (OR: 3.74; 95% CI: 1.1, 12.8; *p* = 0.02)

Study	Setting	Time period	Study population	Number of participants in analysis	Age[1]	Exposure	Outcomes of interest	Overall findings
Ronnenberg 2007[65]	Anhui, China	1996-1998	Female textile workers who conceived at least once during prospective observation between 1996 and 1998	364	24.9 (1.5)	Vitamin B6 and homocysteine status during preconception	Conception; clinical pregnancy; early pregnancy loss	Women with sufficient vitamin B6 had a higher hazard of conception (HR: 1.4, 95% CI: 1.1, 1.9) and lower odds of early pregnancy loss in conception cycles (OR: 0.7; 95% CI: 0.4, 1.1) than did women with vitamin B6 deficiency
Prabhakar 2007[66]	Chandigarh, India	Not clear	Reproductive age group women with generalized epilepsy	25	Range: 15-28; mean (SD): 18.3 (3.7)	BMI during preconception	Amenorrhea, oligomenorrhea, and irregular menstrual cycles	There was a significant positive correlation existed between weight gain and the development of menstrual abnormalities (r = 0.66, $p < 0.0001$)
Bosch 2008[67]	Matlab, Bangladesh	1988-2001	Follow-up of under-five children who were enrolled in an earlier study on childhood infectious diseases conducted in 1988-1989	255	Age in early childhood: 19.5 (13.6) mos; age in adolescence: 14.0 (1.3) yrs	Nutritional status during early childhood and adolescence	Age at menarche	Girls with stunting had a higher age at menarche
Mehta 2008[68]	Blantyre, Malawi; Lilongwe, Malawi; Dar es Salaam, Tanzania; Lusaka, Zambia	2001-2003	HIV-infected women	2126	25.3 (0.10)	BMI, Hb concentration, and GWG during pregnancy	Composite outcome of fetal loss	Severe anemia (Hb < 8.5 g/dL) during preconception was associated with greater odds of stillbirth (OR: 3.67; 95% CI: 1.16, 11.66) than those with Hb ≥ 11 g/dL. A 1-g/dL decrease in Hb during preconception was associated with greater odds of stillbirth (OR: 1.5; 95% CI: 1.19, 1.89; $p = 0.01$). A one kilogram-per-wk decrease in weight change was associated with lower odds of fetal loss or stillbirth (OR: 0.28; 95% CI: 0.09, 0.91; $p = 0.03$). BMI was not a significant predictor of stillbirth
Hauger 2008[69]	Buenos Aires, Argentina	2003-2006	Women with pregnancies ending in a live birth or fetal death with at least 22 wks gestation or birth weight higher than 500 g	46964	Not clear but categorized into 3 groups (≤ 19, 20-34, ≥ 35)	BMI during preconception	Stillbirths (> 22 wks)	There was no association between maternal weight status and the risk of stillbirth
Zhang 2009[70]	Zhejiang, China; Jiangsu, China	1993-1996	Women in a population-based pregnancy-monitoring system	164667	Not clear but categorized as < 25, 25-29, 30-34, and ≥ 35	Anemia during pregnancy	Stillbirth (≥ 20 wks)	Women with anemia during the third trimester had a lower risk of stillbirth. In the third trimester, Hb concentrations between 9 and 10 g/dL were associated with a 20% lower risk for stillbirth, while Hb of 12 g/dL was associated with a slightly higher risk for stillbirth (HR: 1.1; 95% CI: 1.0, 1.2)
Chumak 2010[71]	Monchegorsk, Russia	1973-2002	Women who gave birth and had data on anemia in pregnancy in Monchegorsk between 1973 and 2002	24525	Range: 13-46	Anemia during pregnancy	Stillbirth	Women with anemia in pregnancy were less likely to have stillbirths (OR: 0.68; 95% CI 0.52, 0.89)
Mesa 2010[72]	Pelotas, Southern Brazil	1982-2005	Women from the Pelotas Birth Cohort Study	2083	In 2004-2005, the participants were 23-24	Growth patterns during childhood, including WAZ, HAZ, and WHZ	Age at menarche	Higher WAZ, HAZ, and WHZ at 19.4 and 43.1 mos were associated with a higher prevalence of the onset of menarche before age 12
Guerra-Shinohara 2010[73]	Sao Paulo, Brazil	2004-2005	Healthy pregnant women of 4-16 wks gestation	100	Among those without miscarriage:	BMI, alcohol intake, use of multivitamins, vitamin status, and vitamin-	Miscarriage	The risk of miscarriage was significantly associated with MMA (OR: 3.80; 95% CI: 1.36, 10.62; per quartile increase in MMA) and BMI (OR: 5.49; 95% CI:

Study	Setting	Time period	Study population	Number of participants in analysis	Age[1]	Exposure	Outcomes of interest	Overall findings
					median (Q1, Q3): 25.5 (21.3, 30.0) Among those with miscarriage: median (Q1, Q3): 28.0 (24.3, 34.5)	dependent metabolites during early pregnancy		1.29, 23.39; per quartile increase in BMI)
Zhang 2010[74]	Hangzhou, China	2002-2008	Women with infertility who underwent their first IVF or ICSI	2628	Around 31 (4)	BMI during preconception	Pregnancy rate, early pregnancy loss (biochemical pregnancy without ultrasound signs of viable pregnancy), miscarriage (< 12 wks), live-birth rate, and fertilization rate	Women with obesity exhibited fewer oocytes retrieved and lower fertilization rate (*p* < 0.001) than women with normal weight. Compared with women with normal weight, women with overweight displayed fewer oocytes retrieved (*p* < 0.001) and lower fertilization rate (*p* < 0.001). Pregnancy rate, miscarriage, and live birth rate were comparable among the three BMI groups
Li 2010[75]	China	2004-2006	Patients undergoing IVF or ICSI from January 2004 to December 2006	1107	Around 31 (4)	BMI during preconception	Early pregnancy loss, implantation, and miscarriage (< 28 wks)	Biochemical pregnancy, clinical pregnancy, implantation, and live birth rates show no differences among three BMI groups
Abeysena 2010[76]	Sri Lanka	2001-2002	Pregnant women < 16 wks gestation	766 for the analysis on miscarriages	26.4 (5.5)	Anemia during pregnancy at or before 16 wks gestation	Miscarriage (< 28 wks)	There was no association between anemia (< 11 g/dL) and miscarriage (OR: 1.30; 95% CI: 0.39, 4.41; *p* = 0.67)
Villamor 2011[77]	Bogotá, Colombia	2006-2008	Children enrolled in public primary schools	242	8.8 (1.6)	Vitamin D status during childhood and adolescence	Age at menarche	Vitamin D status was inversely associated with the probability of having menarche. Vitamin D deficiency is associated with earlier menarche
Belachew 2011[78]	Multiple settings in Ethiopia	2005-2010	Adolescents	900	Around 15 (1.3)	Stunting and food insecurity during adolescence	Age at menarche	Adolescents with food insecurity had menarche one yr later than those who were food secure. The hazard of menarche showed a significant decline to 0.936 (95% CI: 0.756, 1.158) and 0.496 (95% CI: 0.276, 0.892), respectively, for mild food insecurity and moderate/severe food insecurity than girls with food security. Girls with stunting had their menarche one yr later than their peers without stunting (HR: 0.551, *p* < 0.001)
Stringer 2011[79]	Lusaka, Zambia	2006-2009	Pregnant women	100454	Median (Q1, Q3): 24 (21, 29)	BMI and Hb during pregnancy	Stillbirth	Baseline BMI greater than 26 (OR: 1.8; 95% CI: 1.4, 2.3) was associated with a greater risk of stillbirth
Chumak 2011[80]	Monchegorsk, Northwest Russia	1973-2002	Using the data from the Kola Birth Registry, women who gave birth during 1973-2002 were selected	24526	Not clear	Hb concentration and anemia during pregnancy	Stillbirth	No significant difference was observed for stillbirths between women with and without anemia defined using the cut-off of 110 g/L
Gonzales 2012[81]	Peru	2009-2010	Pregnant women with singleton pregnancies of 20-42 wks	295651	Not clear	Hb concentrations and anemia during pregnancy	Stillbirth (> 20 wks)	Risk for stillbirths was positively associated with the severity of low maternal Hb (OR: 1.39 for Hb 9-9.9;

Study	Setting	Time period	Study population	Number of participants in analysis	Age[1]	Exposure	Outcomes of interest	Overall findings
								OR: 1.84 for Hb 8-8.9; OR: 3.25 for Hb 7-7.9; and OR: 7.8 for Hb < 7 g/dL); and with Hb higher than 14.5 g/dL (OR: 1.31)
Reyes 2012[82]	Mexico	2007-2008	Women attending prenatal care with an uncomplicated pregnancy and ≤ 13 wks gestation	546	Normal weight group: 27.03 (8.5); overweight group: 30.5 (7.3); obesity group: 31.2 (6.1)	BMI during preconception	Miscarriage (< 20 wks) and stillbirth	Women with obesity had the highest risk of a lack of spontaneous labor ($p < 0.001$)
Young 2012[83]	Tororo, Uganda	2009-2011	HIV-infected pregnant women of 12-28 wks gestation	158	Median age (Q1, Q3): 29 (26, 34)	BMI, Hb concentrations, and GWG during pregnancy	Fetal death, including miscarriage (12-20 wks) and stillbirth (> 20 wks)	Gaining < 0.1 kg per wk was associated with greater odds of an adverse birth outcome (OR 2.85, 95% CI 1.32, 6.15, $p < 0.01$)
Neumann 2013[84]	Embu District of Eastern Province, Kenya	1984-1991	Pregnant women	138	Not clear	Vitamin B12 deficiency during pregnancy	Stillbirth	Women with vitamin B12 deficiency during pregnancy had more stillbirths than women who were not vitamin B12 deficient. Stillbirths were correlated with maternal macrocytic anemia
Malhotra 2013[85]	New Delhi, India	2007-2009	Women with infertility	183	Range: 20-42; mean (SD): 32.13 (4.04)	BMI during preconception	AFC, ovarian volume, FSH, LH	Women with overweight and obesity had a significantly low AFC ($p < 0.0129$) on the right side. There was no difference in ovarian volume in either of the groups. FSH and LH were comparable in the three BMI groups
Kumari 2013[86]	Jammu District, India	2006-(possibly) 2007	Pregnant women	271	Not clear but categorized into < 19, 20-29, 30-39, and > 40	Anemia during pregnancy	Pregnancy wastage including miscarriage and stillbirths	Women with anemia had a higher risk of miscarriage or stillbirth (OR: 6.49; 95% CI:1.48-39.94)
Sharma 2014[87]	Bangalore, India	2010-2011	Women in a fertility clinic	500	Range: 25-35	BMI during preconception	Clinical pregnancy; number of oocytes retrieved; mature oocytes; implantation rate; live birth rate; miscarriage	There were positive associations between higher BMl and unfavorable outcomes of IVF/ICSI treatment, including lower clinical pregnancy, higher early pregnancy loss, higher dosage and duration of gonadotropins requirement, risk of cancellation, and fewer collected oocytes. Implantation rate, pregnancy rate, and miscarriage were comparable across BMI categories, but live births are higher in normal weight and overweight than in the extremes of BMI
Huang 2014[88]	Wuhan, China	2009-2011	Patients with infertility due to PCOS or tubal factor who were undergoing IVF/ICSI treatment	256	Around 30 (4)	BMI during preconception	Number of oocytes retrieved; number of available embryos; clinical pregnancy rate;	Among women with PCOS, no significant association was observed between BMI and the number of oocytes retrieved or number of available embryos was observed. Patients with leanness had a higher clinical pregnancy rate ($p = 0.022$) than patients with overweight and obesity. Comparisons of

Study	Setting	Time period	Study population	Number of participants in analysis	Age[1]	Exposure	Outcomes of interest	Overall findings
							miscarriage; live birth rate	women with leanness versus overweight and obesity showed live birth rates of 40.5% versus 24.5% ($p > 0.05$) and miscarriage rates of 13.5% versus 7.7% ($p > 0.05$), respectively
Zhou 2014[89]	Guangzhou, China	2010-2011	Adult pregnant women	1963	Around 30 (4)	Vitamin D status during pregnancy	Miscarriage (< 20 wks) and IUFD (≥ 20 wks)	There was no significant association between vitamin D status and miscarriage or IUFD
Ouyang 2014[90]	Anhui, China	1996-1998	Newly married, nulligravid textile workers who intended to become pregnant	291	24.9 (1.5)	Vitamin B6 status, vitamin B12 status, and folate status during preconception	Clinical pregnancy; early pregnancy loss; miscarriage (≤ 20 wks)	Compared with women with adequate B-vitamins, rate of clinical pregnancy was lower in women with B-vitamin deficiency ($p < 0.05$ for all)
Cung 2014[91]	Nablus City, West Bank, Palestine	Started in 2010	Pregnant women identified from a delivery registry	5644	Range: 15-48; mean: 27	Hb concentrations during pregnancy	Stillbirth (≥ 28 wks)	The risk of a stillbirth was six times higher among women with high Hb, whereas women with anemia did not have a greater risk
Meng 2015[92]	Shanxi, China	2009-2012	Non-pregnant women who planned to become pregnant in the following 12 mos	1627	24.0 (3.6)	BMI during preconception	Infertility (impaired fecundity) rate	One 1-unit increase in BMI was associated with lower odds of pregnancy (adjusted fecundity ratio: 0.98; 95% CI: 0.96 to 0.99)
Zhai 2015[93]	Shenyang, China	1999-2003	Healthy girls from the second grade of elementary schools	120	Around 8.5 (0.3)	Body fat percentage by skinfold thickness	Age at menarche	By the fourth year, 50.0% of girls with obesity had menarche onset, which was higher than among girls with normal weight (27.5%) and leanness (8.1%)
Salgin 2015[94]	Johannesburg-Soweto, South Africa	1990	Children enrolled into the birth cohort through public antenatal and delivery clinics and hospitals	2352	Not clear	Weight gain during infancy	Age at menarche	Greater catch-up weight gain from birth to the age of 1 yr was associated with earlier menarche
Awan 2015[95]	Pakistan	2010-2012	Pregnant women with singleton pregnancy in the first trimester	200	Range: 30-45; mean (SD): 30 (4.1)	BMI during early pregnancy	Stillbirth	Stillbirth was more frequent in pregnant women with overweight than those with normal weight ($p = 0.0133$)
Shen 2015[96]	Shanghai, China	2013-2014	Pregnant women	1568	Range: 18-39; mean: 26.4	Serum copper, zinc, calcium, and iron concentrations during preconception and pregnancy	Miscarriage	Serum zinc and iron levels in patients with miscarriage were significantly lower than in patients who reached the third trimester of pregnancy
Jansen 2016[97]	Bogotá, Colombia	2006	Children from public primary schools	456	8.4 (1.7)	Red meat intake and tuna/sardine intake	Age at menarche	Red meat intake frequency was associated with an earlier age at menarche (p for trend = 0.0009). Intake of canned tuna/sardines was associated with a later age at menarche
Shen 2016[98]	China	2011-2013	Women with infertility and without PCOS undergoing their first IVF-ET treatment	411	Around 33 (4)	BMI during preconception	Embryo implantation rate; clinical pregnancy; miscarriage; live birth	Women with obesity had a longer infertility duration than women with normal weight ($p < 0.01$). Patients with overweight or obesity had less mature oocytes, fertilization rate, blast formation rate, and embryo implantation rate ($p < 0.05$). Clinical pregnancy rate and live birth rate also showed a decreasing trend in patients with overweight or obesity

Study	Setting	Time period	Study population	Number of participants in analysis	Age[1]	Exposure	Outcomes of interest	Overall findings
Zhou 2016[99]	Anhui, China	2013-2014	Women enrolled before pregnancy and follow-up continued until pregnancy outcomes occurred or for a maximum of 24 mos	2940	Range: 18-40	BMI during preconception	Miscarriage (< 20 wks)	Women with obesity, overweight, and underweight prior to pregnancy were about 2.01 (95% CI: 1.1,3.68), 1.71 (95% CI: 1.04,2.81), and 2.05 (95% CI: 1.3,3.23) times more likely, respectively, to have miscarriage, than women with normal pre-pregnancy BMI
Pan 2016[100]	220 counties from 31 mainland provinces of China	2010-2012	Married women enrolled in the National Free Preconception Health Examination Project and planned to become pregnant within the next 6 mos	536098	21-49	BMI during preconception	Miscarriage (< 28 wks) and stillbirth (≥ 28 wks)	Pre-pregnancy underweight was associated with miscarriage (OR 1.11; 95% CI 1.06 to 1.17). Women with obesity had a higher risk of spontaneous miscarriage (OR 1.13; 95% CI 1.02 to 1.26) and stillbirth (OR 1.59; 95% CI 1.18 to 2.15)
Zerfu 2016[101]	Arsi zone, Oromia, Ethiopia	Not clear	Pregnant women	374	Around 25 (0.3)	Dietary diversity during pregnancy	Stillbirth (> 24 wks)	No association was observed between the adequacy of dietary diversity during pregnancy and stillbirth (RR: 2.71; 95% CI: 0.88, 8.36)
Aydin 2017[102]	Istanbul, Turkey	Not clear	Healthy children < 9 yrs	157	7.6 (0.9)	Weight gain, height gain, and BMI during adolescence	LH, FSH, estradiol, ovarian volume	Girls with pubertal signs were heavier than their peers starting at 9 mos of age ($p = 0.02$), and the difference became more evident over time ($p < 0.001$). Accelerated weight gain between 6 and 15 mos of age was associated with greater odds of having a pubertal sign at the study visit (OR: 34.5, $p = 0.004$)
Ahmed 2017[103]	Sulaimani Governorate, Kurdistan Region, Iraq	2012-2016	Female patients with obesity who underwent sleeve gastrectomy	16	Range: 20-35	Weight loss after gastrectomy surgery	Pregnancy and ovulation	Significant changes in fertility were recorded in females with PCOS and obesity after bariatric surgery as early as 3 mos after the surgery
Villamor 2017[104]	Bogotá, Colombia	2006-2015	A random sample of children aged from the public primary school system	1464	Range: 5-12; mean (SD): 8.6 (1.7)	Status of circulating Hb, ferritin, mean corpuscular volume, zinc, vitamin B12, erythrocyte folate, and retinol during childhood	Age at menarche	Higher iron status in middle childhood is related to later age at menarche whereas Hb concentrations are inversely associated with age at onset of menses. Every 1 SD higher Hb (11 g/L) was related to a 7% (95% CI: 2, 13; $p = 0.01$) higher probability of experiencing menarche at any given time of follow-up. Plasma ferritin was positively related to age at menarche. The probability of having menarche was 8% lower (95% CI: 3, 13; $p = 0.002$) for every 1 SD higher ferritin
Narasati 2017[105]	Jakarta, Indonesia	2012-2016	Couples undergoing IVF cycles	1062	≤ 49	BMI during preconception	Clinical pregnancy	Underweight, overweight, class I obesity, and class II obesity had no significant association with clinical pregnancy rate than normal weight
MacKenna 2017[106]	15 Latin American countries	2010-2014	Patients admitted for IVF and ICSI	107313	36.4 (4.6)	BMI during preconception	Number of oocytes retrieved; pregnancy; live birth; miscarriage	Overweight and obesity were associated with a lower mean number of oocytes retrieved, but were not significantly associated with pregnancy, live birth, or miscarriage

Study	Setting	Time period	Study population	Number of participants in analysis	Age[1]	Exposure	Outcomes of interest	Overall findings
Kumar 2017[107]	Tirupati, India	Not clear	Pregnant women	112	Range: 18-30; mean (SD): 22.25 (3)	Hb status during pregnancy	Miscarriage	Miscarriage was significantly related to the Hb status in early pregnancy than to the Hb status in late pregnancy
Suryanarayana 2017[108]	Karnataka, India	2013-2015	Pregnant women	427	Mean: 22.4	Anemia during pregnancy	Miscarriage or stillbirth	Anemia during pregnancy was associated with greater maternal and fetal risks
Sheng 2017[109]	China	2011-2013	Women with PCOS undergoing IVF/ICSI	774	Range: 20-35; mean (SD): 27.9 (3.1)	BMI during preconception	Biochemical pregnancy; clinical pregnancy; ongoing pregnancy; implantation rate; miscarriage (≤ 12 wks)	The implantation rate was lower in women with obesity than in women with normal weight. Clinical pregnancy rates differed among BMI categories (p = 0.033), but there was no difference for biochemical (p = 0.327) and ongoing (p = 0.084) pregnancy rates. The miscarriage rate was similar among BMI categories
Cai 2017[110]	Xiamen, China	2013-2014	Patients who underwent IVF/ICSI treatment and fresh autologous embryo transfer	4401	Range: 20-46; mean (SD): 31.35 (4.29)	BMI during preconception	Number of oocytes retrieved; fertilization rate; clinical pregnancy; live birth; miscarriage (< 22 wks)	Pregnancy rates, miscarriage rates per pregnancy, and live birth rates were similar among BMI categories. Underweight was associated with a lower live birth rate (OR: 0.80; 95% CI:0.68, 0.94), whereas the association between overweight and live birth rates was not significant. Underweight was associated with a higher risk of miscarriage 1 (OR: 1.51; 95% CI 1.13, 2.07) than normal weight, and overweight had no significant effect on miscarriage rates. More oocytes were retrieved in the group with underweight than in the groups with normal weight (p = 0.043) and overweight (p < 0.001)
Mosha 2017[111]	Dar es Salaam, Tanzania	2001-2004	HIV-negative pregnant women of 12-27 wks gestation	7634	Mean (SD; range): 25.2 (5.1; 13.5-45.5)	Dietary iron and calcium intake during pregnancy	Stillbirth (≥ 28 wks)	Dietary iron intake was associated with a lower risk of stillbirth (p for trend over quartiles: 0.010). Dietary animal iron intake was not significantly associated with a lower risk of stillbirth. There was no significant association between dietary calcium intake and stillbirth
Darling 2017[112]	Dar es Salaam, Tanzania	2001-2004	HIV-negative pregnant women 12-27 wks gestation	7591	Around 25 (5)	Dietary arginine intake during pregnancy	Fetal loss	Dietary arginine intake was not associated with fetal loss (p = 0.43)
Kyweluk 2018[113]	Cebu, Philippines	Started in 1983	Pregnant women who gave birth during 1983-1984	807	Mean age at menarche (SD): 12.8 (1.2)	Caloric intake and BMI during childhood and adolescence	Age at menarche	Childhood caloric intake and BMI were both significant independent predictors of accelerated menarcheal timing. Early maturers grew faster from birth to 24 mos and had higher caloric intake and BMI in childhood
Jansen 2018[114]	Mexico City, Mexico	1997-2004	Child participants from 2 of 3 sequentially enrolled cohorts of the ELEMENT study. Between 1997 and 2004, mother/child dyads were recruited from prenatal clinics	989	14.5 (2.1)	Dietary patterns during childhood, including vegetables and lean proteins, maize products and sugar-sweetened beverages, processed	Age at menarche	There were no dose-response relations between any of the dietary patterns and menarche

Study	Setting	Time period	Study population	Number of participants in analysis	Age[1]	Exposure	Outcomes of interest	Overall findings
						meats and refined grains, fruit and yogurt, whole grains, and fat		
Arefi 2018[115]	Iran	Not clear	Patients with primary or secondary infertility	189	Range: 21-42; mean (SD): 32.21 (3.64)	Vitamin D status during preconception	AFC	There was a negative association between vitamin D and AFC ($p < 0.001$)
Li 2018[116]	Chongqing, China	2014-2017	Primary school students	1066	Range: 5.81-12.20; mean: 8.59	BMI during childhood	Age at menarche	Higher prepubertal BMI was associated with earlier menarche (HR: 1.205, 95% CI: 1.151-2.261). Girls with overweight (HR: 2.605, 95% CI: 1.716-3.956) and obesity (HR: 2.565, 95% CI: 1.603-4.103) had greater risks of early menarche than those with normal weight
Pan 2018[117]	Zhejiang and Guangxi in China	2010-2017	Patients with PCOS undergoing IVF	1074	Around 28 (3)	BMI during preconception	Biochemical pregnancy rate; clinical pregnancy rate; implantation rate; miscarriage (< 28 wks); live birth rate	There were no significant differences among the patients with PCOS and with normal weight, overweight, and obesity undergoing IVF on the biochemical pregnancy rate, clinical pregnancy rate, miscarriage rate, or live birth rate. The biochemical pregnancy rate, implantation rate, and live birth rate were lower with higher BMI ($p < 0.05$). Patients with underweight achieved higher live birth rate ($p < 0.05$) than patients with PCOS and normal weight
Huang 2018[118]	Zhejiang, China	2010-2014	Women undergoing IVF or ICSI	2277	34.52 (5.46)	BMI during preconception	Live birth rate	There were significant differences in BMI ($p = 0.039$) between the non-live-birth and live-birth groups
Zerfu 2018[119]	Oromia, Ethiopia	Not clear	Pregnant women	374	Not clear	Dietary patterns during preconception	Stillbirth (> 24 wks)	Poor or inconsistent consumption of dairy, dark green leafy vegetables and fruits were associated with a higher risk of stillbirth
Patel 2018[120]	Maharashtra, India	2009-2016	Pregnant women	67574	Not clear but categorized into <20, 20-29, > 29	Anemia and BMI during pregnancy	Stillbirth	Mild anemia (RR: 1.3; 95% CI: 1.1, 1.6) and moderate/severe anemia (RR: 1.4; 95% CI: 1.2, 1.8) at any time during pregnancy was significantly associated with the risk of stillbirth. The risk of stillbirths (RR: 1.5; 95% CI: 1.2, 1.8) was highest when anemia and underweight co-existed
Mocking 2018[121]	Jakarta, Indonesia; Accra, Ghana	2012-2014	Pregnant women	1379 (433 from Indonesia, 946 from Ghana)	Mean (SD): 26.5 (6.06), 29.1 (5.18), and 30.2 (6.06) for the first to third tertiles of BMI in Indonesia; Mean (SD): 26.3 (4.5), 28.0 (5.3), and 29.7 (5.0) for the first to third tertiles of BMI in Ghana	BMI during early pregnancy	Stillbirth (> 24 wks)	Maternal overweight and obesity were associated with higher risks of stillbirth. Anemia was not associated with adverse birth outcomes in the Indonesian (OR 1.29; 95% CI 0.35, 4.76) or the Ghanaian (OR 1.09; 95% CI 0.71, 1.68) cohort

Study	Setting	Time period	Study population	Number of participants in analysis	Age[1]	Exposure	Outcomes of interest	Overall findings
Dhaded 2018[122]	Belagavi, India	2014-2017	Pregnant women	30166	Not clear but split into ≤ 20, 21-25, 26-30, > 30	BMI and Hb during pregnancy	Miscarriage (< 20 wks)	Women with a BMI > 25 were less likely to have a miscarriage at 6 wks to 7 wks 6 days. Those with a Hb < 9 g/dL had a lower risk of miscarriage in all gestational age groups, although the results were only significant in the earliest gestational age group. Those with a Hb level from 9 to 11 g/dL were less likely to have a miscarriage at 6 wks to 7 wks 6 days and at 8 wks to 11 wks 6 days.
Aurino 2018[123]	Andhra Pradesh, India; Telangana, India; Peru; Vietnam	Started 2002-2003	Girls born in 2001-2002	2001	Mean (SD) menarcheal age in mos in 2013: 145.30 (3.82)	Dietary intake, HAZ, BAZ, and mother's nutritional status	Age at menarche	HAZ (HR: 1.66; 95% CI: 1.5, 1.83) and BAZ at 8 yrs (HR: 1.28; 95% CI: 1.18, 1.38) predicted earlier menarche. Changes in HAZ and BMIZ between 1 and 8 yrs were not associated with earlier menarche. By 12 yrs, girls who had menarche were more likely to have consumed animal-sourced protein, fruits, and vegetables the previous day
Miller 2019[124]	Bolivia	2002-2010	Women of reproductive age who had given birth to at least one child	116	Range: 16-45; mean (SD): 28.3 (7.6)	Hb during preconception	Fecundity	The higher the Hb level, the lower the hazard of a woman giving birth within the study observation period (HR: 0.82, *p* = 0.03). However, there was no evidence that low Hb was associated with lower women's fecundity
Zhao 2019[125]	China	2015-2016	Couples who had no prior gravidity	2226048	Range: 20-49	Fasting plasma glucose level during preconception	Time to pregnancy	The cumulative pregnancy rate for 12 cycles of the normal fasting plasma glucose level group was significantly higher than that of the impaired fasting glucose and diabetes groups
Maged 2019[126]	Cairo, Egypt	2012-2017	Women undergoing IVF	185	Around 35 (5)	BMI during preconception	Clinical pregnancy; implantation; chemical pregnancy rate; number of oocytes and embryos	The number of embryos, transferred embryos, and frozen embryos were similar in the three BMI groups. Implantation, chemical pregnancy, and clinical pregnancy rates were significantly higher for women with normal weight than women with overweight and obesity, and for women with overweight than women with obesity
Jahangirifar 2019[127]	Isfahan, Iran	2015-2016	Women with primary infertility undergoing IVF/ICSI	140	Range: 20-45	Dietary patterns during preconception	Number of total oocytes; number of metaphase II oocytes; fertilization rate; biochemical pregnancy; clinical pregnancy	There was a greater average number of metaphase II oocytes (*p* for trend = 0.034) in the third tertile of healthy diet than the first tertile. The western and the unhealthy diets were not associated with the average number of total and metaphase II oocytes. Women in the second tertile of unhealthy diet had a significantly lower chance of getting pregnant than the first tertile. None of

Study	Setting	Time period	Study population	Number of participants in analysis	Age[1]	Exposure	Outcomes of interest	Overall findings
								the dietary patterns were associated with fertilization rate and embryo quality
Zhou 2019a[128]	Hebei, China	2006-2009	Pregnant women in a randomized controlled trial with singleton pregnancies; nulliparous women were enrolled < 20 wks gestation	18481	23.0 (3.0)	BMI during early pregnancy	Miscarriage (< 20 wks, and 20 to < 28 wks), stillbirth (≥ 28 wks), and fetal loss combining the two	Obesity was particularly associated with miscarriage (RR 1.51; 95% CI: 1.13-2.02) but not stillbirth (RR 1.52; 95% CI: 0.65-3.57)
Zhou 2019b[129]	Hebei, China	2006-2009	Pregnant women in a randomized controlled trial with singleton pregnancies; nulliparous women were enrolled < 20 wks gestation	14219	Categorized as <25, 25 to <30, ≥ 30	GWG during pregnancy	Stillbirth (≥ 28 wks)	Quintiles of GWG rate in the second and third trimesters were not significantly associated with stillbirth risk
Zhang 2019[130]	Shanghai, China	2010-2017	Women who had undergone first FET cycles with high-quality embryo transfer after a freeze-all policy	22043	Around 31 (4)	BMI during preconception	Implantation rate; biochemical pregnancy; clinical pregnancy; miscarriage (< 24 wks); live birth rate	Women with underweight had lower rates of implantation (OR: 0.91;95%CI 0.85–0.96), clinical pregnancy (OR0.91; 95% CI 0.83–0.99), and live birth (OR0.91; 95% CI 0.83–0.99) than women with normal weight. Obesity was significantly associated with lower implantation (OR 0.80; 95% CI 0.73–0.87), clinical pregnancy (OR 0.81; 95% CI 0.710.91), and live birth rates (OR 0.70; 95% CI 0.62–0.80). Pregnancy loss rate, both in the first (OR1.46; 95%CI 1.15–1.87) and second trimester (OR 2.76; 95% CI 1.67–4.58), was significantly higher in the obesity group
Parks 2019[131]	Nagpur, India; Belagavi, India; Thatta, Sindh Province, Pakistan	2012-2016	Pregnant women	92247	Not clear but categorized as <20, 20-35, > 35	Hb during pregnancy	Stillbirth	There was a higher rate of stillbirth in women with severe anemia ($p < 0.001$). Anemia was not associated differently with fresh stillbirths versus macerated stillbirths
Svefors 2020[132]	Bangladesh	2016-2017	Children born to women participating in the MINIMat trial	1930	Not clear but categorized as < 20, 20-29, ≥ 30	Stunting during infancy and childhood	Age at menarche	Children with stunting had a later age at menarche than children without stunting
Hur 2020[133]	Gaibandha, Bangladesh; Southern Rangpur, Bangladesh	2001-2007	Adolescent girls married ≤ 4 mos	5516	Range: 12-19	Thinness (defined by MUAC) during preconception	Time to pregnancy	Thinness was associated with a delay in time to pregnancy
Hu 2020[134]	Shanghai, China	2013-2015	Women of reproductive age seeking preconception counseling	820	Range: 24-46; mean (SD): 32.10 (3.25)	BMI during preconception	Fecundability; fecundity; fertility	No difference was found in fecundability and infertility rates in women with underweight or overweight/obesity than in women with normal weight
Alizadeh 2020[135]	Tehran, Iran	2015-2018	Women who underwent assisted reproductive technology	398	31.67 (5.39)	BMI during preconception	Number of total metaphase II oocytes	BMI was significantly and inversely associated with the number of metaphase II oocytes ($p < 0.001$). The expected number of MII was lower by 0.99 for each 1-unit increase in BMI (95% CI: -1.01, -0.98)

Study	Setting	Time period	Study population	Number of participants in analysis	Age[1]	Exposure	Outcomes of interest	Overall findings
Duan 2020[136]	China	1997-2015	Girls aged 6-13 yrs	1118	Mean age at baseline (SD): 8.3 (1.8)	Dietary patterns (modern dietary pattern, animal food pattern, and snack food pattern) during childhood and adolescence	Age at menarche	Girls in the highest quartile of modern dietary pattern had a 33% higher probability of experiencing menarche at an earlier age than those in the lowest quartile (HR: 1.33, 95% CI: 1.002,1.77, *p* for trend = 0.03). No significant association was found for the animal food pattern or snack food pattern
Fang 2020[137]	Chongqing, China	2012-2016	Couples wishing to conceive	50927	Mean (SD): 24.80 (3.18), 25.78 (4.04), and 27.21 (4.97) from women with underweight, normal weight, and overweight/obesity	BMI during preconception	Miscarriage; time to pregnancy	Women with pre-pregnancy overweight/obesity were more likely to experience miscarriage and had a longer time to pregnancy than women with normal weight
Xu 2020[138]	China	2013-2017	Reproductive-aged couples who planned to get pregnant within the next 6 mos	3971428	20-49	Hb during preconception	Miscarriage (< 28 wks)	Severe anemia and high Hb concentration before pregnancy were associated with a higher risk of miscarriage; women with mild anemia prior to pregnancy had a lower risk of miscarriage
Ou 2020[139]	Beijing, China	2013-2017	Women with unexplained recurrent miscarriage	171	Range: 23-39; mean (SD): 32.4 (3.4)	Triple therapy including aspirin, prednisone, and multivitamins during preconception and pregnancy	Rate of successful pregnancy; continuous successful treatment; miscarriage (< 20 wks)	Triple therapy was associated with a higher rate of successful treatment (OR: 6.38; 95% CI: 2.82, 14.42; $p < 0.001$) than FA monotherapy
Mao 2020[140]	Chongqing, China	2010-2016	Pregnant women on FA supplementation	65497	Median (Q1, Q3): 25 (22, 27)	FA supplementation during preconception	Miscarriage (< 28 wks) and stillbirth	Periconceptional FA supplementation was associated with a lower risk of miscarriage (RR: 0.52; 95% CI: 0.48, 0.56). Pregnant women with FA supplementation initiated at least 3 mos before conception had a 10% lower risk of miscarriage (RR: 0.46; 95% CI: 0.42, 0.50) than those with FA supplementation initiated 1-2 mos before conception (RR: 0.56; 95% CI: 0.50, 0.62) or after conception (RR: 0.56; 95% CI: 0.51, 0.61). Pregnant women with FA supplementation had a 70% lower risk of stillbirth
Madlala 2020[141]	Cape Town, South Africa	2015-2016	HIV-positive pregnant women	471	Median (Q1, Q3): 28 (23, 32)	BMI during pregnancy	Miscarriage (< 28 wks) and stillbirth (≥ 28 wks) combined	No significant associations were observed between obesity and stillbirth/miscarriage
Madzorera 2020[142]	Dar es Salaam, Tanzania	2001-2004	HIV-negative pregnant women of 12-27 wks gestation	7553	Range: 18-45; mean (SD): around 25 (5)	Dietary diversity and dietary quality during pregnancy	Fetal loss, including miscarriage (< 28 wks) and	Dietary diversity was not significantly associated with fetal loss. An inverse association was found between dietary quality and fetal loss

Study	Setting	Time period	Study population	Number of participants in analysis	Age[1]	Exposure	Outcomes of interest	Overall findings
							stillbirth (≥ 28 wks)	
Kutchi 2020[143]	Salem, Tamilnadu, India	2016-2017	Pregnant women up to 16 wks gestation	200	Around 26 (4)	BMI during early pregnancy	Miscarriage	Mothers with obesity had a significantly greater risk of miscarriage than mothers without obesity (OR: 4.85; 95% CI: 1.02, 23.03)
Ali 2020[144]	Sindh, Pakistan; Belagavi, India; Nagpur, India	2012-2018	Pregnant women	130888	Not clear but categorized into < 20, 20-35, and > 35	Hb during pregnancy	Stillbirth	Risk of stillbirth was higher in women with a Hb < 10 g/dL than the group with a Hb concentration of 11-12.9 g/dL
Mali 2021[145]	Belagavi, South India	2017	Women admitted to the labor ward and had stillbirth	5755	Not clear	Anemia during pregnancy	Stillbirth (> 20 wks)	Anemia was positively associated with stillbirths; the odds of stillbirth were 21.87 (95% CI: 15.69, 30.48) times higher for the subjects with anemia than the subjects without anemia
Cai 2021[146]	Xi'an, China	2014-2019	Women who underwent IVF/ICSI	21820	Around 30 (4)	BMI during pregnancy	EPL (≤ 13 wks); LPL (> 13 wks)	BMI was associated with a greater risk of LPL (OR 1.08, 95% CI 1.04–1.1)
Wu 2021[147]	Shanghai, China	2015	Girls from primary and middle schools	986	Not clear	Meat intake during childhood and adolescence	Age at menarche	A lower consumption of poultry was associated with a lower risk of earlier age of menarche (*p* for trend < 0.05). Girls who never consumed poultry had a lower risk of earlier menarche than those who consumed poultry at least once a wk (OR: 0.61; 95% CI: 0.39, 0.96)
Gao 2021[148]	China	2012-2015	Women with PCOS	998	Around 28 (3)	BMI during preconception	Ovulations; conception; live births; clinical pregnancy; pregnancy loss	Rates of ovulation ($p < 0.001$), conception ($p = 0.028$), pregnancy ($p = 0.023$), and live birth ($p = 0.030$) became lower with increasing BMI category. Pregnancy loss rates were comparable in all three BMI groups
Wang 2021[149]	Beijing and Shandong, China	2015-2017	Women with infertility undergoing their first IVF-ET treatment cycles	305	31.4 (3.4)	Zinc concentrations during preconception	Clinical pregnancy	Women who failed to achieve clinical pregnancy had significantly lower zinc concentrations in Shandong, but not in Beijing or the total participants in both centers. In Shandong, women who achieved clinical pregnancy were found to have significantly higher serum zinc concentrations than those who failed ($p = 0.025$)
Chaurasia 2021[150]	Gaya, Bihar, India	2019-2020	Pregnant women with viable singleton pregnancy	100	23.98 (3.76)	BMI during early pregnancy	Stillbirth	There were three stillbirths among women with normal BMI, zero stillbirths among women with low BMI, one among women with overweight, and one among women with obesity
Ambedkar 2021[151]	Uttar Pradesh, India	2019-2021	Pregnant women with varying severity of anemia and pregnant women without anemia	476	Not clear	Anemia during pregnancy	Miscarriage, stillbirth, IUD	Miscarriage, stillbirth, and IUD were associated with the severity of anemia
Yang 2021a[152]	Dazu and Chongqing, China	2013-2018	Women of reproductive age receiving reproductive consultation	2677	Not clear but categorized as <20, 20-30, 30-40, 40-45	FA supplementation during preconception and pregnancy (including individualized FA dose based on maternal genotype, and	Miscarriage	The individualized FA supplement had a lower incidence of miscarriage ($p < 0.01$) than no FA supplementation

Study	Setting	Time period	Study population	Number of participants in analysis	Age[1]	Exposure	Outcomes of interest	Overall findings
						conventional unified FA dose)		
Yang 2021b[153]	China	2014-2019	Patients who underwent assisted reproductive technology	15210	Range: 20-45; mean (SD): 29.94 (4.17)	BMI during preconception	Pregnancy loss, including early miscarriage (< 12 wks), late miscarriage (12-24 wks), and stillbirth (≥ 28 wks)	Patients with overweight had a higher risk of late miscarriage (OR 1.38, 95% CI 1.16-1.65), while patients with obesity had a higher risk of both early miscarriage (OR 1.47, 95% CI 1.14-1.91) and late miscarriage (OR 1.80, 95% CI 1.33-2.44)
Yang 2021c[154]	China	2013-2014	Couples who had undergone IVF/ICSI	17978	31.96 (4.76)	BMI during preconception	Live birth rate	Greater preconceptional BMI was significantly associated with a lower live birth rate
Tang 2021[155]	Guangdong Province, China	2013-2017	Pregnant women	668956	Median (Q1, Q3): 26 (24, 29)	BMI during preconception	Stillbirth	Compared with healthy weight, underweight was inversely associated with stillbirth (IRR: 0.73; 95% CI: 0.53, 0.99), and overweight was associated with higher risk of stillbirth (IRR:1.44; 95% CI: 1.03, 2.06)
Qu 2021[156]	Xi'an, China	2006-2015	Pregnant women who underwent assisted reproductive technology	14994	29.79 (4.08)	BMI during preconception	Miscarriage (< 20 wks), including early miscarriage (< 12 wks)	The risks of early miscarriage and miscarriage were higher in the obesity group than in the group with normal weight (early miscarriage: RR: 1.36; 95% CI: 1.12, 1.65; miscarriage: RR: 1.40, 95% CI: 1.17, 1.68). Pre-pregnancy underweight was not associated with a greater risk of early miscarriage or miscarriage
Hu 2021[157]	Fuyang, Anhui, China	2010-2013	Women of reproductive age	17248	Range: 18-45; mean (SD): 24.27 (3.38)	Alcohol consumption, FA use, BMI, serum creatinine, and anemia during preconception	Miscarriage (< 20 wks)	Serum creatinine was associated with a greater risk of miscarriage (> 80 mmol/L; OR: 1.35; 95% CI: 1.16, 1.57). FA use 3 or more mos before conception (OR: 0.69; 95% CI: 0.57, 0.83), 1 to 2 mos before conception (OR: 0.70; 95% CI: 0.54, 0.91), and after conception (OR: 0.81; 95% CI: 0.67, 0.98) was associated with the lower risk of miscarriage. Alcohol consumption, BMI, and preconceptional anemia were not associated with miscarriage
Chen 2021[158]	Sichuan, Yunnan, and Guizhou, China	2013-2018	A representative sample of pregnant women and their children	51125	30.7 (4.2)	BMI during preconception	Stillbirth (> 24 wks)	Women with pre-pregnancy overweight or obesity (but not underweight) are at a higher risk of stillbirth (OR: 1.69; 95% CI: 1.41, 2.02)
Bakleicheva 2021[159]	St. Petersburg, Russia	2018-2020	Pregnant women in the first trimester of pregnancy (up to 13 wks)	88	20-44	Vitamin D status during the first trimester of pregnancy	Threat of miscarriage; missed abortion; diagnosis of infertility	Threat of miscarriage, missed abortion, and infertility were positively associated with vitamin D deficiency
Jessani 2021[160]	Pakistan; India; Zambia; Kenya; Democratic	2016-2019	Nulliparous between 6-<14 wks gestation	11558	21.0 (3.0)	Hb during pregnancy	Stillbirth (≥ 20 wks)	There was a U-shaped relationship showing a higher proportion of fetal deaths at both lower (70-89 g/L) and

Study	Setting	Time period	Study population	Number of participants in analysis	Age[1]	Exposure	Outcomes of interest	Overall findings
	Republic of Congo; Guatemala							higher (110-129 g/L) concentrations of Hb
Unisa 2022[161]	India	2015-2016	Married women	402807	20-49	Dietary patterns, BMI, and anemia during preconception	Primary infertility	Primary infertility was higher among those with lower intake of milk or curd, dark green leafy vegetables, and fruit. Primary infertility was higher among those with a high intake of fish, eggs, chicken, meat, and aerated drinks. Primary infertility was higher among women with overweight/obesity than those with lower BMI. Women without anemia had a lower prevalence of primary infertility than women with severe anemia
Li 2022[162]	Guangzhou, China	2017	Patients with PCOS	160	Around 28 (4)	Body weight reduction during preconception	Spontaneous pregnancy	Body weight reduction was positively associated with spontaneous pregnancy
Wu 2022[163]	Shenyang, China	2017-2020	Couples recruited in a prospective cohort study	1500	32.09 (4.33)	Dietary patterns during preconception	Biochemical pregnancy; clinical pregnancy; live birth; early miscarriage (< 12 wks); late miscarriage (12- < 18 wks)	Women who are more inclined to the Fruits-Vegetables-Dairy-Eggs pattern and less adherent to the Tubers-Beans-Cereals were more likely to achieve normally fertilized eggs and transferable embryos. A lower Puffed food-Candy-Bakery score and a higher Dried fruits-Organs-Rice score were related to a higher likelihood of achieving biochemical pregnancy
Zhang 2022[164]	Shanghai, China	2016-2018	Preconception couples who attempted to conceive	198	Range: 21-38; mean: 28	Vitamin D concentration during preconception	Time to pregnancy; clinical pregnancy	Preconceptional vitamin D insufficiency (< 30 ng/mL) was not significantly associated with time to pregnancy
Zhu 2022[165]	Shanghai, China	2013-2017	Couples of reproductive ages	1839	Range: 20-49; mean (SD): 29.76 (3.71)	BMI during preconception	Infertility	High BMI (especially BMI ≥ 28) was associated with a greater risk of infertility (OR: 1.58; 95% CI 1.31, 6.26)
Xiong 2022[166]	China	2013-2018	Children during middle childhood	4781	Range: 6-8; mean (SD): 7.2 (0.7)	Dietary soy and fiber intake	Age at menarche	Total fiber intake was not significantly associated with puberty timing after adjusting for soy intake
Xu 2022[167]	China	1997-2015	Girls during middle childhood	5920	7.0 (0.8)	Dietary fat intake	Age at menarche	Girls with a higher intake of dietary fat were more likely to experience menarche (HR: 1.17; 95% CI, 1.11, 1.23; *p* for trend = 0.01) earlier than those with a lower intake of dietary fat. Girls with a higher PUFA intake had approximately higher HR to experience menarche (HR: 1.13; 95% CI, 1.08, 1.20; *p* for trend = 0.03) than girls with a lower PUFA intake. Higher MUFA intake in girls was associated with earlier menarche (HR: 1.11; 95% CI, 1.06, 1.17; *p* for trend = 0.05)
Chai 2022[168]	China	2013-2016	Couples who were planning a pregnancy within the next 6 mos	4531680	20 to 49	Alcohol intake during preconception	Miscarriage (< 28 wks)	Compared with non-drinkers, the OR of miscarriage was 1.06 (95% CI 1.02,

Study	Setting	Time period	Study population	Number of participants in analysis	Age[1]	Exposure	Outcomes of interest	Overall findings
								1.10) and 1.59 (95% CI 1.15, 2.20) in maternal occasional drinkers and regular drinkers, respectively
Zheng 2022[169]	China	2016-2019	Couples in a reproductive medicine center	10252	Around 32 (4)	BMI during preconception	Live birth rate; clinical pregnancy rate; miscarriage	Miscarriage rate was higher in women with obesity than in women with normal weight (OR: 1.453; 95% CI: 1.066, 1.982). BMI had no significant effect on the chance of pregnancy or live birth
Shi 2022[170]	China	2016-2019	Pregnant women	18948443	29.42 (4.87)	Anemia during pregnancy	Stillbirth (> 20 wks)	Compared with no anemia, mild anemia (OR: 0.59, 95% CI: 0.58, 0.61) and moderate anemia (OR: 0.79; 95% CI: 0.76, 0.81) were associated with a lower risk of stillbirth, and severe anemia was associated with a higher risk of stillbirth (OR: 1.86; 95% CI: 1.75, 1.98)
Chen 2022[171]	China	2017-2018	Patients with infertility undergoing IVF/ICSI	2569	Range: 18-39; median (Q1, Q3): 29 (27,32)	BMI during preconception	Clinical pregnancy rate; implantation rate; ongoing pregnancy rate; early miscarriage rate (< 12 wks); live birth rate	BMI was not associated with IVF/ICSI pregnancy outcomes
Wondemagegn 2022[172]	East Gojjam, Amhara, Ethiopia	2019-2020	Pregnant women	390	27 (4.53)	Dietary diversity during pregnancy	Stillbirth	There was no significant association between dietary diversity during pregnancy and the risk of stillbirth
Perumal 2022[173]	Dar es Salaam, Tanzania	2001-2005	HIV-negative pregnant women of 12-27 wks gestation	7561	Not clear but categorized into < 20, 20-24, 25-29, and ≥ 30	GWG during pregnancy	Stillbirth (≥ 28 wks)	Total excessive GWG was positively associated with the risk of stillbirth (RR 1.60, 95% CI: 1.03, 2.42), especially among women with underweight (RR 3.13, 95% CI: 0.96, 10.2) than women with normal weight during early pregnancy (RR: 1.73; 95% CI: 0.95, 3.15; *p* for interaction = 0.04)
Caniglia 2022[174]	Botswana	2014-2020	Pregnant women with singleton pregnancies < 24 wks gestation	96341	Not clear	Use of iron, FA, or MMS during pregnancy	Stillbirth (≥ 24 wks)	Women who initiated iron-only and FA-only supplementation had higher risks of stillbirth than women who received iron and FA (risk difference: 0.56%, 95% CI 0.31 to 0.81)
Kamenju 2022[175]	Dar es Salaam, Tanzania	2001-2004	HIV-negative pregnant women 12-27 wks gestation	7564	Median (IQR): 25.2 (5.1)	Dietary consumption of animal source foods during pregnancy	Stillbirth (≥ 28 wks)	Dietary animal protein intake was not significantly associated with stillbirth (*p* = 0.31)
Kalla 2022[176]	Eastern Algeria	2011-2015	Pregnant women	786	17-53	BMI, anemia, and dietary intake during pregnancy	Miscarriage (< 24 wks)	Miscarriage was associated with high BMI (> 25) (OR: 1.88; 95% CI:1.28, 2.78; *p* = 0.001) and diets rich in meat (OR: 0.60; 95% CI: 0.33, 1.04; *p* = 0.075) and moderate in fish (OR: 2.32; 95% CI: 1.18, 4.58; *p* = 0.015)
Abioye 2023[177]	Dar es Salaam, Tanzania	2010-2013	HIV-negative pregnant women of 12-27 wks gestation	1450	Range: 18-45; median (Q1, Q3): 23 (21, 26)	Iron-deficiency anemia during pregnancy	Stillbirth (≥ 28 wks)	Iron-deficiency anemia was associated with a greater risk of stillbirths (RR: 2.10; 95% CI 1.05, 4.24) than the non-anemia group

Study	Setting	Time period	Study population	Number of participants in analysis	Age[1]	Exposure	Outcomes of interest	Overall findings
Yu 2023[178]	China	2018-2021	Women who had received IVF/ICSI-ET	612	Not clear but categorized as 20-30, 30-35, > 35	Vitamin D status during preconception	Clinical pregnancy rate and live birth rate	Women with a sufficient vitamin D concentration had a higher chance of successful birth outcomes than women with vitamin D insufficiency
Eldin 2023[179]	Cairo, Egypt	2016-2020	Pregnant women treated by assisted reproductive technology	195	Around 30 (5)	BMI during early pregnancy	EPL (6-12 wks)	There was no significant association between BMI category and EPL
Gudipally 2023[180]	Telangana, India	2009-2011	Married women were recruited before conception or within the first trimester of pregnancy	675	Range: 15-35	BMI during preconception	Miscarriage (< 20 wks) and fetal death (including stillbirth ≥ 20 wks and miscarriage)	BMI during preconception was not significantly associated with miscarriage or fetal death

AFC, antral follicle count; BAZ, BMI-for-age z-score; BMI, body mass index; CLHNS, Cebu Longitudinal Health and Nutrition Survey; CI, confidence interval; EPL, early pregnancy loss; ET, embryo transfer; FA, folic acid; FET, frozen embryo transfer; FSH, follicle-stimulating hormone; GWG, gestational weight gain; HAZ, height-for-age z-score; Hb, hemoglobin; HIV, human immunodeficiency virus; HR, hazard ratio; ICSI, intracytoplasmic sperm injection; INCAP, Institute of Nutrition of Central America and Panama; IQR, interquartile range; IRR, incidence risk ratio; IUD, intrauterine death; IUFD, Intrauterine fetal death; IVF, in vitro fertilization; LH, luteinizing hormone; LPL, late pregnancy loss; MMA, methylmalonic acid; MMS, multiple micronutrient supplementation; MUAC, mid-upper arm circumference; MUFA, monounsaturated fatty acid; OR, odds ratio; PCOS, polycystic ovary syndrome; Q1, 25th percentile; Q3, 75th percentile; PUFA, polyunsaturated fatty acid; RR, risk ratio; SD, standard deviation; WAZ, weight-for-age z-score; WHZ, weight-for-height z-score.

[1] The values are total range or means (standard deviations) in years unless stated otherwise.

Supplemental Table 8. Exposure-outcome combinations examined in the observational cohort studies

Outcome	Exposure	Number of Studies (number of studies with quantitative estimates for meta-analysis)
Miscarriage	BMI during preconception	19 (17)
	Hb concentrations and/or anemia during pregnancy	6 (4)
	BMI during early pregnancy	5 (4)
	Use of folic acid during preconception and/or pregnancy	4
	Alcohol intake during preconception and/or pregnancy	3
	Hb concentrations and/or anemia during preconception	2
	Dietary patterns during pregnancy	2
	Vitamin B6 status during preconception	2
	Vitamin D status during pregnancy	2
	MUAC during pregnancy	1
	Dietary patterns during preconception	1
	Vitamin B12 status during preconception	1
	Folate status during preconception	1
	Homocysteine status during preconception	1
	MMA status during early pregnancy	1
	Use of multivitamins during early pregnancy	1
	Serum copper status during preconception	1
	Serum copper status during pregnancy	1
	Serum zinc status during preconception	1
	Serum zinc status during pregnancy	1
	Serum calcium status during preconception	1
	Serum calcium status during pregnancy	1
	Serum iron status during preconception	1
	Serum iron status during pregnancy	1
	Triple therapy for infertility that included multivitamins	1
	Serum creatinine	1
Stillbirth	Hb concentrations and/or anemia during pregnancy	18 (17)
	BMI during early pregnancy	5
	BMI during preconception	7
	GWG	4
	MUAC during pregnancy	2
	Height during pregnancy	3
	Use of iron supplementation during pregnancy	2
	Dietary diversity during pregnancy	2
	Use of folic acid during preconception and/or pregnancy	2
	Dietary consumption of animal source foods during pregnancy	1
	Iodine deficiency during pregnancy	1
	Dietary patterns during pregnancy	1
	Dietary patterns during preconception	1
	Weight during pregnancy	1
	Dietary iron intake during pregnancy	1
	Dietary calcium intake during pregnancy	1
	Vitamin B12 status during pregnancy	1
	Vitamin D status during pregnancy	1
	Use of multivitamins during pregnancy	1
Composite outcome of fetal death (including stillbirth and miscarriage)	Hb concentrations and/or anemia during pregnancy	6
	BMI during preconception	2
	BMI during early pregnancy	4
	GWG	2
	Dietary arginine intake during pregnancy	1
	Plasma selenium status during pregnancy	1
	Dietary diversity during pregnancy	1
	Dietary quality during pregnancy	1
Ovulation rate	Weight loss after bariatric surgery	1
	BMI during preconception	1
Conception	Vitamin B6 status during preconception	1
	Homocysteine status during preconception	1
Biochemical pregnancy	BMI during preconception	5
	Dietary patterns during preconception	2
Clinical pregnancy	BMI during preconception	16 (15)
	Vitamin B6 status during preconception	2
	Vitamin D status during preconception	2
	Dietary patterns during preconception	2
	Vitamin B12 status during preconception	1
	Folate status during preconception	1
	Homocysteine status during preconception	1

	Weight loss after bariatric surgery	1
	Zinc status during preconception	1
Number of oocytes	BMI during preconception	5
	Dietary patterns during preconception	1
Live birth rate	BMI during preconception	14
	Dietary patterns during preconception	1
	Vitamin D status during preconception	1
Infertility rate	BMI during preconception	4
	Hb concentrations and/or anemia during preconception	2
	Vitamin D status during pregnancy	1
	Dietary patterns during preconception	1
Fertilization rate	BMI during preconception	2
	Dietary patterns during preconception	1
Implantation rate	BMI during preconception	8 (6)
Number of metaphase II/mature oocytes	BMI during preconception	2
	Dietary patterns during preconception	1
Number of total embryos	BMI during preconception	2
Antral follicle count	BMI during preconception	1
	Vitamin D status during preconception	1
LH	BMI during preconception	2
	Weight gain during adolescence	1
	Height gain during adolescence	1
FSH	BMI during preconception	2
	Weight gain during adolescence	1
	Height gain during adolescence	1
Ovarian volume	BMI during preconception	2
	Weight gain during adolescence	1
	Height gain during adolescence	1
Length of postpartum amenorrhea	Nutritional status during pregnancy	1
Duration of postpartum amenorrhea	BMI during postpartum period	1
	Fat intake during postpartum period	1
Duration from return to menses to conception	BMI during postpartum period	1
	Fat intake during postpartum period	1
Age at menarche	Length/height/HAZ/stunting during childhood and/or adolescence	9
	Weight/WAZ during childhood and/or adolescence	3
	BMI/BAZ during childhood and/or adolescence	3
	Body composition during childhood and/or adolescence	2
	Meat intake during childhood and/or adolescence	2
	Dietary patterns during childhood and/or adolescence	2
	Birth weight	1
	Birth length	1
	WLZ/WHZ during childhood and/or adolescence	1
	Vitamin D status during childhood and/or adolescence	1
	Food insecurity during childhood/adolescence	1
	Fish intake during childhood and/or adolescence	1
	Animal-source protein intake during childhood and/or adolescence	1
	Fruit intake during childhood and/or adolescence	1
	Vegetable intake during childhood and/or adolescence	1
	Hb concentrations and/or anemia	1
	Plasma zinc status	1
	Plasma vitamin B12 status	1
	Plasma folate status	1
	Plasma retinol status	1
	Caloric intake during childhood and/or adolescence	1
	Dietary soy intake	1
	Dietary fiber intake	1
	Dietary fat intake	1
Time to pregnancy	Fasting plasma glucose level during preconception	1
	MUAC during preconception	1
	BMI during preconception	1
	Vitamin D status during preconception	1
Amenorrhea	BMI during preconception	1
Oligomenorrhea	BMI during preconception	1
Irregular menstrual cycles	BMI during preconception	1
Early pregnancy loss/early miscarriage	BMI during preconception	5
	BMI during early pregnancy	2
	Dietary patterns during preconception	1
Late pregnancy loss	BMI during early pregnancy	1
	BMI during preconception	1
	Dietary patterns during preconception	1

BAZ, BMI-for-age z-score; FSH, follicle-stimulating hormone; GWG, gestational weight gain; HAZ, height-for-age z-score; Hb, hemoglobin; LH, luteinizing hormone; MMA, methylmalonic acid; MUAC, mid-upper arm circumference; WAZ, weight-for-age z-score; WHZ, weight-for-height z-score; WLZ, weight-for-length z-score.

Supplemental Table 9. The definitions used to define different outcomes in the identified studies[1]

Outcome	Definition
Miscarriage[1]	The loss of a pregnancy before viability, most frequently defined as the loss of pregnancy before 28 weeks of gestation
Stillbirth[1]	The loss of a pregnancy after a given threshold, most frequently defined as a baby born with no signs of life at or after 28 weeks
Early miscarriage	The loss of pregnancy before reaching 12 weeks of gestation
Early pregnancy loss	A biochemical pregnancy without subsequent ultrasound signs of viable pregnancy
Implantation rate	The ratio of the number of gestational sacs over the number of transferred embryos
Biochemical pregnancy	A pregnancy that is confirmed by the presence of serological human chorionic gonadotropin
Clinical pregnancy	A pregnancy confirmed by ultrasound by visualization of a gestational sac or fetal cardiac activity after 4-7 weeks of embryo transfer or gestation
Live birth rate	The proportion of all in vitro fertilization cycles or embryo transfers that result in the birth of a living child

[1] The definitions most commonly used in the included studies are shown in the table, with some variations across studies. For example, variations to the definitions of miscarriage and stillbirth have been observed, with some studies using 20, 22, or 24 weeks to differentiate between the two adverse pregnancy outcomes.

Supplemental Table 10. Risk of bias assessment for the observational cohort studies.[1]

Study	RoB necessary[2]	D1	D2	D3	D4	D5	D6	D7	Overall
Pharoah 1976	No	NA	NA	NA	NA	NA	NA	NA	Critical
Kurz 1993	Yes	Low	Very high	High	Low	High	Low	Low	Serious
Popkin 1993	Yes	Low	Very high	High	Low	High	Low	Low	Critical
Khan 1996	Yes	High	Low	Low	Low	Low	Low	Low	Serious
Onadeko 1996	No	NA	NA	NA	NA	NA	NA	NA	Critical
Agarwal 1998	No	NA	NA	NA	NA	NA	NA	NA	Critical
Simondon 1998	No	NA	NA	NA	NA	NA	NA	NA	Critical
Ramakrishnan 1999	Yes	Low	Low	Some concerns	Low	Low	Low	Low	Moderate
Adair 2001	Yes	Low	Some concerns	Low	Low	Low	Low	Low	Low
Gindler 2001	Yes	Low	Low	High	Low	High	High	Low	Serious
Khan 2001	Yes	Low	Low	Some concerns	Low	Low	Low	Low	Moderate
Osman 2001	Yes	Low	Low	Some concerns	Low	Low	Low	Low	Moderate
Chalumeau 2002	No	NA	NA	NA	NA	NA	NA	NA	Critical
Lone 2004	Yes	Low	Low	Some concerns	Low	Low	Low	Low	Moderate
Villamor 2004	Yes	Low	Low	Low	Low	Low	Low	Low	Low
Kupka 2005	Yes	High	Low	Some concerns	Low	High	Low	Low	Critical
Prabhakar 2007	No	NA	NA	NA	NA	NA	NA	NA	Critical
Ronnenberg 2007	Yes	Low	Low	Some concerns	Low	Some concerns	Low	Low	Serious
Watson-Jones 2007	Yes	Low	Low	Low	Low	Some concerns	Low	Low	Serious
Bosch 2008	Yes	Low	Low	High	Low	High	High	Low	Serious
Hauger 2008	Yes	High	Low	Some concerns	Low	High	Low	Low	Critical
Mehta 2008	Yes	Low	Low	Low	Low	Some concerns	High	Low	Serious
Zhang 2009	Yes	Low	Low	Low	Low	Low	Low	Low	Low
Abeysena 2010	Yes	Low	High	Low	Low	High	Low	Low	Serious
Chumak 2010	Yes	Low	Low	Some concerns	Low	Low	Low	Low	Moderate
Guerra-Shinohara 2010	Yes	Low	Low	Some concerns	Low	Some concerns	High	Low	Moderate
Li 2010	Yes	Low	Low	Some concerns	Low	Low	Low	Low	Moderate
Mesa 2010	Yes	Low	Low	Some concerns	Low	Low	Low	Low	Moderate
Zhang 2010	Yes	Low	Low	Some concerns	Low	Low	Low	Low	Moderate
Belachew 2011	Yes	Low	Low	Some concerns	Low	Low	Low	Low	Moderate
Chumak 2011	Yes	Low	Low	Low	Low	Low	Low	Low	Low
Stringer 2011	Yes	High	Low	Some concerns	Low	High	Low	Low	Critical
Villamor 2011	Yes	Low	Low	Low	Low	Some concerns	Low	Low	Moderate
Gonzales 2012	Yes	Low	Low	Some concerns	Low	Low	Low	Low	Moderate
Reyes 2012	Yes	Low	Low	Some concerns	Low	Low	Low	Low	Moderate
Young 2012	Yes	Low	Low	Some concerns	Low	Low	Low	Low	Moderate
Kumari 2013	Yes	Low	Low	Some concerns	Low	Low	Low	Low	Moderate
Malhotra 2013	Yes	Low	Low	Some concerns	Low	Low	Low	Low	Moderate
Neumann 2013	Yes	High	Low	Some concerns	Low	High	Low	Low	Critical
Cung 2014	Yes	Low	Low	Some concerns	Low	Low	Low	Low	Moderate
Huang 2014	Yes	Low	Low	Some concerns	Low	Low	Low	Low	Moderate
Ouyang 2014	Yes	Low	Low	Low	Low	Low	High	Low	Serious
Sharma 2014	Yes	Low	Low	Low	Low	Low	Low	Low	Low
Zhou 2014	Yes	Low	Low	Low	Low	Low	Low	Low	Low
Awan 2015	No	NA	NA	NA	NA	NA	NA	NA	Critical
Meng 2015	Yes	Low	Low	Some concerns	Low	Low	Low	Low	Moderate
Salgin 2015	Yes	Low	Low	Some concerns	Low	Low	Low	Low	Moderate
Shen 2015	No	NA	NA	NA	NA	NA	NA	NA	Critical
Zhai 2015	Yes	Low	Low	Low	Low	Some concerns	Low	Low	Serious

Jansen 2016	Yes	Low	Low	Some concerns	Low	Low	Low	Low	Moderate
Pan 2016	Yes	Low	Low	Low	Low	Low	Low	Low	Low
Shen 2016	No	NA	NA	NA	NA	NA	NA	NA	Critical
Zerfu 2016	Yes	Low	Some concerns	Some concerns	Low	Some concerns	Low	Low	Moderate
Zhou 2016	Yes	Low	Low	Low	Low	Some concerns	Low	Low	Moderate
Ahmed 2017	No	NA	NA	NA	NA	NA	NA	NA	Critical
Aydin 2017	Yes	High	Low	Low	Low	Some concerns	Low	Low	Serious
Cai 2017	Yes	Low	Low	Some concerns	Low	Low	Low	Low	Serious
Darling 2017	Yes	Low	Low	Some concerns	Low	Low	Low	Low	Moderate
MacKenna 2017	Yes	Low	Low	Some concerns	Low	Low	Low	Low	Moderate
Mosha 2017	Yes	Low	Low	Low	Low	Low	Low	Low	Low
Narasati 2017	No	NA	NA	NA	NA	NA	NA	NA	Critical
Sheng 2017	No	NA	NA	NA	NA	NA	NA	NA	Critical
Kumar 2017	No	NA	NA	NA	NA	NA	NA	NA	Critical
Suryanarayana 2017	Yes	Low	Low	Some concerns	Low	Low	Low	Low	Moderate
Villamor 2017	Yes	Low	Low	Some concerns	Low	Low	Low	Low	Moderate
Arefi 2018	Yes	Low	Low	Some concerns	Low	Low	Low	Low	Moderate
Aurino 2018	Yes	Low	Low	Some concerns	Low	Low	Low	Low	Moderate
Dhaded 2018	Yes	Low	Low	Low	Low	Low	High	Low	Serious
Huang 2018	Yes	Low	Low	Some concerns	Low	Low	Low	Low	Moderate
Jansen 2018	Yes	Low	Low	Low	Low	Low	Low	Low	Low
Kyweluk 2018	Yes	Low	Low	Some concerns	Low	Low	Low	Low	Moderate
Li 2018	Yes	Low	Low	Some concerns	Low	Low	Low	Low	Moderate
Mocking 2018	Yes	Low	Low	Some concerns	Low	Low	Low	Low	Moderate
Pan 2018	Yes	Low	Low	Some concerns	Low	Low	Low	Low	Moderate
Patel 2018	Yes	Low	Low	Low	Low	Some concerns	High	Low	Serious
Zerfu 2018	No	NA	NA	NA	NA	NA	NA	NA	Critical
Jahangirifar 2019	Yes	Low	Low	Some concerns	Low	Low	Low	Low	Moderate
Madlala 2019	Yes	Low	Low	Some concerns	Low	Low	Low	Low	Moderate
Maged 2019	Yes	Low	Low	Low	Low	Some concerns	High	Low	Serious
Miller 2019	Yes	Low	Low	Some concerns	Low	Low	Low	Low	Moderate
Parks 2019	Yes	Low	Low	Some concerns	Low	Low	Low	Low	Moderate
Zhang 2019	Yes	Low	Low	Some concerns	Low	Low	Low	Low	Moderate
Zhao 2019	Yes	Low	Low	Low	Low	Some concerns	Low	Low	Serious
Zhou 2019a	Yes	Low	Low	Low	Low	Some concerns	High	Low	Serious
Zhou 2019b	Yes	Low	Low	Low	Low	Some concerns	Low	Low	Serious
Ali 2020	Yes	Low	Low	Some concerns	Low	Low	Low	Low	Moderate
Alizadeh 2020	Yes	Low	Low	Some concerns	Low	Low	Low	Low	Moderate
Duan 2020	Yes	Low	Low	Low	Low	Low	Low	Low	Low
Fang 2020	Yes	Low	Low	Low	Low	Low	Low	Low	Low
Hu 2020	Yes	Low	Low	Low	Low	Low	High	Low	Serious
Hur 2020	Yes	Low	Low	Low	Low	Low	High	Low	Serious
Kutchi 2020	Yes	Low	Low	Some concerns	Low	Some concerns	Low	Low	Serious
Madzorera 2020	Yes	Low	Low	Low	Low	Low	Low	Low	Serious
Mao 2020	Yes	Low	Low	Low	Low	Low	Low	Low	Low
Ou 2020	Yes	High	Low	Some concerns	Low	High	Low	Low	Critical
Svefors 2020	Yes	Low	Low	Low	Low	Low	Low	Low	Low
Xu 2020	Yes	Low	Low	Low	Low	Low	Low	Low	Low
Ambedkar 2021	No	NA	NA	NA	NA	NA	NA	NA	Critical
Bakleicheva 2021	No	NA	NA	NA	NA	NA	NA	NA	Critical
Cai 2021	Yes	Low	Low	Low	Low	Low	Low	Low	Low
Chaurasia 2021	No	NA	NA	NA	NA	NA	NA	NA	Critical

Chen 2021	Yes	Low	Low	Some concerns	Low	Low	High	Low	Serious
Gao 2021	No	NA	NA	NA	NA	NA	NA	NA	Critical
Hu 2021	Yes	Low	Low	Low	Low	Low	Low	Low	Low
Jessani 2021	Yes	Low	Some concerns	Some concerns	Low	Some concerns	Low	Low	Moderate
Mali 2021	Yes	Low	Low	Low	Low	Low	High	Low	Serious
Qu 2021	Yes	Low	Low	Some concerns	Low	Some concerns	Low	Low	Serious
Tang 2021	Yes	Low	Low	Low	Low	Some concerns	Low	Low	Serious
Wang 2021	Yes	Low	Low	Some concerns	Low	Low	Low	Low	Moderate
Wu 2021	Yes	Low	Low	Low	Low	Some concerns	High	Low	Serious
Yang 2021a	Yes	Low	Low	Low	Low	Some concerns	Low	Low	Moderate
Yang 2021b	Yes	Low	Low	Some concerns	Low	Some concerns	High	Low	Moderate
Yang 2021c	Yes	Low	Low	Low	Low	Low	Low	Low	Serious
Caniglia 2022	Yes	Low	Some concerns	Some concerns	Low	Some concerns	Low	Low	Moderate
Chai 2022	Yes	Low	Low	Low	Low	Some concerns	High	Low	Serious
Chen 2022	Yes	Low	Low	Some concerns	Low	Low	Low	Low	Moderate
Kalla 2022	Yes	Low	Low	Low	Low	Some concerns	High	Low	Serious
Kamenju 2022	Yes	Low	Low	Low	Low	Some concerns	High	Low	Serious
Li 2022	Yes	Low	Low	Low	Low	Some concerns	Low	Low	Serious
Perumal 2022	Yes	Low	Low	Low	Low	Some concerns	Some concerns	Low	Moderate
Shi 2022	Yes	Low	Low	Low	Low	Low	Low	Low	Low
Unisa 2022	No	NA	NA	NA	NA	NA	NA	NA	Critical
Wondemagegn 2022	Yes	Low	Low	Low	Low	Some concerns	Low	Low	Moderate
Wu 2022	Yes	Low	Low	Low	Low	Some concerns	High	Low	Serious
Xiong 2022	Yes	Low	Low	Low	Low	Low	Low	Low	Low
Xu 2022	Yes	Low	Low	Low	Low	Low	Low	Low	Low
Zhang 2022	Yes	Low	Low	Low	Low	Some concerns	Low	Low	Moderate
Zheng 2022	Yes	Low	Low	Some concerns	Low	Some concerns	High	Low	Moderate
Zhu 2022	Yes	Low	Low	Low	Low	Some concerns	Low	Low	Moderate
Abioye 2023	Yes	Low	Low	Some concerns	Low	Low	Low	Low	Moderate
Eldin 2023	Yes	Low	Low	Low	Low	Some concerns	Some concerns	Low	Moderate
Gudipally 2023	Yes	Low	Low	Low	Low	Some concerns	Some concerns	Low	Moderate
Yu 2023	Yes	Low	Low	Low	Low	Some concerns	Some concerns	Low	Moderate

[1] D1: bias (due to confounding) in the estimated effect of exposure on the outcome; D2: bias (arising from measurement of exposure) in the estimated effect of exposure on the outcome; D3: bias due to the selection of participants into the study; D4: bias due to post-exposure interventions; D5: bias due to missing data; D6: bias arising from measurement of outcomes; D7: bias due to selection of the reported result. RoB, risk of bias assessment.

[2] Judged based on whether any attempt was made to control for confounding, whether there is sufficient potential for confounding that an unadjusted result should not be considered further, whether the method of measuring exposure was inappropriate, and whether the method of measuring outcome was inappropriate.

Supplemental Table 11. GRADE assessments for the certainty of evidence.

Exposure/intervention	Comparator	Outcome	Study design	Risk of bias	Inconsistency	Indirectness	Imprecision	Certainty of evidence
MMS	Control	Miscarriage	RCTs	Not serious	Not serious	Not serious	Serious	Moderate
MMS	Control	Stillbirth	RCTs	Not serious	Serious	Not serious	Serious	Low
Any anemia	No anemia	Miscarriage	Observational studies	Very serious	Serious	Not serious	Serious	Very low
Any anemia	No anemia	Stillbirth	Observational studies	Serious	Very serious	Not serious	Not serious	Very low
Mild anemia	No anemia	Stillbirth	Observational studies	Very serious	Very serious	Not serious	Serious	Very low
Moderate anemia	No anemia	Stillbirth	Observational studies	Serious	Very serious	Not serious	Not serious	Very low
Preconceptional obesity	Preconceptional normal weight	Biochemical pregnancy	Observational studies	Serious	Serious	Not serious	Serious	Very low
Preconceptional obesity	Preconceptional normal weight	Clinical pregnancy	Observational studies	Serious	Serious	Not serious	Not serious	Very low
Preconceptional obesity	Preconceptional normal weight	Early pregnancy loss	Observational studies	Serious	Not serious	Not serious	Not serious	Very low
Preconceptional obesity	Preconceptional normal weight	Implantation rate	Observational studies	Very serious	Serious	Not serious	Not serious	Very low
Preconceptional obesity	Preconceptional normal weight	Live birth rate	Observational studies	Serious	Serious	Not serious	Serious	Very low
Preconceptional obesity	Preconceptional normal weight	Miscarriage	Observational studies	Serious	Serious	Not serious	Not serious	Very low
Preconceptional obesity	Preconceptional normal weight	Stillbirth	Observational studies	Serious	Not serious	Not serious	Not serious	Very low
Preconceptional overweight	Preconceptional normal weight	Biochemical pregnancy	Observational studies	Serious	Not serious	Not serious	Not serious	Very low
Preconceptional overweight	Preconceptional normal weight	Clinical pregnancy	Observational studies	Serious	Not serious	Not serious	Not serious	Very low
Preconceptional overweight	Preconceptional normal weight	Early pregnancy loss	Observational studies	Serious	Not serious	Not serious	Not serious	Very low
Preconceptional overweight	Preconceptional normal weight	Implantation rate	Observational studies	Serious	Not serious	Not serious	Not serious	Very low
Preconceptional overweight	Preconceptional normal weight	Live birth rate	Observational studies	Serious	Not serious	Not serious	Not serious	Very low
Preconceptional overweight	Preconceptional normal weight	Miscarriage	Observational studies	Serious	Not serious	Not serious	Not serious	Very low
Preconceptional overweight	Preconceptional normal weight	Stillbirth	Observational studies	Serious	Not serious	Not serious	Not serious	Very low
Preconceptional underweight	Preconceptional normal weight	Biochemical pregnancy	Observational studies	Serious	Not serious	Not serious	Not serious	Very low
Preconceptional underweight	Preconceptional normal weight	Clinical pregnancy	Observational studies	Serious	Not serious	Not serious	Not serious	Very low
Preconceptional underweight	Preconceptional normal weight	Early pregnancy loss	Observational studies	Serious	Not serious	Not serious	Not serious	Very low
Preconceptional underweight	Preconceptional normal weight	Implantation rate	Observational studies	Serious	Not serious	Not serious	Not serious	Very low
Preconceptional underweight	Preconceptional normal weight	Live birth rate	Observational studies	Serious	Serious	Not serious	Not serious	Very low
Preconceptional underweight	Preconceptional normal weight	Miscarriage	Observational studies	Serious	Serious	Not serious	Not serious	Very low
Preconceptional underweight	Preconceptional normal weight	Stillbirth	Observational studies	Very serious	Not serious	Not serious	Not serious	Very low
Obesity during early pregnancy	Normal weight during early pregnancy	Stillbirth	Observational studies	Very serious	Not serious	Not serious	Serious	Very low
Overweight during early pregnancy	Normal weight during early pregnancy	Stillbirth	Observational studies	Very serious	Serious	Not serious	Very serious	Very low
Underweight during early pregnancy	Normal weight during early pregnancy	Stillbirth	Observational studies	Very serious	Not serious	Not serious	Serious	Very low

MMS, multiple micronutrient supplementation. RCT, randomized controlled trial.

REFERENCES

1. Khan AD, Schroeder DG, Martorell R, et al. Age at menarche and nutritional supplementation. *J Nutr* 1995;125(4 Suppl):1090s-96s. doi: 10.1093/jn/125.suppl_4.1090S
2. Ceesay SM, Prentice AM, Cole TJ, et al. Effects on birth weight and perinatal mortality of maternal dietary supplements in rural Gambia: 5 year randomised controlled trial. *Bmj* 1997;315(7111):786-90. doi: 10.1136/bmj.315.7111.786
3. Fawzi WW, Msamanga GI, Spiegelman D, et al. Randomised trial of effects of vitamin supplements on pregnancy outcomes and T cell counts in HIV-1-infected women in Tanzania. *Lancet* 1998;351(9114):1477-82. doi: 10.1016/s0140-6736(98)04197-x
4. Katz J, West KP, Jr., Khatry SK, et al. Maternal low-dose vitamin A or beta-carotene supplementation has no effect on fetal loss and early infant mortality: a randomized cluster trial in Nepal. *Am J Clin Nutr* 2000;71(6):1570-6. doi: 10.1093/ajcn/71.6.1570
5. Christian P, West KP, Khatry SK, et al. Effects of maternal micronutrient supplementation on fetal loss and infant mortality: a cluster-randomized trial in Nepal. *Am J Clin Nutr* 2003;78(6):1194-202. doi: 10.1093/ajcn/78.6.1194
6. Friis H, Gomo E, Nyazema N, et al. Effect of multimicronutrient supplementation on gestational length and birth size: a randomized, placebo-controlled, double-blind effectiveness trial in Zimbabwe. *Am J Clin Nutr* 2004;80(1):178-84. doi: 10.1093/ajcn/80.1.178
7. Fawzi WW, Villamor E, Msamanga GI, et al. Trial of zinc supplements in relation to pregnancy outcomes, hematologic indicators, and T cell counts among HIV-1-infected women in Tanzania. *Am J Clin Nutr* 2005;81(1):161-7. doi: 10.1093/ajcn/81.1.161
8. Kaestel P, Michaelsen KF, Aaby P, et al. Effects of prenatal multimicronutrient supplements on birth weight and perinatal mortality: a randomised, controlled trial in Guinea-Bissau. *Eur J Clin Nutr* 2005;59(9):1081-9. doi: 10.1038/sj.ejcn.1602215
9. Osrin D, Vaidya A, Shrestha Y, et al. Effects of antenatal multiple micronutrient supplementation on birthweight and gestational duration in Nepal: double-blind, randomised controlled trial. *Lancet* 2005;365(9463):955-62. doi: 10.1016/s0140-6736(05)71084-9
10. Qublan HS, Yannakoula EK, Al-Qudah MA, et al. Dietary intervention versus metformin to improve the reproductive outcome in women with polycystic ovary syndrome. A prospective comparative study. *Saudi Med J* 2007;28(11):1694-9.
11. Fawzi WW, Msamanga GI, Urassa W, et al. Vitamins and perinatal outcomes among HIV-negative women in Tanzania. *N Engl J Med* 2007;356(14):1423-31. doi: 10.1056/NEJMoa064868
12. Zagré NM, Desplats G, Adou P, et al. Prenatal multiple micronutrient supplementation has greater impact on birthweight than supplementation with iron and folic acid: a cluster-randomized, double-blind, controlled programmatic study in rural Niger. *Food Nutr Bull* 2007;28(3):317-27. doi: 10.1177/156482650702800308
13. Kupka R, Mugusi F, Aboud S, et al. Randomized, double-blind, placebo-controlled trial of selenium supplements among HIV-infected pregnant women in Tanzania: effects on maternal and child outcomes. *Am J Clin Nutr* 2008;87(6):1802-8. doi: 10.1093/ajcn/87.6.1802
14. Shankar AH, Jahari AB, Sebayang SK, et al. Effect of maternal multiple micronutrient supplementation on fetal loss and infant death in Indonesia: a double-blind cluster-randomised trial. *Lancet* 2008;371(9608):215-27. doi: 10.1016/s0140-6736(08)60133-6
15. Zeng L, Dibley MJ, Cheng Y, et al. Impact of micronutrient supplementation during pregnancy on birth weight, duration of gestation, and perinatal mortality in rural western China: double blind cluster randomised controlled trial. *Bmj* 2008;337:a2001. doi: 10.1136/bmj.a2001 [published Online First: 20081107]
16. Roberfroid D, Huybregts L, Lanou H, et al. Effects of maternal multiple micronutrient supplementation on fetal growth: a double-blind randomized controlled trial in rural Burkina Faso. *Am J Clin Nutr* 2008;88(5):1330-40. doi: 10.3945/ajcn.2008.26296
17. Sunawang, Utomo B, Hidayat A, et al. Preventing low birthweight through maternal multiple micronutrient supplementation: a cluster-randomized, controlled trial in Indramayu, West Java. *Food Nutr Bull* 2009;30(4 Suppl):S488-95. doi: 10.1177/15648265090304s403

18. Bhutta ZA, Rizvi A, Raza F, et al. A comparative evaluation of multiple micronutrient and iron-folic acid supplementation during pregnancy in Pakistan: impact on pregnancy outcomes. *Food Nutr Bull* 2009;30(4 Suppl):S496-505. doi: 10.1177/15648265090304s404
19. Aminisani N, Ehdaivand F, Shamshirgaran S, et al. Zinc supplementation during pregnancy: a randomized controlled trial. *Iranian Journal of Pharmacology & Therapeutics* 2009;8(2):67-71.
20. Ramakrishnan U, Stein AD, Parra-Cabrera S, et al. Effects of docosahexaenoic acid supplementation during pregnancy on gestational age and size at birth: randomized, double-blind, placebo-controlled trial in Mexico. *Food Nutr Bull* 2010;31(2 Suppl):S108-16. doi: 10.1177/15648265100312s203
21. Kawai K, Kupka R, Mugusi F, et al. A randomized trial to determine the optimal dosage of multivitamin supplements to reduce adverse pregnancy outcomes among HIV-infected women in Tanzania. *Am J Clin Nutr* 2010;91(2):391-7. doi: 10.3945/ajcn.2009.28483 [published Online First: 20091125]
22. West KP, Jr., Christian P, Labrique AB, et al. Effects of vitamin A or beta carotene supplementation on pregnancy-related mortality and infant mortality in rural Bangladesh: a cluster randomized trial. *Jama* 2011;305(19):1986-95. doi: 10.1001/jama.2011.656
23. Persson L, Arifeen S, Ekström EC, et al. Effects of prenatal micronutrient and early food supplementation on maternal hemoglobin, birth weight, and infant mortality among children in Bangladesh: the MINIMat randomized trial. *Jama* 2012;307(19):2050-9. doi: 10.1001/jama.2012.4061
24. Vila-Nova C, Wehby GL, Queirós FC, et al. Periconceptional use of folic acid and risk of miscarriage - findings of the Oral Cleft Prevention Program in Brazil. *J Perinat Med* 2013;41(4):461-6. doi: 10.1515/jpm-2012-0173
25. Wang YF, Pei LJ, Song XM, et al. Impact of periconceptional multi-micronutrient supplementation on gestation: a population-based study. *Biomed Environ Sci* 2013;26(1):23-31. doi: 10.3967/0895-3988.2013.01.003
26. Liu JM, Mei Z, Ye R, et al. Micronutrient supplementation and pregnancy outcomes: double-blind randomized controlled trial in China. *JAMA Intern Med* 2013;173(4):276-82. doi: 10.1001/jamainternmed.2013.1632
27. Kiondo P, Wamuyu-Maina G, Wandabwa J, et al. The effects of vitamin C supplementation on pre-eclampsia in Mulago Hospital, Kampala, Uganda: a randomized placebo controlled clinical trial. *BMC Pregnancy Childbirth* 2014;14:283. doi: 10.1186/1471-2393-14-283 [published Online First: 20140821]
28. West KP, Jr., Shamim AA, Mehra S, et al. Effect of maternal multiple micronutrient vs iron-folic acid supplementation on infant mortality and adverse birth outcomes in rural Bangladesh: the JiVitA-3 randomized trial. *Jama* 2014;312(24):2649-58. doi: 10.1001/jama.2014.16819
29. Aflatoonian A, Arabjahvani F, Eftekhar M, et al. Effect of vitamin D insufficiency treatment on fertility outcomes in frozen-thawed embryo transfer cycles: A randomized clinical trial. *Iran J Reprod Med* 2014;12(9):595-600.
30. Hekmatdoost A, Vahid F, Yari Z, et al. Methyltetrahydrofolate vs Folic Acid Supplementation in Idiopathic Recurrent Miscarriage with Respect to Methylenetetrahydrofolate Reductase C677T and A1298C Polymorphisms: A Randomized Controlled Trial. *PLoS One* 2015;10(12):e0143569. doi: 10.1371/journal.pone.0143569 [published Online First: 20151202]
31. Becker GF, Passos EP, Moulin CC. Short-term effects of a hypocaloric diet with low glycemic index and low glycemic load on body adiposity, metabolic variables, ghrelin, leptin, and pregnancy rate in overweight and obese infertile women: a randomized controlled trial. *Am J Clin Nutr* 2015;102(6):1365-72. doi: 10.3945/ajcn.115.117200 [published Online First: 20151111]
32. Anees M, Anis RA, Yousaf S, et al. Effect of maternal iodine supplementation on thyroid function and birth outcome in goiter endemic areas. *Curr Med Res Opin* 2015;31(4):667-74. doi: 10.1185/03007995.2015.1011779 [published Online First: 20150213]
33. Ashorn P, Alho L, Ashorn U, et al. The impact of lipid-based nutrient supplement provision to pregnant women on newborn size in rural Malawi: a randomized controlled trial. *Am J Clin Nutr* 2015;101(2):387-97. doi: 10.3945/ajcn.114.088617 [published Online First: 20141210]
34. Adu-Afarwuah S, Lartey A, Okronipa H, et al. Lipid-based nutrient supplement increases the birth size of infants of primiparous women in Ghana. *Am J Clin Nutr* 2015;101(4):835-46. doi: 10.3945/ajcn.114.091546 [published Online First: 20150211]
35. Zahran KM, Abd Elaal DEM, Kamel HS, et al. A combination treatment of folic acid, aspirin, doxycycline and progesterone for women with recurrent early pregnancy loss; hospital based study. *Middle East Fertility Society Journal* 2016;21(1):22-26.

36. Sayyah-Melli M, Ghorbanihaghjo A, Alizadeh M, et al. The Effect of High Dose Folic Acid throughout Pregnancy on Homocysteine (Hcy) Concentration and Pre-Eclampsia: A Randomized Clinical Trial. *PLoS One* 2016;11(5):e0154400. doi: 10.1371/journal.pone.0154400 [published Online First: 20160511]
37. Zahiri Sorouri Z, Sadeghi H, Pourmarzi D. The effect of zinc supplementation on pregnancy outcome: a randomized controlled trial. *J Matern Fetal Neonatal Med* 2016;29(13):2194-8. doi: 10.3109/14767058.2015.1079615 [published Online First: 20150912]
38. Al-Eisa E, Gabr SA, Alghadir AH. Effects of supervised aerobic training on the levels of anti-Mullerian hormone and adiposity measures in women with normo-ovulatory and polycystic ovary syndrome. *J Pak Med Assoc* 2017;67(4):499-507.
39. Al-Alousi TA, Aziz AA, Al-Allak MMA, et al. The effect of omega-3 on the number of retrieved ova, fertilization rate, and embryo grading in subfertile women undergoing intracytoplasmic sperm injection. *Biomedical and Pharmacology Journal* 2018;11(4):2221-32.
40. Charkamyani F, Hosseinkhani A, Neisani Samani L, et al. Reducing the Adverse Maternal and Fetal Outcomes in IVF Women by Exercise Interventions During Pregnancy. *Res Q Exerc Sport* 2019;90(4):589-99. doi: 10.1080/02701367.2019.1639601 [published Online First: 20190809]
41. Kadoura S, Alhalabi M, Nattouf AH. Effect of Calcium and Vitamin D Supplements as an Adjuvant Therapy to Metformin on Menstrual Cycle Abnormalities, Hormonal Profile, and IGF-1 System in Polycystic Ovary Syndrome Patients: A Randomized, Placebo-Controlled Clinical Trial. *Adv Pharmacol Sci* 2019;2019:9680390. doi: 10.1155/2019/9680390 [published Online First: 20190701]
42. de Araújo CAL, Ray JG, Figueiroa JN, et al. BRAzil magnesium (BRAMAG) trial: a double-masked randomized clinical trial of oral magnesium supplementation in pregnancy. *BMC Pregnancy Childbirth* 2020;20(1):234. doi: 10.1186/s12884-020-02935-7 [published Online First: 20200421]
43. Al-Bayyari N, Al-Domi H, Zayed F, et al. Androgens and hirsutism score of overweight women with polycystic ovary syndrome improved after vitamin D treatment: A randomized placebo controlled clinical trial. *Clin Nutr* 2021;40(3):870-78. doi: 10.1016/j.clnu.2020.09.024 [published Online First: 20200924]
44. Nausheen S, Habib A, Bhura M, et al. Impact evaluation of the efficacy of different doses of vitamin D supplementation during pregnancy on pregnancy and birth outcomes: a randomised, controlled, dose comparison trial in Pakistan. *BMJ Nutr Prev Health* 2021;4(2):425-34. doi: 10.1136/bmjnph-2021-000304 [published Online First: 20210927]
45. Sudfeld CR, Manji KP, Muhihi A, et al. Vitamin D3 supplementation during pregnancy and lactation for women living with HIV in Tanzania: A randomized controlled trial. *PLoS Med* 2022;19(4):e1003973. doi: 10.1371/journal.pmed.1003973 [published Online First: 20220415]
46. de Kok B, Toe LC, Hanley-Cook G, et al. Prenatal fortified balanced energy-protein supplementation and birth outcomes in rural Burkina Faso: A randomized controlled efficacy trial. *PLoS Med* 2022;19(5):e1004002. doi: 10.1371/journal.pmed.1004002 [published Online First: 20220513]
47. Ilboudo B, Savadogo LGB, Traoré I, et al. Effect of personalized home-based support for pregnant women on pregnancy outcomes: a cluster randomized trial. *J Public Health Afr* 2022;13(3):1939. doi: 10.4081/jphia.2022.1939 [published Online First: 20220912]
48. Pharoah PO, Ellis SM, Ekins RP, et al. Maternal thyroid function, iodine deficiency and fetal development. *Clin Endocrinol (Oxf)* 1976;5(2):159-66. doi: 10.1111/j.1365-2265.1976.tb02827.x
49. Popkin BM, Guilkey DK, Akin JS, et al. Nutrition, lactation, and birth spacing in Filipino women. *Demography* 1993;30(3):333-52.
50. Kurz KM, Habicht JP, Rasmussen KM, et al. Effects of maternal nutritional status and maternal energy supplementation on length of postpartum amenorrhea among Guatemalan women. *Am J Clin Nutr* 1993;58(5):636-42. doi: 10.1093/ajcn/58.5.636
51. Khan AD, Schroeder DG, Martorell R, et al. Early childhood determinants of age at menarche in rural guatemala. *Am J Hum Biol* 1996;8(6):717-23. doi: 10.1002/(sici)1520-6300(1996)8:6<717::Aid-ajhb3>3.0.Co;2-q
52. Onadeko MO, Avokey F, Lawoyin TO. Observations of stillbirths, birthweight and maternal haemoglobin in teenage pregnancy in Ibadan, Nigeria. *Afr J Med Med Sci* 1996;25(1):81-6.
53. Simondon KB, Simondon F, Simon I, et al. Preschool stunting, age at menarche and adolescent height: a longitudinal study in rural Senegal. *Eur J Clin Nutr* 1998;52(6):412-8. doi: 10.1038/sj.ejcn.1600577

54. Agarwal DK, Agarwal A, Singh M, et al. Pregnancy wastage in rural Varanasi: relationship with maternal nutrition and sociodemographic characteristics. *Indian Pediatr* 1998;35(11):1071-9.
55. Ramakrishnan U, Barnhart H, Schroeder DG, et al. Early childhood nutrition, education and fertility milestones in Guatemala. *J Nutr* 1999;129(12):2196-202. doi: 10.1093/jn/129.12.2196
56. Adair LS. Size at birth predicts age at menarche. *Pediatrics* 2001;107(4):E59. doi: 10.1542/peds.107.4.e59
57. Gindler J, Li Z, Berry RJ, et al. Folic acid supplements during pregnancy and risk of miscarriage. *Lancet* 2001;358(9284):796-800. doi: 10.1016/s0140-6736(01)05969-4
58. Khan MM. Effect of maternal anaemia on fetal parameters. *J Ayub Med Coll Abbottabad* 2001;13(2):38-41.
59. Osman NB, Challis K, Cotiro M, et al. Perinatal outcome in an obstetric cohort of Mozambican women. *J Trop Pediatr* 2001;47(1):30-8. doi: 10.1093/tropej/47.1.30
60. Chalumeau M, Bouvier-Colle MH, Breart G. Can clinical risk factors for late stillbirth in West Africa be detected during antenatal care or only during labour? *Int J Epidemiol* 2002;31(3):661-8. doi: 10.1093/ije/31.3.661
61. Villamor E, Dreyfuss ML, Baylín A, et al. Weight loss during pregnancy is associated with adverse pregnancy outcomes among HIV-1 infected women. *J Nutr* 2004;134(6):1424-31. doi: 10.1093/jn/134.6.1424
62. Lone FW, Qureshi RN, Emanuel F. Maternal anaemia and its impact on perinatal outcome. *Trop Med Int Health* 2004;9(4):486-90. doi: 10.1111/j.1365-3156.2004.01222.x
63. Kupka R, Garland M, Msamanga G, et al. Selenium status, pregnancy outcomes, and mother-to-child transmission of HIV-1. *J Acquir Immune Defic Syndr* 2005;39(2):203-10.
64. Watson-Jones D, Weiss HA, Changalucha JM, et al. Adverse birth outcomes in United Republic of Tanzania--impact and prevention of maternal risk factors. *Bull World Health Organ* 2007;85(1):9-18. doi: 10.2471/blt.06.033258
65. Ronnenberg AG, Venners SA, Xu X, et al. Preconception B-vitamin and homocysteine status, conception, and early pregnancy loss. *Am J Epidemiol* 2007;166(3):304-12. doi: 10.1093/aje/kwm078 [published Online First: 20070502]
66. Prabhakar S, Sahota P, Kharbanda PS, et al. Sodium valproate, hyperandrogenism and altered ovarian function in Indian women with epilepsy: a prospective study. *Epilepsia* 2007;48(7):1371-7. doi: 10.1111/j.1528-1167.2007.01100.x [published Online First: 20070418]
67. Bosch AM, Willekens FJ, Baqui AH, et al. Association between age at menarche and early-life nutritional status in rural Bangladesh. *J Biosoc Sci* 2008;40(2):223-37. doi: 10.1017/s0021932007002490 [published Online First: 20071024]
68. Mehta S, Manji KP, Young AM, et al. Nutritional indicators of adverse pregnancy outcomes and mother-to-child transmission of HIV among HIV-infected women. *Am J Clin Nutr* 2008;87(6):1639-49. doi: 10.1093/ajcn/87.6.1639
69. Hauger MS, Gibbons L, Vik T, et al. Prepregnancy weight status and the risk of adverse pregnancy outcome. *Acta Obstet Gynecol Scand* 2008;87(9):953-9. doi: 10.1080/00016340802303349
70. Zhang Q, Ananth CV, Rhoads GG, et al. The impact of maternal anemia on perinatal mortality: a population-based, prospective cohort study in China. *Ann Epidemiol* 2009;19(11):793-9. doi: 10.1016/j.annepidem.2009.06.002 [published Online First: 20090803]
71. Chumak EL, Grjibovski AM. Anemia in pregnancy and its association with pregnancy outcomes in the Arctic Russian town of Monchegorsk, 1973-2002. *Int J Circumpolar Health* 2010;69(3):265-77. doi: 10.3402/ijch.v69i3.17603 [published Online First: 20100517]
72. Mesa JM, Araújo C, Horta BL, et al. Growth patterns in early childhood and the onset of menarche before age twelve. *Rev Saude Publica* 2010;44(2):249-60. doi: 10.1590/s0034-89102010000200004
73. Guerra-Shinohara EM, Pereira PM, Kubota AM, et al. Increased MMA concentration and body mass index are associated with spontaneous abortion in Brazilian women: a pilot study. *Clin Chim Acta* 2010;411(5-6):423-7. doi: 10.1016/j.cca.2009.12.014 [published Online First: 20091228]
74. Zhang D, Zhu Y, Gao H, et al. Overweight and obesity negatively affect the outcomes of ovarian stimulation and in vitro fertilisation: a cohort study of 2628 Chinese women. *Gynecol Endocrinol* 2010;26(5):325-32. doi: 10.3109/09513591003632100
75. Li Y, Yang D, Zhang Q. Impact of overweight and underweight on IVF treatment in Chinese women. *Gynecol Endocrinol* 2010;26(6):416-22. doi: 10.3109/09513591003632118

76. Abeysena C, Jayawardana P, de ASR. Maternal haemoglobin level at booking visit and its effect on adverse pregnancy outcome. *Aust N Z J Obstet Gynaecol* 2010;50(5):423-7. doi: 10.1111/j.1479-828X.2010.01220.x [published Online First: 20100901]
77. Villamor E, Marin C, Mora-Plazas M, et al. Vitamin D deficiency and age at menarche: a prospective study. *Am J Clin Nutr* 2011;94(4):1020-5. doi: 10.3945/ajcn.111.018168 [published Online First: 20110810]
78. Belachew T, Hadley C, Lindstrom D, et al. Food insecurity and age at menarche among adolescent girls in Jimma Zone Southwest Ethiopia: a longitudinal study. *Reprod Biol Endocrinol* 2011;9:125. doi: 10.1186/1477-7827-9-125 [published Online First: 20110913]
79. Stringer EM, Vwalika B, Killam WP, et al. Determinants of stillbirth in Zambia. *Obstet Gynecol* 2011;117(5):1151-59. doi: 10.1097/AOG.0b013e3182167627
80. Chumak EL, Grjibovski AM. Association between different levels of hemoglobin in pregnancy and pregnancy outcomes: a registry-based study in Northwest Russia. *Int J Circumpolar Health* 2011;70(5):457-9. doi: 10.3402/ijch.v70i5.17851 [published Online First: 20111017]
81. Gonzales GF, Tapia V, Gasco M, et al. Maternal hemoglobin concentration and adverse pregnancy outcomes at low and moderate altitudes in Peru. *J Matern Fetal Neonatal Med* 2012;25(7):1105-10. doi: 10.3109/14767058.2011.623200 [published Online First: 20111017]
82. Reyes E, Martínez N, Parra A, et al. Early intensive obstetric and medical nutrition care is associated with decreased prepregnancy obesity impact on perinatal outcomes. *Gynecol Obstet Invest* 2012;73(1):75-81. doi: 10.1159/000329899 [published Online First: 20110902]
83. Young S, Murray K, Mwesigwa J, et al. Maternal nutritional status predicts adverse birth outcomes among HIV-infected rural Ugandan women receiving combination antiretroviral therapy. *PLoS One* 2012;7(8):e41934. doi: 10.1371/journal.pone.0041934 [published Online First: 20120807]
84. Neumann CG, Oace SM, Chaparro MP, et al. Low vitamin B12 intake during pregnancy and lactation and low breastmilk vitamin 12 content in rural Kenyan women consuming predominantly maize diets. *Food Nutr Bull* 2013;34(2):151-9. doi: 10.1177/156482651303400204
85. Malhotra N, Bahadur A, Singh N, et al. Does obesity compromise ovarian reserve markers? A clinician's perspective. *Arch Gynecol Obstet* 2013;287(1):161-6. doi: 10.1007/s00404-012-2528-7 [published Online First: 20120829]
86. Kumari R, Mengi V, Kumar D. Maternal risk factors & pregnancy wastage in a rural population of Jammu District. *JK Science* 2013;15(2):82.
87. Sharma R. Prospective Study of Effect of Body Weight on in vitro Fertilization Outcome in Reproductive Age Group. *International journal of infertility and fetal medicine* 2014;5(2):58-63. doi: 10.5005/jp-journals-10016-1082
88. Huang K, Liao X, Dong X, et al. Effect of overweight/obesity on IVF-ET outcomes in chinese patients with polycystic ovary syndrome. *Int J Clin Exp Med* 2014;7(12):5872-6. [published Online First: 20141215]
89. Zhou J, Su L, Liu M, et al. Associations between 25-hydroxyvitamin D levels and pregnancy outcomes: a prospective observational study in southern China. *Eur J Clin Nutr* 2014;68(8):925-30. doi: 10.1038/ejcn.2014.99 [published Online First: 20140528]
90. Ouyang F, Longnecker MP, Venners SA, et al. Preconception serum 1,1,1-trichloro-2,2,bis(p-chlorophenyl)ethane and B-vitamin status: independent and joint effects on women's reproductive outcomes. *Am J Clin Nutr* 2014;100(6):1470-8. doi: 10.3945/ajcn.114.088377 [published Online First: 20141022]
91. Cung TG, Paus AS, Aghbar A, et al. Stillbirths at a hospital in Nablus, 2010: a cohort study. *Glob Health Action* 2014;7:25222. doi: 10.3402/gha.v7.25222 [published Online First: 20140905]
92. Meng Q, Ren A, Zhang L, et al. Incidence of infertility and risk factors of impaired fecundity among newly married couples in a Chinese population. *Reprod Biomed Online* 2015;30(1):92-100. doi: 10.1016/j.rbmo.2014.10.002 [published Online First: 20141013]
93. Zhai L, Liu J, Zhao J, et al. Association of Obesity with Onset of Puberty and Sex Hormones in Chinese Girls: A 4-Year Longitudinal Study. *PLoS One* 2015;10(8):e0134656. doi: 10.1371/journal.pone.0134656 [published Online First: 20150806]
94. Salgin B, Norris SA, Prentice P, et al. Even transient rapid infancy weight gain is associated with higher BMI in young adults and earlier menarche. *Int J Obes (Lond)* 2015;39(6):939-44. doi: 10.1038/ijo.2015.25 [published Online First: 20150316]

95. Awan S, Bibi S, Makhdoom A, et al. Adverse fetomaternal outcome among pregnant overweight women. *Pak J Med Sci* 2015;31(2):383-7. doi: 10.12669/pjms.312.6530
96. Shen PJ, Gong B, Xu FY, et al. Four trace elements in pregnant women and their relationships with adverse pregnancy outcomes. *Eur Rev Med Pharmacol Sci* 2015;19(24):4690-7.
97. Jansen EC, Marín C, Mora-Plazas M, et al. Higher Childhood Red Meat Intake Frequency Is Associated with Earlier Age at Menarche. *J Nutr* 2015;146(4):792-98. doi: 10.3945/jn.115.226456
98. Shen X-F, Liu X, Zhang Y-H, et al. Obesity impaired oocyte maturation and embryo implantation rate in Chinese women without polycystic ovary syndrome undergoing in vitro fertilization-embryo transfer. *Int J Clin Exp Med* 2016;9(10):19995-20001.
99. Zhou H, Liu Y, Liu L, et al. Maternal pre-pregnancy risk factors for miscarriage from a prevention perspective: a cohort study in China. *Eur J Obstet Gynecol Reprod Biol* 2016;206:57-63. doi: 10.1016/j.ejogrb.2016.07.514 [published Online First: 20160812]
100. Pan Y, Zhang S, Wang Q, et al. Investigating the association between prepregnancy body mass index and adverse pregnancy outcomes: a large cohort study of 536 098 Chinese pregnant women in rural China. *BMJ Open* 2016;6(6):e011227. doi: 10.1136/bmjopen-2016-011227 [published Online First: 20160720]
101. Zerfu TA, Umeta M, Baye K. Dietary diversity during pregnancy is associated with reduced risk of maternal anemia, preterm delivery, and low birth weight in a prospective cohort study in rural Ethiopia. *Am J Clin Nutr* 2016;103(6):1482-8. doi: 10.3945/ajcn.115.116798 [published Online First: 20160511]
102. Aydin BK, Devecioglu E, Kadioglu A, et al. The relationship between infancy growth rate and the onset of puberty in both genders. *Pediatr Res* 2017;82(6):940-46. doi: 10.1038/pr.2017.194 [published Online First: 20170913]
103. Ahmed HO. Improvement in Fertility After Bariatric Surgery in Obese Females with Polycystic Ovarian Syndrome: Based on Four Years of Experience in Two Centers in Sulaimani Governorate, Kurdistan Region/Iraq. *Bariatric surgical practice and patient care* 2017;12(4):162-69. doi: 10.1089/bari.2017.0041
104. Villamor E, Marín C, Mora-Plazas M, et al. Micronutrient status in middle childhood and age at menarche: results from the Bogotá School Children Cohort. *Br J Nutr* 2017;118(12):1097-105. doi: 10.1017/s0007114517003130 [published Online First: 20171204]
105. Narasati S, Riayati O, Wiweko B, et al. Effect of Female Body Mass Index on Clinical Pregnancy Rate After In Vitro Fertilization. *Advanced science letters* 2017;23(7):7009-11. doi: 10.1166/asl.2017.9455
106. MacKenna A, Schwarze JE, Crosby JA, et al. Outcome of assisted reproductive technology in overweight and obese women. *JBRA Assist Reprod* 2017;21(2):79-83. doi: 10.5935/1518-0557.20170020 [published Online First: 20170601]
107. Kumar DSS, Keerthinmayee K. A Study on the Effects of Anaemia on Pregnancy Outcome in an Urban Health Centre Practice Area of Tirupati. *Indian journal of public health research and development* 2017;8(1):215. doi: 10.5958/0976-5506.2017.00044.4
108. Suryanarayana R, Chandrappa M, Santhuram AN, et al. Prospective study on prevalence of anemia of pregnant women and its outcome: A community based study. *J Family Med Prim Care* 2017;6(4):739-43. doi: 10.4103/jfmpc.jfmpc_33_17
109. Sheng Y, Lu G, Liu J, et al. Effect of body mass index on the outcomes of controlled ovarian hyperstimulation in Chinese women with polycystic ovary syndrome: a multicenter, prospective, observational study. *J Assist Reprod Genet* 2017;34(1):61-70. doi: 10.1007/s10815-016-0830-1 [published Online First: 20161105]
110. Cai J, Liu L, Zhang J, et al. Low body mass index compromises live birth rate in fresh transfer in vitro fertilization cycles: a retrospective study in a Chinese population. *Fertil Steril* 2017;107(2):422-29.e2. doi: 10.1016/j.fertnstert.2016.10.029 [published Online First: 20161122]
111. Mosha D, Liu E, Hertzmark E, et al. Dietary iron and calcium intakes during pregnancy are associated with lower risk of prematurity, stillbirth and neonatal mortality among women in Tanzania. *Public Health Nutr* 2017;20(4):678-86. doi: 10.1017/s1368980016002809 [published Online First: 20161107]
112. Darling AM, McDonald CR, Urassa WS, et al. Maternal Dietary L-Arginine and Adverse Birth Outcomes in Dar es Salaam, Tanzania. *Am J Epidemiol* 2017;186(5):603-11. doi: 10.1093/aje/kwx080
113. Kyweluk MA, Georgiev AV, Borja JB, et al. Menarcheal timing is accelerated by favorable nutrition but unrelated to developmental cues of mortality or familial instability in Cebu, Philippines. *Evolution and human behavior* 2018;39(1):76-81. doi: 10.1016/j.evolhumbehav.2017.10.002

114. Jansen EC, Zhou L, Perng W, et al. Vegetables and lean proteins-based and processed meats and refined grains -based dietary patterns in early childhood are associated with pubertal timing in a sex-specific manner: a prospective study of children from Mexico City. *Nutr Res* 2018;56:41-50. doi: 10.1016/j.nutres.2018.04.021 [published Online First: 20180503]
115. Arefi S, Khalili G, Iranmanesh H, et al. Is the ovarian reserve influenced by vitamin D deficiency and the dress code in an infertile Iranian population? *J Ovarian Res* 2018;11(1):62. doi: 10.1186/s13048-018-0435-7 [published Online First: 20180724]
116. Li W, Liu Q, Deng X, et al. Association of prepubertal obesity with pubertal development in Chinese girls and boys: A longitudinal study. *Am J Hum Biol* 2018;30(6):e23195. doi: 10.1002/ajhb.23195 [published Online First: 20181102]
117. Pan X-m, Lin Z, Li N, et al. Effects of body mass index on the outcomes of in vitro fertilization in Chinese patients with polycystic ovary syndrome: a retrospective cohort study. *Journal of Zhejiang University B Science* 2018;19(6):490-96. doi: 10.1631/jzus.B1800113
118. Huang Y, Li J, Zhang F, et al. Factors affecting the live-birth rate in women with diminished ovarian reserve undergoing IVF-ET. *Arch Gynecol Obstet* 2018;298(5):1017-27. doi: 10.1007/s00404-018-4884-4 [published Online First: 20180919]
119. Zerfu TA, Pinto E, Baye K. Consumption of dairy, fruits and dark green leafy vegetables is associated with lower risk of adverse pregnancy outcomes (APO): a prospective cohort study in rural Ethiopia. *Nutr Diabetes* 2018;8(1):52. doi: 10.1038/s41387-018-0060-y [published Online First: 20180920]
120. Patel A, Prakash AA, Das PK, et al. Maternal anemia and underweight as determinants of pregnancy outcomes: cohort study in eastern rural Maharashtra, India. *BMJ Open* 2018;8(8):e021623. doi: 10.1136/bmjopen-2018-021623 [published Online First: 20180808]
121. Mocking M, Savitri AI, Uiterwaal C, et al. Does body mass index early in pregnancy influence the risk of maternal anaemia? An observational study in Indonesian and Ghanaian women. *BMC Public Health* 2018;18(1):873. doi: 10.1186/s12889-018-5704-2 [published Online First: 20180713]
122. Dhaded SM, Somannavar MS, Jacob JP, et al. Early pregnancy loss in Belagavi, Karnataka, India 2014-2017: a prospective population-based observational study in a low-resource setting. *Reprod Health* 2018;15(Suppl 1):95. doi: 10.1186/s12978-018-0525-4 [published Online First: 20180622]
123. Aurino E, Schott W, Penny ME, et al. Birth weight and prepubertal body size predict menarcheal age in India, Peru, and Vietnam. *Ann N Y Acad Sci* 2017 doi: 10.1111/nyas.13445 [published Online First: 20170928]
124. Miller EM, Khalil M. Iron and fecundity among Tsimane' women of Bolivia. *Evol Med Public Health* 2019;2019(1):111-20. doi: 10.1093/emph/eoz020 [published Online First: 20190704]
125. Zhao J, Hong X, Zhang H, et al. Pre-pregnancy maternal fasting plasma glucose levels in relation to time to pregnancy among the couples attempting first pregnancy. *Hum Reprod* 2019;34(7):1325-33. doi: 10.1093/humrep/dez069
126. Maged AM, Fahmy RM, Rashwan H, et al. Effect of body mass index on the outcome of IVF cycles among patients with poor ovarian response. *Int J Gynaecol Obstet* 2019;144(2):161-66. doi: 10.1002/ijgo.12706 [published Online First: 20181126]
127. Jahangirifar M, Taebi M, Nasr-Esfahani MH, et al. Dietary Patterns and The Outcomes of Assisted Reproductive Techniques in Women with Primary Infertility: A Prospective Cohort Study. *Int J Fertil Steril* 2019;12(4):316-23. doi: 10.22074/ijfs.2019.5373 [published Online First: 20181002]
128. Zhou Y, Li H, Zhang Y, et al. Association of Maternal Obesity in Early Pregnancy with Adverse Pregnancy Outcomes: A Chinese Prospective Cohort Analysis. *Obesity (Silver Spring)* 2019;27(6):1030-36. doi: 10.1002/oby.22478 [published Online First: 20190508]
129. Zhou Y, Li H, Zhang Y, et al. Rate of gestational weight gain and adverse pregnancy outcomes in rural nulliparous women: a prospective cohort analysis from China. *Br J Nutr* 2019;122(3):352-59. doi: 10.1017/s0007114519001247 [published Online First: 20190726]
130. Zhang J, Liu H, Mao X, et al. Effect of body mass index on pregnancy outcomes in a freeze-all policy: an analysis of 22,043 first autologous frozen-thawed embryo transfer cycles in China. *BMC Med* 2019;17(1):114. doi: 10.1186/s12916-019-1354-1 [published Online First: 20190626]

131. Parks S, Hoffman MK, Goudar SS, et al. Maternal anaemia and maternal, fetal, and neonatal outcomes in a prospective cohort study in India and Pakistan. *Bjog* 2019;126(6):737-43. doi: 10.1111/1471-0528.15585 [published Online First: 20190124]
132. Svefors P, Pervin J, Islam Khan A, et al. Stunting, recovery from stunting and puberty development in the MINIMat cohort, Bangladesh. *Acta Paediatr* 2020;109(1):122-33. doi: 10.1111/apa.14929 [published Online First: 20190801]
133. Hur J, West KP, Jr., Shamim AA, et al. Thinness and fecundability: Time to pregnancy after adolescent marriage in rural Bangladesh. *Matern Child Nutr* 2020;16(3):e12985. doi: 10.1111/mcn.12985 [published Online First: 20200324]
134. Hu P, Cai C, Vinturache A, et al. Maternal preconception body mass index and time-to-pregnancy in Shanghai Women, China. *Women Health* 2020;60(9):1014-23. doi: 10.1080/03630242.2020.1784369 [published Online First: 20200701]
135. Alizadeh A, Omani-Samani R, Mansournia MA, et al. Causal Effects of Body Mass Index and Maternal Age on Oocyte Maturation in Assisted Reproductive Technology: Model-Average Causal Effect and Bayesian LASSO Method. *Iran J Public Health* 2020;49(11):2161-69. doi: 10.18502/ijph.v49i11.4734
136. Duan R, Chen Y, Qiao T, et al. Modern dietary pattern is prospectively associated with earlier age at menarche: data from the CHNS 1997-2015. *Nutr J* 2020;19(1):95. doi: 10.1186/s12937-020-00622-z [published Online First: 20200909]
137. Fang Y, Liu J, Mao Y, et al. Pre-pregnancy body mass index and time to pregnancy among couples pregnant within a year: A China cohort study. *PLoS One* 2020;15(4):e0231751. doi: 10.1371/journal.pone.0231751 [published Online First: 20200423]
138. Xu Q, Yang Y, Liu F, et al. Preconception Hb concentration with risk of spontaneous abortion: a population-based cohort study in over 3·9 million women across rural China. *Public Health Nutr* 2020;23(16):2963-72. doi: 10.1017/s1368980019003811 [published Online First: 20200305]
139. Ou H, Yu Q. Efficacy of aspirin, prednisone, and multivitamin triple therapy in treating unexplained recurrent spontaneous abortion: A cohort study. *Int J Gynaecol Obstet* 2020;148(1):21-26. doi: 10.1002/ijgo.12972 [published Online First: 20191105]
140. Mao YY, Yang L, Li M, et al. Periconceptional Folic Acid Supplementation and the Risk of Spontaneous Abortion among Women Who Prepared to Conceive: Impact of Supplementation Initiation Timing. *Nutrients* 2020;12(8) doi: 10.3390/nu12082264 [published Online First: 20200729]
141. Madlala HP, Malaba TR, Newell ML, et al. Elevated body mass index during pregnancy and gestational weight gain in HIV-infected and HIV-uninfected women in Cape Town, South Africa: association with adverse birth outcomes. *Trop Med Int Health* 2020;25(6):702-13. doi: 10.1111/tmi.13387 [published Online First: 20200323]
142. Madzorera I, Isanaka S, Wang M, et al. Maternal dietary diversity and dietary quality scores in relation to adverse birth outcomes in Tanzanian women. *Am J Clin Nutr* 2020;112(3):695-706. doi: 10.1093/ajcn/nqaa172
143. Kutchi I, Chellammal P, Akila A. Maternal Obesity and Pregnancy Outcome: in Perspective of New Asian Indian Guidelines. *J Obstet Gynaecol India* 2020;70(2):138-44. doi: 10.1007/s13224-019-01301-8 [published Online First: 20200113]
144. Ali SA, Tikmani SS, Saleem S, et al. Hemoglobin concentrations and adverse birth outcomes in South Asian pregnant women: findings from a prospective Maternal and Neonatal Health Registry. *Reprod Health* 2020;17(Suppl 2):154. doi: 10.1186/s12978-020-01006-6 [published Online First: 20201130]
145. Mali RV, Dalal A, Khursheed R, et al. Association of Stillbirths with Maternal and Fetal Risk Factors in a Tertiary Care Hospital in South India. *Obstet Gynecol Int* 2021;2021:8033248. doi: 10.1155/2021/8033248 [published Online First: 20210722]
146. Cai H, Mol BW, Gordts S, et al. Early and late pregnancy loss in women with polycystic ovary syndrome undergoing IVF/ICSI treatment: a retrospective cohort analysis of 21 820 pregnancies. *Bjog* 2021;128(7):1160-69. doi: 10.1111/1471-0528.16590 [published Online First: 20201119]
147. Wu Y, Gu Q, Cui X, et al. Higher poultry consumption was associated with an earlier age at menarche. *Acta Paediatr* 2021;110(3):889-95. doi: 10.1111/apa.15554 [published Online First: 20200916]

148. Gao J, Ma H, Wang Y, et al. Hospital-Based Phenotypic Features and Treatment Outcomes of Chinese Women with Polycystic Ovary Syndrome: The Effect of Body Mass Index and Geographic Distribution. *Engineering (Beijing, China)* 2021;7(2):170-77. doi: 10.1016/j.eng.2020.12.006
149. Wang L, Liang R, Zhang G, et al. Serum zinc concentration and risk of adverse outcomes to in vitro fertilization and embryo transfer: A prospective cohort study in northern China. *Sci Total Environ* 2021;792:148405. doi: 10.1016/j.scitotenv.2021.148405 [published Online First: 20210610]
150. Chaurasia AKD, Lata Shukala. A Study to Determine the Early Pregnancy BMI, the Prevalence of Various Levels of BMI, and the Correlation in Order to Analyse the Influence of Low Weight, Overweight, and Obesity on Mother and Foetal Outcomes. *International Journal of Pharmaceutical and Clinical Research* 2021;13(3):424-32.
151. Ambedkar D, Mishra C, Sharma R, et al. Antepartum and Intrapartum Complications in Anemic and Non-Anemic Women. *European Journal of Molecular & Clinical Medicine (EJMCM)* 2021;8(04):2021.
152. Yang J, Luo G, Chen X. Individualized Supplement of Folic Acid Based on the Gene Polymorphisms of MTHER/MTRR Reduced the Incidence of Adverse Pregnancy Outcomes and Newborn Defects. *Niger J Clin Pract* 2021;24(8):1150-58. doi: 10.4103/njcp.njcp_381_20
153. Yang AM, Xu X, Han Y, et al. Risk Factors for Different Types of Pregnancy Losses: Analysis of 15,210 Pregnancies After Embryo Transfer. *Front Endocrinol (Lausanne)* 2021;12:683236. doi: 10.3389/fendo.2021.683236 [published Online First: 20210625]
154. Yang R, Niu ZR, Chen LX, et al. Analysis of related factors affecting cumulative live birth rates of the first ovarian hyperstimulation in vitro fertilization or intracytoplasmic sperm injection cycle: a population-based study from 17,978 women in China. *Chin Med J (Engl)* 2021;134(12):1405-15. doi: 10.1097/cm9.0000000000001586 [published Online First: 20210604]
155. Tang J, Zhu X, Chen Y, et al. Association of maternal pre-pregnancy low or increased body mass index with adverse pregnancy outcomes. *Sci Rep* 2021;11(1):3831. doi: 10.1038/s41598-021-82064-z [published Online First: 20210215]
156. Qu P, Yan M, Zhao D, et al. Association Between Pre-Pregnancy Body Mass Index and Miscarriage in an Assisted Reproductive Technology Population: A 10-Year Cohort Study. *Front Endocrinol (Lausanne)* 2021;12:646162. doi: 10.3389/fendo.2021.646162 [published Online First: 20210616]
157. Hu CY, Yang XJ, Hua XG, et al. Risk factors for spontaneous abortion from a prevention perspective in rural China: a population-based follow-up study. *J Matern Fetal Neonatal Med* 2021;34(16):2583-91. doi: 10.1080/14767058.2019.1670160 [published Online First: 20191001]
158. Chen Y, Wan K, Gong Y, et al. Assessing the relationship between pregravid body mass index and risk of adverse maternal pregnancy and neonatal outcomes: prospective data in Southwest China. *Sci Rep* 2021;11(1):7591. doi: 10.1038/s41598-021-87135-9 [published Online First: 20210407]
159. Bakleicheva M, Bespalova O, Kovaleva I. Features of the 1st trimester of pregnancy course with severe deficiency of 25(OH)D. *Gynecol Endocrinol* 2021;37(sup1):49-53. doi: 10.1080/09513590.2021.2006527
160. Jessani S, Saleem S, Hoffman MK, et al. Association of haemoglobin levels in the first trimester and at 26-30 weeks with fetal and neonatal outcomes: a secondary analysis of the Global Network for Women's and Children's Health's ASPIRIN Trial. *Bjog* 2021;128(9):1487-96. doi: 10.1111/1471-0528.16676 [published Online First: 20210412]
161. Unisa S, Negi K, Pujari S, et al. Do dietary patterns and morbidities have a relationship with primary infertility among women? A study from NFHS-4 (2015-16), India. *J Biosoc Sci* 2022;54(4):682-97. doi: 10.1017/s0021932021000274 [published Online First: 20210618]
162. Li R, Mai T, Zheng S, et al. Effect of metformin and exenatide on pregnancy rate and pregnancy outcomes in overweight or obese infertility PCOS women: long-term follow-up of an RCT. *Arch Gynecol Obstet* 2022;306(5):1711-21. doi: 10.1007/s00404-022-06700-3 [published Online First: 20220713]
163. Wu S, Zhang X, Zhao X, et al. Preconception Dietary Patterns and Associations With IVF Outcomes: An Ongoing Prospective Cohort Study. *Front Nutr* 2022;9:808355. doi: 10.3389/fnut.2022.808355 [published Online First: 20220216]
164. Zhang Y, Jukic AMZ, Song H, et al. Serum Vitamin D Concentrations, Time to Pregnancy, and Pregnancy Outcomes among Preconception Couples: A Cohort Study in Shanghai, China. *Nutrients* 2022;14(15) doi: 10.3390/nu14153058 [published Online First: 20220726]

165. Zhu C, Yan L, He C, et al. Incidence and risk factors of infertility among couples who desire a first and second child in Shanghai, China: a facility-based prospective cohort study. *Reprod Health* 2022;19(1):155. doi: 10.1186/s12978-022-01459-x [published Online First: 20220708]
166. Xiong J, Xu Y, Liu X, et al. Prospective association of dietary soy and fibre intake with puberty timing: a cohort study among Chinese children. *BMC Med* 2022;20(1):145. doi: 10.1186/s12916-022-02320-5 [published Online First: 20220404]
167. Xu Y, Xiong J, Gao W, et al. Dietary Fat and Polyunsaturated Fatty Acid Intakes during Childhood Are Prospectively Associated with Puberty Timing Independent of Dietary Protein. *Nutrients* 2022;14(2) doi: 10.3390/nu14020275 [published Online First: 20220110]
168. Chai J, Guo T, Deng Y, et al. Preconception alcohol consumption and risk of miscarriage in over 4.5 million Chinese women aged 20-49 years. *BMJ Sex Reprod Health* 2022;48(e1):e53-e59. doi: 10.1136/bmjsrh-2020-201012 [published Online First: 20210510]
169. Zheng Y, Dong X, Chen B, et al. Body mass index is associated with miscarriage rate and perinatal outcomes in cycles with frozen-thawed single blastocyst transfer: a retrospective cohort study. *BMC Pregnancy Childbirth* 2022;22(1):118. doi: 10.1186/s12884-022-04443-2 [published Online First: 20220211]
170. Shi H, Chen L, Wang Y, et al. Severity of Anemia During Pregnancy and Adverse Maternal and Fetal Outcomes. *JAMA Netw Open* 2022;5(2):e2147046. doi: 10.1001/jamanetworkopen.2021.47046 [published Online First: 20220201]
171. Chen H, Li J, Cai S, et al. Impact of body mass index (BMI) on the success rate of fresh embryo transfer in women undergoing first in vitro fertilization/intracytoplasmic sperm injection (IVF/ICSI) treatment. *Int J Obes (Lond)* 2022;46(1):202-10. doi: 10.1038/s41366-021-00978-0 [published Online First: 20211009]
172. Wondemagegn AT, Tsehay B, Mebiratie AL, et al. Effects of dietary diversification during pregnancy on birth outcomes in east Gojjam, northwest Ethiopia: A prospective cohort study. *Front Public Health* 2022;10:1037714. doi: 10.3389/fpubh.2022.1037714 [published Online First: 20221205]
173. Perumal N, Wang D, Darling AM, et al. Associations between Gestational Weight Gain Adequacy and Neonatal Outcomes in Tanzania. *Ann Nutr Metab* 2022;78(3):156-65. doi: 10.1159/000522197 [published Online First: 20220204]
174. Caniglia EC, Zash R, Swanson SA, et al. Iron, folic acid, and multiple micronutrient supplementation strategies during pregnancy and adverse birth outcomes in Botswana. *Lancet Glob Health* 2022;10(6):e850-e61. doi: 10.1016/s2214-109x(22)00126-7
175. Kamenju P, Madzorera I, Hertzmark E, et al. Higher Dietary Intake of Animal Protein Foods in Pregnancy Is Associated with Lower Risk of Adverse Birth Outcomes. *J Nutr* 2022;152(11):2546-54. doi: 10.1093/jn/nxac183 [published Online First: 20220818]
176. Kalla A, Loucif L, Yahia M. Miscarriage Risk Factors for Pregnant Women: A Cohort Study in Eastern Algeria's Population. *J Obstet Gynaecol India* 2022;72(Suppl 1):109-20. doi: 10.1007/s13224-021-01564-0 [published Online First: 20211006]
177. Abioye AI, Hughes MD, Sudfeld CR, et al. The effect of iron supplementation on maternal iron deficiency anemia does not differ by baseline anemia type among Tanzanian pregnant women without severe iron deficiency anemia. *Eur J Nutr* 2023;62(2):987-1001. doi: 10.1007/s00394-022-03029-0 [published Online First: 20221108]
178. Yu Z, Sun Y, Wang P, et al. Does vitamin D level associate with pregnancy outcomes in Chinese women undergoing in vitro fertilization/intracytoplasmic sperm injection-embryo transfer? A retrospective cohort study. *J Obstet Gynaecol Res* 2023;49(3):835-45. doi: 10.1111/jog.15521 [published Online First: 20221219]
179. Eldin AB, Ibrahim M, Elsheikh A, et al. Insights into Early Pregnancy Demise following Intracytoplasmic Sperm Injection in Women with Unexplained Infertility. *JBRA Assist Reprod* 2023;27(1):4-8. doi: 10.5935/1518-0557.20220005 [published Online First: 20230330]
180. Gudipally M, Farooq F, Basany K, et al. Impact of prepregnancy body mass index on adverse pregnancy outcomes: analysis from the Longitudinal Indian Family hEalth cohort study. *AJOG Glob Rep* 2023;3(1):100134. doi: 10.1016/j.xagr.2022.100134 [published Online First: 20221105]
